# Serum metabolomics study of narcolepsy type 1 based on ultra-performance liquid chromatography–tandem mass spectrometry

**DOI:** 10.1007/s00726-023-03315-z

**Published:** 2023-09-10

**Authors:** Qingqing Zhan, Lili Wang, Nan Liu, Yuqing Yuan, Liying Deng, Yongmin Ding, Fen Wang, Jian Zhou, Liang Xie

**Affiliations:** 1 Department of Neurology, The Second Affiliated Hospital of Nanchang University, Nanchang, 330006 Jiangxi People’s Republic of China; 2 Institute of Neuroscience, School of Basic Medical Sciences, Chongqing Medical University, 1 Yixueyuan Road, Yuzhong District, Chongqing, 400016 China

**Keywords:** Narcolepsy type 1, Metabolite profile, Serum samples, Principal component analysis, Enrichment pathways

## Abstract

Narcolepsy is a chronic and underrecognized sleep disorder characterized by excessive daytime sleepiness and cataplexy. Furthermore, narcolepsy type 1 (NT1) has serious negative impacts on an individual's health, society, and the economy. Currently, many sleep centers lack the means to measure orexin levels in the cerebrospinal fluid. We aimed to analyze the characteristics of metabolite changes in patients with NT1, measured by ultra-performance liquid chromatography–tandem mass spectrometry. A principal component analysis (PCA), an orthogonal partial least square discriminant analysis (OPLS-DA), *t* tests, and volcano plots were used to construct a model of abnormal metabolic pathways in narcolepsy. We identified molecular changes in serum specimens from narcolepsy patients and compared them with control groups, including dehydroepiandrosterone, epinephrine, N-methyl-D-aspartic acid, and other metabolites, based on an OPLS-loading plot analysis. Nine metabolites yielded an area under the receiver operating curve > 0.75. Meanwhile, seven abnormal metabolic pathways were correlated with differential metabolites, such as metabolic pathways; neuroactive ligand‒receptor interaction; and glycine, serine, and threonine metabolism. To our knowledge, this is the first study to reveal the characteristic metabolite changes in sera from NT1 patients for the selection of potential blood biomarkers and the elucidation of NT1 pathogenesis.

**Supplementary Information:**

The online version contains supplementary material available at 10.1007/s00726-023-03315-z.

## Introduction

Narcolepsy type 1 (NT1) is a chronic neurologic disorder characterized by excessive daytime sleepiness (EDS) that is often profound. NT1 is accompanied by a series of symptoms, such as cataplexy and hypnagogic hallucinations (Richardson et al. 1990; Krahn and Gonzalez-Arriaza 2004; Han 2012). Quality of life studies have shown that the influence of NT1 is similar to or worse than that of Parkinson’s disease or epilepsy, as it can contribute to difficulties in holding a job, psychiatric comorbidities, and cause an increased risk of motor vehicle accidents (Beusterien et al. 1999; Teixeira et al. 2004). In addition to the clinical manifestations and the need for multiple sleep latency test (MSLT), which is an expensive gold standard of NT1 diagnosis (Sateia 2014; Kapur et al. 2017), measurement of cerebrospinal fluid (CSF) hypocretin-1 has been included in the major diagnostic criteria (Bourgin et al. 2008). However, the detection of CSF hypocretin-1 is refused by many Chinese patients given the invasive nature of lumbar puncture. Furthermore, many hospitals cannot test for cerebrospinal fluid orexin. Thus, the discovery of an effective and convenient clinical tool to monitor NT1 would aid in the diagnosis.

In recent years, serum testing has been used extensively in clinical diagnosis. Serum testing is simple to utilize, readily available, less expensive, and objective. Specific serum biomarkers are potentially helpful for NT1 investigations. Assays for the biological marker human leukocyte antigen (HLA) DQB1*0602 are preliminarily performed to diagnose NT1 (Han et al. 2010; Kornum et al. 2017). Nevertheless, this subtype of HLA is very prevalent in the population, and its specificity and sensitivity are relatively lower than those of other detection indices (Luca et al. 2013; Zhang et al. 2018). Studies show that 5–38% of the general population is HLA DQB1*06:02 positive, and NT1 occurs in 1/1000 of these positive individuals (Mignot et al. 2001; Tafti et al. 2014). Therefore, HLA measurement is inaccurate in recognizing the occurrence of NT1 and may result in inappropriate diagnostic conclusions. The existing routine blood work renders diagnosis challenging. Consequently, much more research is urgently needed to explore potential biomarkers in the serum of NT1 patients.

At present, omics data analysis has developed remarkably in large patient populations (Reel et al. 2021). Metabolomics, directly reflecting the molecular phenotype of species, has the potential to discover novel diagnostic markers for disease classification, severity determination, and personalized treatment (Bujak et al. 2015; Wishart 2019). In the present study, we attempted to identify more potential biomarkers in the sera of patients with a diagnosis of NT1 and matched healthy individuals. Serum metabolome alterations and metabolic pathways associated with NT1 risk were analyzed with liquid chromatography‒mass spectrometry (LC‒MS). This study not only provides basic data regarding the mechanism of NT1 patients' metabolic process but also investigates the potential diagnostic values of specific biomarkers of NT1.

## Materials and methods

### Clinical participants

The study protocol was approved by the Ethics Committee of the Second Affiliated Hospital of Nanchang University and was conducted per the ethical principles of the Declaration of Helsinki and the International Conference on Harmonization Good Clinical Practice guidelines. All participants signed an institutional review board-approved informed consent document. During the study, serum samples were obtained from patients who underwent therapy for NT1 at the Second Affiliated Hospital of Nanchang University between November 2018 and May 2020, and these patients were drug-naïve for related medications prior to blood collection. According to the third edition of the International Classification of Sleep Disorders (ICSD-3), in total, 10 subjects were diagnosed with NT1. The criteria include (1) daily periods of an irrepressible need for sleep or daytime lapses into sleep occurring for ≥ 3 months and (2) the presence of the following: cataplexy and positive MSLT findings; mean sleep latency (MSL) ≤ 8 min on the MSLT and ≥ 2 sleep-onset rapid eye movement periods (SOREMPs) on the MSLT; and preceding polysomnography (PSG). In the healthy control (HC) group, 10 age-, sex-, and body mass index (BMI)-matched individuals were recruited; these subjects did not develop any sleep disorders and received a routine health examination at the hospital during the concurrent period.

### Serum collection

Venous blood samples were collected from the 10 NT1 patients and 10 HC participants after an overnight fast. After clotting for 30 min at room temperature, serum samples were collected, followed by refrigerated centrifugation at 1600 × *g* for 10 min. The sera were cryopreserved at − 80 °C within 2 h of collection until further analyses were performed.

### Sample preparation

Frozen serum samples were lyophilized in a 1:1 proportion. We mixed the lyophilized powder with 1 mL methanol (− 20 °C) and vortexed the mixture for 1 min. Then, 450 µL supernatant was removed after centrifugation (12,000 rpm, 4 °C, 10 min) and concentrated to dryness using a vacuum centrifuge. The samples were dissolved in 150 μL of 2-chlorobenzalanine (4 ppm) 80% methanol solution, and the supernatants were filtered through a 0.22 μm filter membrane for the LC/MS analysis. Meanwhile, a quality control (QC) sample was prepared by mixing 20 μL of each sample to evaluate the data quality and correct for variations in the analytical instrument.

### LC‒MS analysis

Chromatographic separation was performed with a Thermo Ultimate 3000 system using an ACQUITY UPLC® HSS T3 (150 × 2.1 mm, 1.8 μm, Waters) column. The temperatures of the column oven and autosampler were set at 40 °C and 8 °C, respectively. Gradient elution was carried out with mobile phases A (5 mM ammonium formate in water) and B (acetonitrile) or C (0.1% formic acid in water) and D (0.1% formic acid in acetonitrile). The flow rates of the mobile phases were 0.25 mL/min, and the volume of injection was 2 μL after equilibration.

The MS analysis was executed on a Thermo Q Exactive mass spectrometer with an ESI source. The electrospray ionization voltage was 3.8 kV in the positive ion mode and 2.5 kV in the negative ion mode, and the capillary temperature was 325 °C. The full MS scan parameters were as follows: m/z range 81–1000 and resolution 70,000. For the HCD scans, the normalized collision energy was 30 eV. Dynamic exclusion was implemented to remove some unnecessary information in the MS/MS spectra.

The raw data files were converted into mzXML format with ProteoWizard (v3.0.8789). Peak identification, peak filtration, and peak alignment of each metabolite were performed using the R (v3.3.2) package XCMS to obtain the mass-to-charge ratio (m/z), retention time, intensity, and positive and negative precursor molecules. Then, the peak intensities were batch-normalized to the total spectral intensity. The molecular formulae (molecular formula error < 20 ppm) were assessed, and the peaks were matched with Metlin (https://mona.fiehnlab.ucdavis.edu//) to confirm the annotations of the metabolites.

### Data processing and statistical analysis

Comparisons of clinical information between the HC and NT1 groups were performed using Fisher’s exact test for sex and HLA–DQB1*06:02, Mann–Whitney rank sum test for age and Epworth Sleepiness Scale (ESS) score variables, and independent-samples *t* test for BMI (kg/m^2^). All statistical analyses were carried out using SPSS version 19 (IBM Corporation, Armonk, NY, USA). A correlation study of the patients’ clinical indicators was carried out with R (corrplot package). The global trends and aggregate state in the two groups among all samples were investigated through an unsupervised principal component analysis (PCA). A supervised orthogonal partial least squares discriminant analysis (OPLS-DA) was used to maximize the metabolic alteration and find significantly changed metabolites between the NT1 and control groups. Further permutation experiments were performed to evaluate the accuracy of the OPLS-DA model. The relative importance of each variable is represented by the variable importance in the projection (VIP) values, and VIP ≥ 1 was considered significant. Finally, the significant differences between the NT1 and control groups were evaluated using independent (unpaired) samples *t* tests. The results are presented as the mean ± standard deviation, and *P* values < 0.05 were considered to indicate statistically significant differences. Volcano plots were generated with R to assess the differential metabolites based on two indicators, i.e., VIP and *P* value. Heatmaps and a clustering analysis of the differential metabolites were also performed with the R package (www.r-project.org). We conducted a receiver operating characteristic (ROC) curve analysis with the survival analysis module to evaluate the diagnostic accuracy of the differentially expressed metabolites between the HC and NT1 groups. Meanwhile, a pathway analysis of the metabolites was carried out with the KEGG pathway database.

## Results

### Study population characteristics

Ten NT1 patients (7 men and 3 women; age range, 10–15 years) and 10 HCs (6 women and 4 men; age range, 10–17 years) were included. There was no significant difference in age or BMI (*p* > 0.05) (Table 1). NT1 patients reported EDS and cataplexy (100%), hypnagogic hallucinations (40%), sleep paralysis (30%), and disturbed nocturnal sleep (90%). The mean age at onset of first symptom (either sleepiness or cataplexy) was 9.80 (2.62) years, and the mean disease duration was approximately 2.92 (2.54) years. MSLT showed a mean sleep latency (mSL) of 2.64 (1.88) min with 4.30 (1.06) SOREMPs. Nocturnal PSG showed a mean total sleep time (TST) of 488.95 (42.38) min with a mSL of 3.95 (4.16) min, sleep efficiency of 83.12 (11.38)%, SOREMPs of 50%, mean N1 stage sleep ratio of 22.77 (12.46), mean N2 stage sleep ratio of 36.78 (10.00), mean N3 stage sleep ratio of 20.70 (5.98), and mean REM stage sleep ratio of 19.76 (7.34).

**Table 1:** Main clinical and biological characteristics of narcolepsy type 1 (NT1) patients and matched healthy controls

Characteristic	Narcolepsy type 1	Healthy control (HC)	*P* value
	(NT1) patients (*N = *10)	participants (*N = *10)	
Sex (male/female)	7/3	6/4	/
Age (years)	12.50 (11.75, 14.25)	12 (11, 13)	0.33
BMI (kg/m^2^)	22.39 ± 4.99	21.50 ± 2.64	0.62
ESS score	15.50 (12.50, 18.50)	1.00 (0, 2.25)	< 0.001
HLA–DQB1*06:02	9* (*N = *9)	0 (*N = *10)	< 0.001

Normally and non-normally distributed data are expressed as mean ± standard deviation or median (interquartile range), respectively.*One patient was not tested for the gene.

**Fig. 1:**
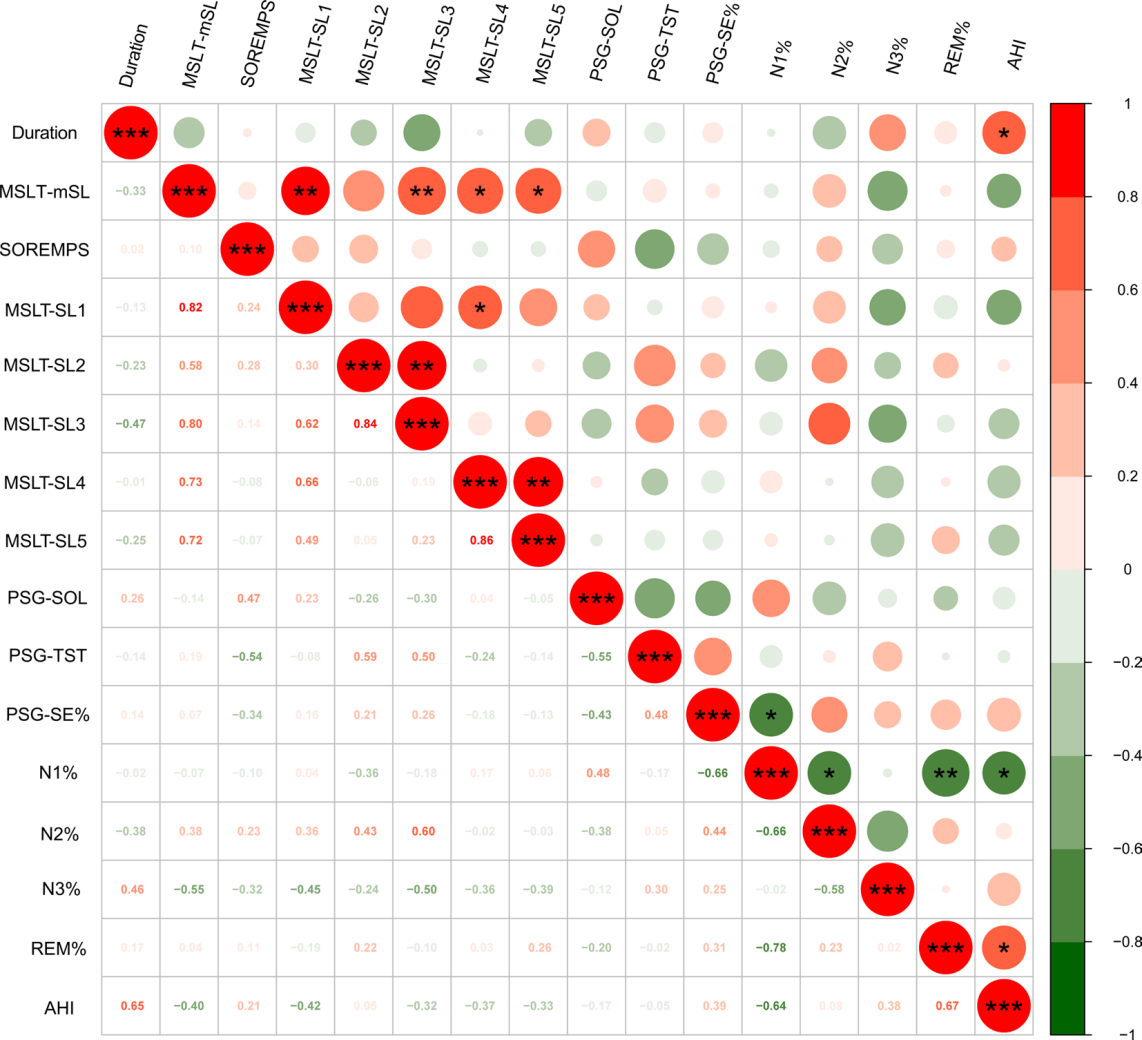
Correlation study of clinical indicators in sera from patients with narcolepsy type 1 (NT1). Significant data points (*P < *0.05) are highlighted in red representing positive correlations and in green, representing negative correlations. The size of the dots denotes the *P* value of correlation; color indicates magnitude of correlation (*P < 0.05, **P < 0.01, ***P < 0.001)

In addition, a correlation analysis of clinical indicators was performed in the NT1 group (Fig. 1). Positive correlations were found between mSL and the first sleep latency (SL1) in MSLT (*r = *0.815, *p = *0.004) and the third sleep latency (SL3) in MSLT (*r = *0.796, *p = *0.006). Meanwhile, negative correlations were found between the proportion of stage N1 (N1%) and the proportion of stage REM (REM%) (*r = − *0.781, *p = *0.008). From the results, it can be reasonably concluded that the percent of TST spent in stage 1 of non-rapid eye movement (NREM) sleep (N1) was significantly reduced, while the REM sleep percentage increased accordingly (Scammell 2015). Meanwhile, we found a correlation between mSL and SL1/3. Although this is an interesting finding, further exploration is needed to arrive at a meaningful conclusion.

### Metabolites expressed in the NT1 group and control group

We first performed qualitative and quantitative analyses of all metabolites in the subjects based on the data pretreatment of metabolomics technology. To reveal the metabolites as comprehensively as possible, metabolomic data were acquired in both the positive and negative ionization modes. In total, 1311 known metabolites were detected in the positive ion mode, and 863 known metabolites were quantified in the negative ion mode. Elaborate information regarding the identified metabolites and lipids in each individual is shown in Supplementary Tables 1 and 2.

**Fig. 2:**
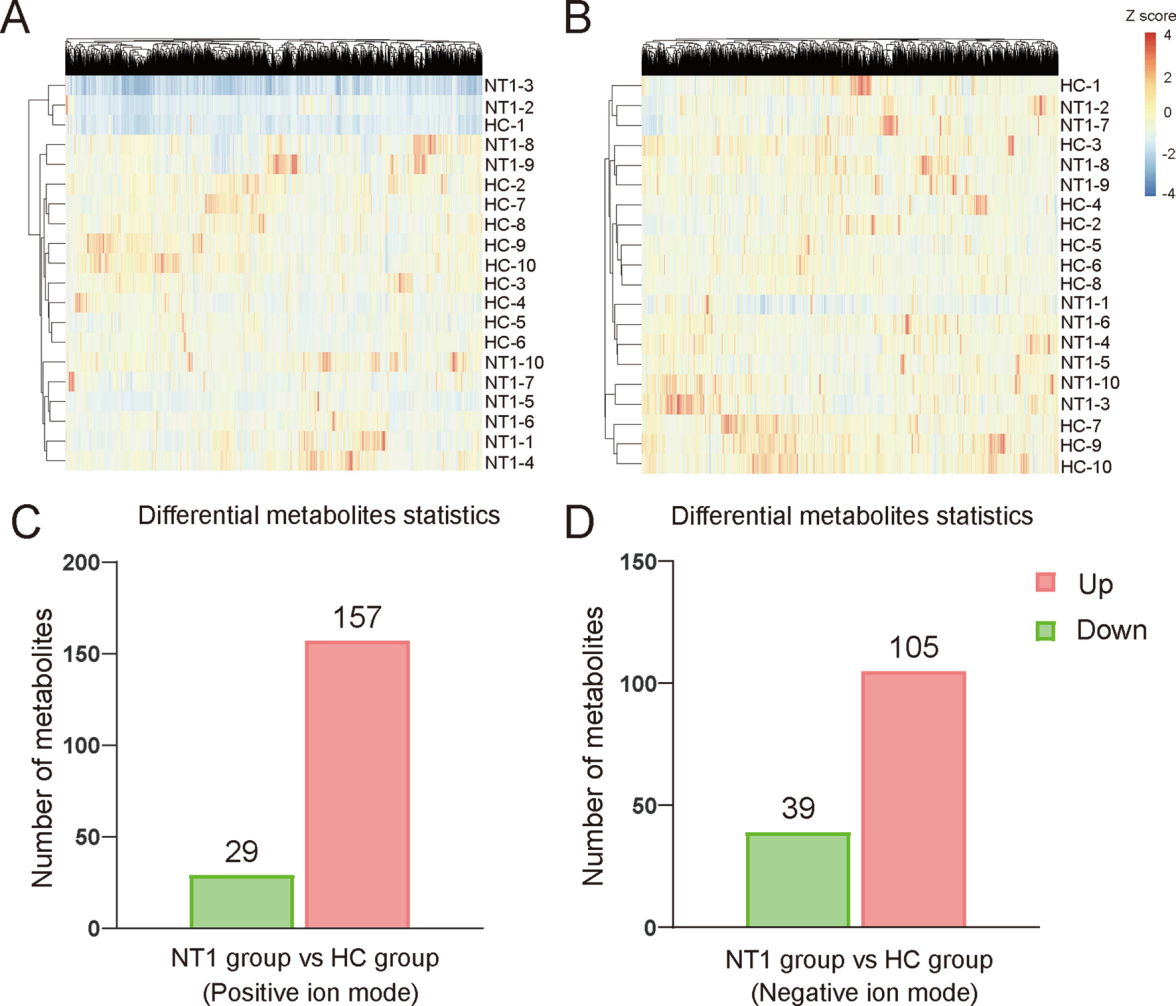
Heatmap of serum discriminating metabolites between narcolepsy type 1 (NT1) patients and healthy controls with their trend of variation in the positive **A** and negative **B** ion modes. Greater intensities of red and blue indicate higher positive or negative correlations, respectively. **C**, **D** Differential serum metabolite histograms of ion modes (red indicates upregulated metabolites, and green represents downregulated metabolites)

To estimate the rationality of the metabolites and more intuitively explain the relationship between the samples and the different expression patterns of metabolites, hierarchical clustering was performed based on the expression in the metabolites. Figure 2A, B shows the hierarchical cluster analysis of all metabolites between the two groups in the positive and negative ion modes. These results show that the serum metabolites between the NT1 and HC groups have a certain degree of cluster trends.

The differential metabolites between the two groups were analyzed by Student’s *t* test (*P < *0.05). Compared with the HCs, in the NT1 group, we identified 186 differential substances, including 157 upregulated and 29 downregulated metabolites, in the positive ion mode. In addition, 144 differential metabolites were identified in the negative ion mode, including 105 upregulated and 39 downregulated metabolites (Fig. 2C, D ). These differential metabolites provided candidate metabolic markers for distinguishing NT1 patients from HCs.

### Identification of differential metabolites in the serum samples of the two groups

**Fig. 3:**
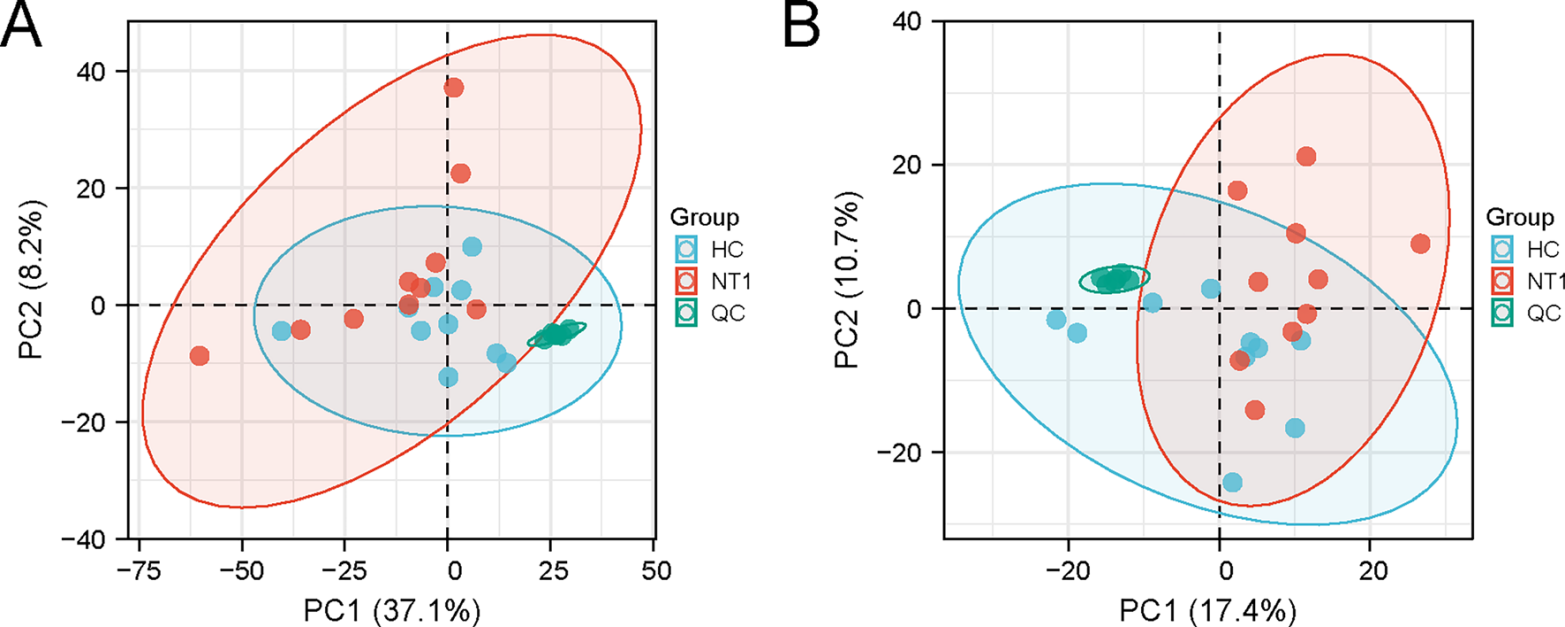
Principal component analysis (PCA) score plot of all samples (red dots: NT1 group; blue dots: HC group) and QC samples (green dots) in the positive **A** and negative **B** ion modes

The quality of the metabolic profiling data was evaluated by performing a principal component analysis of all replicated samples and QC samples. As shown in Fig. 3, the QC samples clustered tightly in both plots of PCA scores, indicating that the stability of the LC/MS analysis was excellent and sufficient to ensure further global metabonomic analysis.

**Fig. 4:**
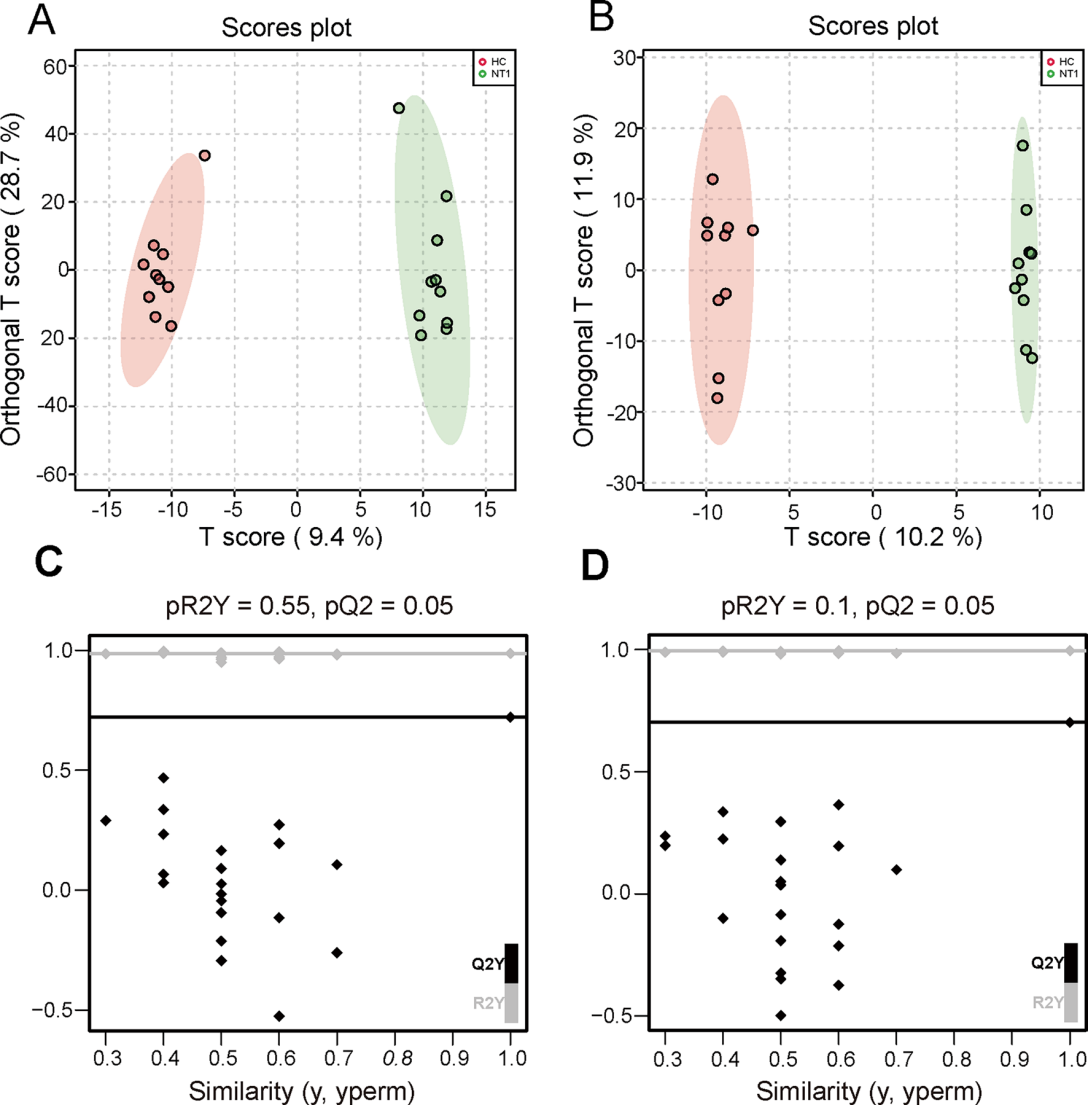
Score plot of OPLS-DA in narcolepsy type 1 (NT1) patients (red) and healthy controls (blue) in the positive **A** and negative **B** ion modes. **C**, **D** Permutation test was performed to assess the reliability of the OPLS-DA model in the two ion modes

To elucidate the variations in serum metabolites and maximize the discriminatory ability of metabolites between the two groups, an OPLS-DA, which is a supervised multivariate data analysis method, was constructed. The OPLS-DA score plot revealed significant separations of metabolomic data between the NT1 group and HC group, which was clearly separated in different regions (Fig. 4A, B). To examine whether our OPLS-DA model analysis was overfitting, we conducted further permutation experiments. The R2Y and Q2 of the permutation test were both less than the original data values, which were placed rightmost in the diagram of the positive (PR2Y = 0.55, PQ2 = 0.05) and negative (PR2Y = 0.1, PQ2 = 0.05) ion modes (Fig. 4C, D). Overall, these results suggest that the OPLS-DA model was reliable and effective in distinguishing NT1 patients from HCs.

**Fig. 5:**
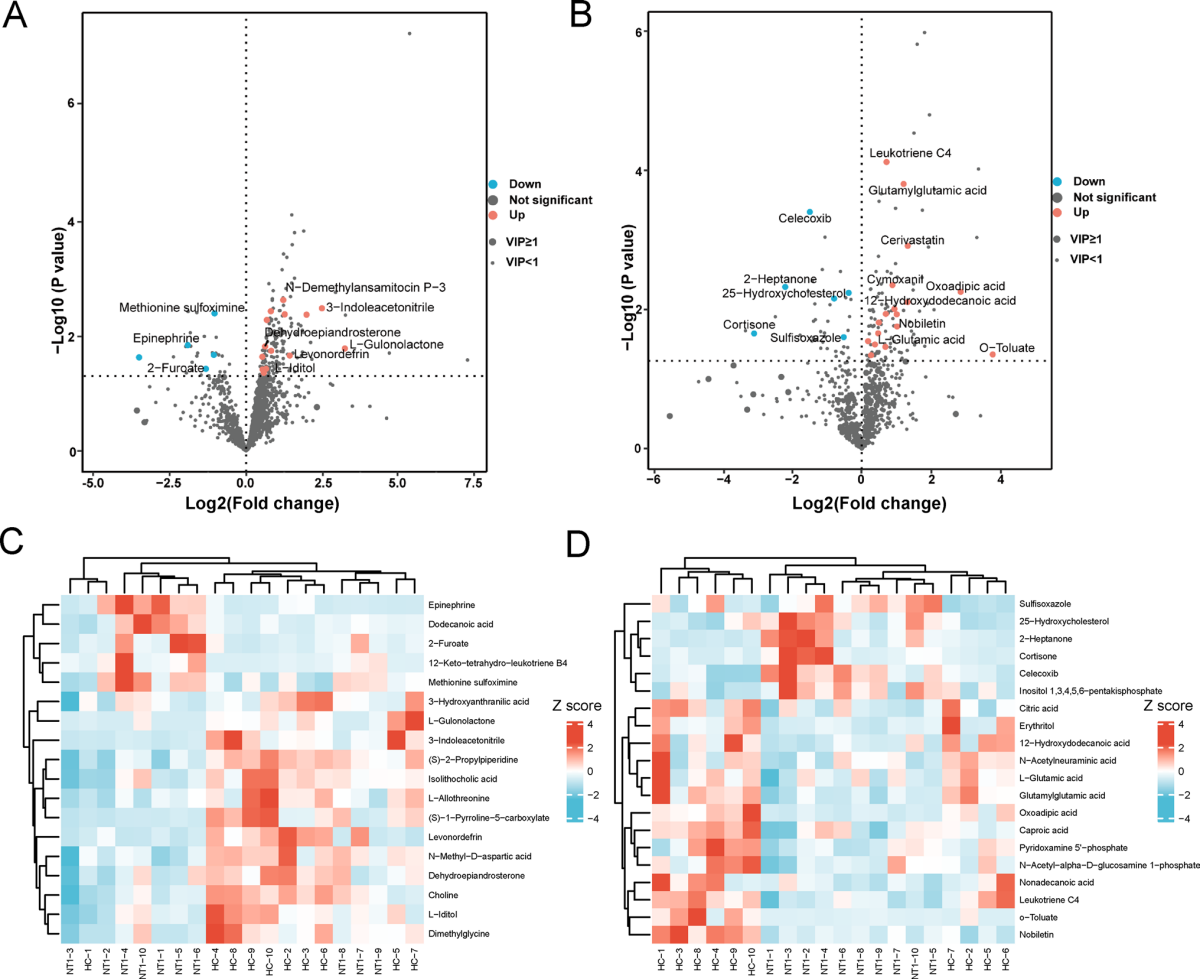
**A**, **B** Volcano plots of differentially expressed metabolic substances between the two groups. Blue represents downregulated metabolites, and red represents upregulated metabolites. **C**, **D** Heatmap showing differential metabolites between the narcolepsy type 1 (NT1) and HC groups

To better understand the differences in metabolites between the NT1 and HC groups, an OPLS-DA model was used to screen the metabolites. Given VIP ≥ 1 and *P < *0.05, 38 metabolites were identified (Table 2). Among them, 27 metabolites showed a meaningful upward trend in the NT1 group, such as dehydroepiandrosterone, citric acid, oxoadipic acid, 3-indoleacetonitrile, and pyridoxamine 5'-phosphate. In contrast, 11 metabolites displayed a significant downward trend, including 2-heptanone, epinephrine, dodecanoic acid, and 25-hydroxycholesterol. Meanwhile, a volcano map (Fig. 5A, B) was drawn based on the VIP values and *t* test findings. The red and blue dots represent up- and downregulated serum metabolites between the two groups, respectively. The differential metabolites were investigated using a hierarchical clustering analysis (HCA) to evaluate the within-group sample similarity. Heatmaps in the positive and negative ion modes are shown in Fig. 5C, D. These data indicate specific patterns of differences in the metabolites between the NTI and HC groups.

**Table 2:** Differential metabolites in the serum of Narcolepsy type 1 (NT1) patients and healthy subjects in positive and negative ion modes

Metabolite name	Retention time (min)	M/z	Formula	VIP	Fold Change	Log2 (Fold Chang)	*P* value	HMDB ESI ±
Dehydroepiandrosterone	12.65	288.2892	C19H28O2	17.29722	1.542134	0.624928	0.015224	HMDB0005962 +
2-Heptanone	5.06	112.9834	C7H14O	11.94813	0.207066	− 2.27184	0.003993	HMDB0003671 −
Epinephrine	1.99	182.9851	C9H13NO3	7.29239	0.264182	− 1.92039	0.014443	HMDB0014344 +
Citric acid	1.27	191.0189	C6H8O7	4.337632	1.423056	0.508992	0.013408	HMDB0000094 −
Oxoadipic acid	2.99	158.9777	C6H8O5	3.063986	7.682051	2.941492	0.004734	HMDB0000225 −
3-Indoleacetonitrile	12.62	156.1202	C10H8N2	2.97338	5.622325	2.491167	0.003297	HMDB0006524 +
Pyridoxamine 5'-phosphate	3.14	248.0534	C8H13N2O5P	2.823978	1.314304	0.394299	0.02838	HMDB0001555 −
L-Gulonolactone	13.88	178.991	C6H10O6	2.781949	9.497233	3.247507	0.016569	HMDB0003466 +
Choline	1.52	104.1072	C5H14NO	2.617989	1.452107	0.538147	0.038437	HMDB0000097 +
o-Toluate	8.98	135.0435	C8H8O2	2.511618	14.8563	3.893003	0.039838	HMDB0002340 −
N-Methyl-D-aspartic acid	1.58	148.0595	C5H9NO4	2.294743	1.602231	0.680082	0.005268	HMDB0002393 +
L-Iditol	3.81	183.0848	C6H14O6	2.169994	1.575525	0.655833	0.037441	HMDB0011632 +
N-Acetylneuraminic acid	1.38	308.0984	C11H19NO9	2.099397	1.631199	0.705933	0.030767	HMDB0000230 −
Erythritol	8.53	121.0283	C4H10O4	1.918096	1.6444	0.717562	0.01002	HMDB0002994 −
Caproic acid	1.31	96.95877	C6H12O2	1.835181	1.140043	0.189089	0.025404	HMDB0000535 −
Dodecanoic acid	2.29	199.9876	C12H24O2	1.791305	0.478383	− 1.06376	0.021372	HMDB0002262 +
25-Hydroxycholesterol	9.23	401.0865	C27H46O2	1.756874	0.765093	− 0.38629	0.004895	HMDB0006247 −
12-Keto-tetrahydro-leukotriene B4	13.99	336.3254	C20H32O4	1.629397	0.087077	− 3.52157	0.023623	HMDB0002995 +
Nobiletin	13.96	402.1298	C21H22O8	1.619453	2.071373	1.050587	0.015374	HMDB0029540 −
L-Allothreonine	1.29	120.0239	C4H9NO3	1.613776	2.408563	1.268173	0.004208	HMDB0004041 +
12-Hydroxydodecanoic acid	12.06	215.165	C12H24O3	1.582275	2.576569	1.365451	0.006711	HMDB0002059 −
Inositol 1,3,4,5,6-pentakisphosphate	1.54	578.8794	C6H17O21P5	1.523154	0.566831	− 0.81901	0.005935	HMDB0003529 −
Cortisone	13.26	341.2691	C21H28O5	1.458127	0.109087	− 3.19645	0.019546	HMDB0015459 −
L-Glutamic acid	1.4	147.0483	C5H9NO4	1.394959	1.404143	0.48969	0.019415	HMDB0060475 −
3-Hydroxyanthranilic acid	5.08	154.0477	C7H7NO3	1.317377	1.445768	0.531836	0.023133	HMDB0001476 +
Sulfisoxazole	3.25	266.0624	C11H13N3O3S	1.312599	0.690648	− 0.53398	0.022133	HMDB0014408 −
Dimethylglycine	1.69	104.0709	C4H9NO2	1.290238	1.7678	0.821955	0.018144	HMDB0000092 +
2-Furoate	2.57	113.0225	C5H4O3	1.277183	0.399548	− 1.32356	0.037463	HMDB0000617 +
Isolithocholic acid	13.5	376.3119	C24H40O3	1.256	1.496682	0.581768	0.043888	HMDB0000717 +
Methionine sulfoximine	1.17	180.9894	C5H12N2O3S	1.250776	0.486647	− 1.03905	0.004054	HMDB0029430 +
(S)-1-Pyrroline-5-carboxylate	3.22	114.055	C5H7NO2	1.23362	3.964764	1.987235	0.004268	HMDB0001301 +
Nonadecanoic acid	12.76	297.2437	C19H38O2	1.194712	2.059567	1.042341	0.010164	HMDB0000772 −
Leukotriene C4	13.87	606.2875	C30H47N3O9S	1.168191	1.667069	0.737314	5.68E-05	HMDB0001198 −
(S)-2-Propylpiperidine	6.19	128.1433	C8H17N	1.099325	1.755246	0.811673	0.003711	HMDB0030285 +
Celecoxib	1.53	362.0566	C17H14F3N3O2S	1.086499	0.343644	− 1.54101	0.000311	HMDB0005014 −
Glutamylglutamic acid	1.29	275.0885	C10H16N2O7	1.078471	2.371192	1.245613	0.00012	HMDB0028818 −
Levonordefrin	1.74	166.0837	C9H13NO3	1.052608	2.70293	1.434524	0.02199	HMDB0015652 +
N-Acetyl-alpha-D-glucosamine 1-phosphate	5.59	300.0457	C8H16NO9P	1.035572	1.213331	0.278973	0.040883	HMDB0001367 −

The metabolites were listed in a decreasing order based on variable importance in the projection values (VIP).

*HMDB* Human metabolome database, *ESI* electrospray ionization.

**Fig. 6:**
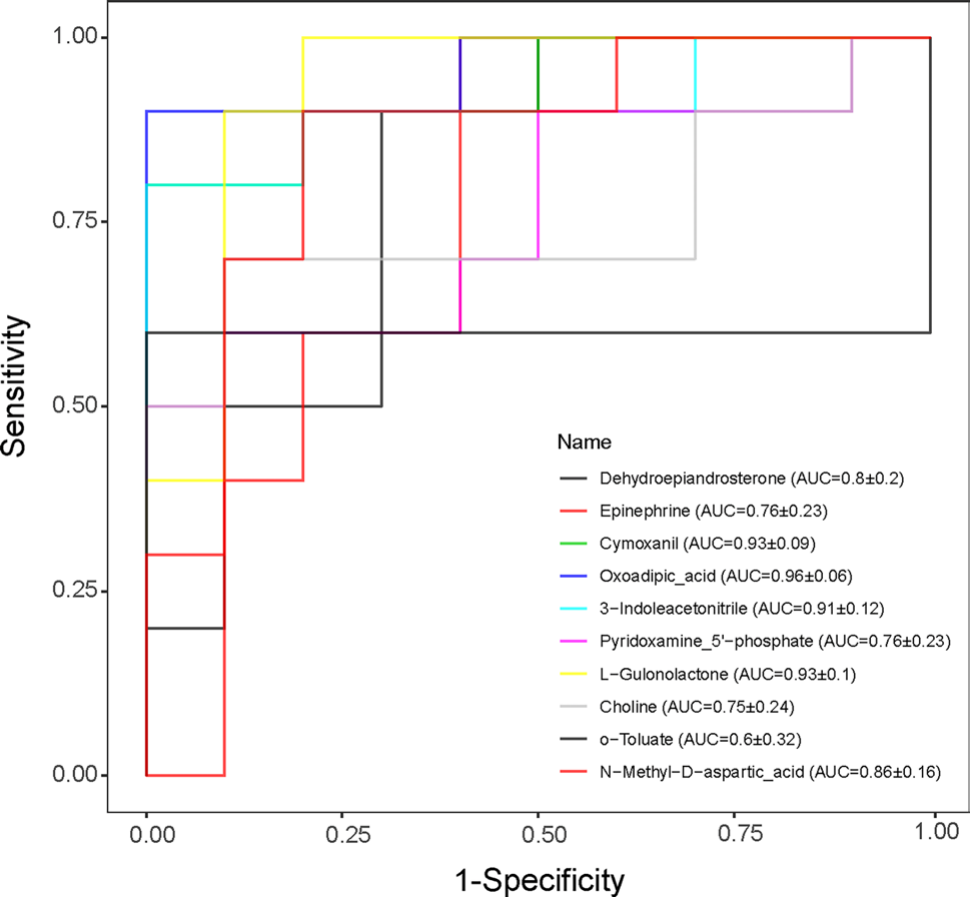
ROC curve analyses of 10 metabolites to evaluate the diagnostic power between narcolepsy type 1 (NT1) vs. healthy controls

Finally, we evaluated the predictive ability of the differential metabolites by plotting ROC curves and comparing the area under the ROC curve (AUC). The AUC values are as indicated: the *X*-axis presents the false-positive rate (1-specificity), and the *Y*-axis presents the true-positive rate (sensitivity). Our results showed that the AUCs of the nine significantly differential metabolites were larger than 0.75, demonstrating that these metabolites had good diagnostic value (Fig. 6). Among them, choline and N-Methyl-D-aspartic acid were also found to be closely related with narcolepsy (Honda et al. 1997; Michinaga et al. 2010). Other metabolites such as cymoxanil, oxoadipic-acid, 3-indoleacetonitrile, and L-gulonolactone had higher diagnostic value with their AUCs larger than 0.9. Moreover, dehydroepiandrosterone, epinephrine, and pyridoxamine 5'-phosphate also have good diagnostic significance.

### Analysis of potential biomarker pathways

**Fig. 7:**
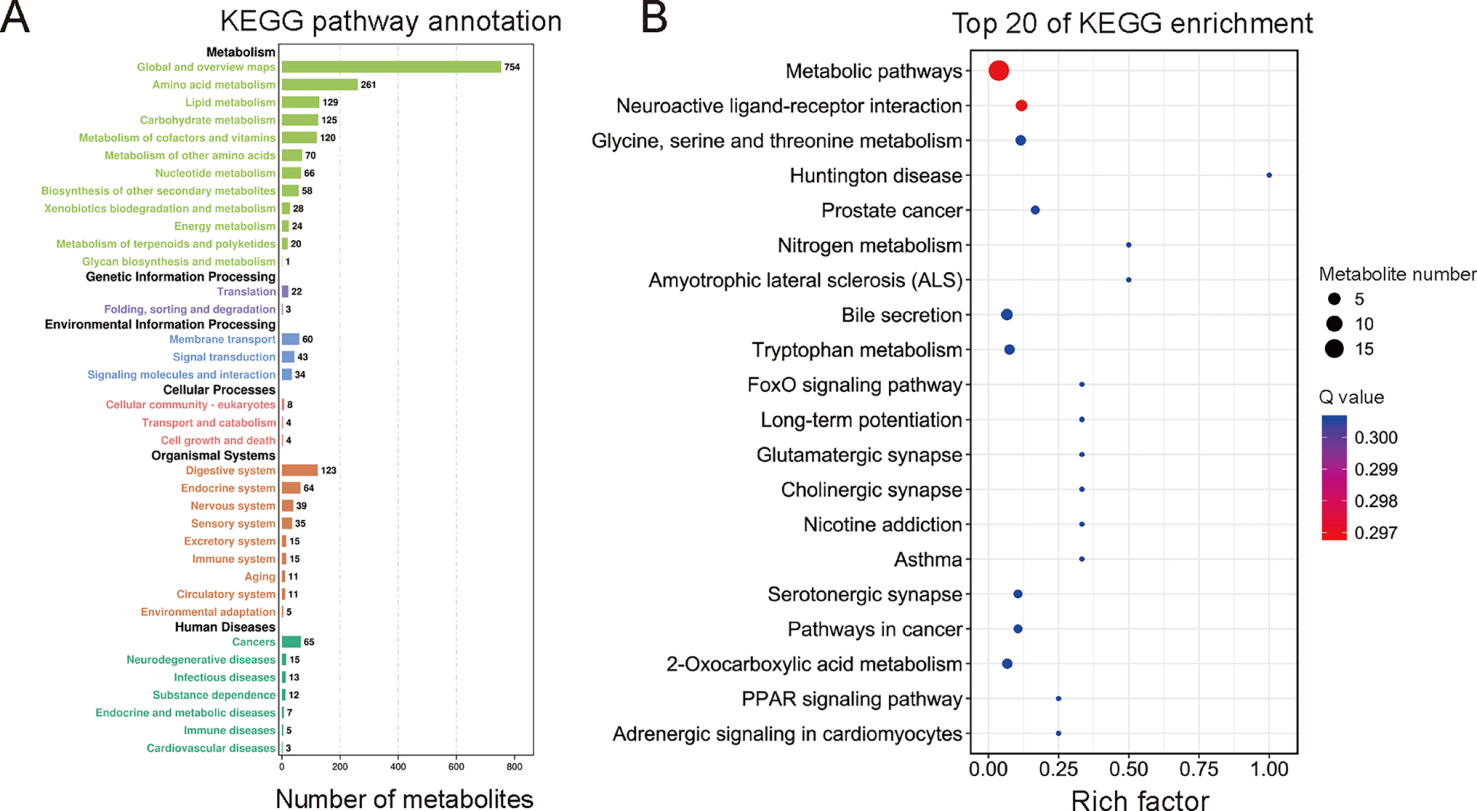
KEGG pathway analysis of differential metabolites associated with narcolepsy type 1 (NT1). **A** Results of the KEGG pathway annotation. **B** Bubble chart of the top 20 enriched KEGG pathways

Next, the differentially expressed metabolites were annotated using the Kyoto Encyclopedia of Genes and Genomes (KEGG) database to identify the metabolic pathways and potential biological functions. These metabolites were enriched in 202 pathways of six KEGG A classes and 33 KEGG B classes. Among the six KEGG A classes, 1656 metabolites were enriched in metabolism, 25 metabolites were enriched in genetic information processing, 137 metabolites were enriched in environmental information processing, 16 metabolites were enriched in cellular processes, 318 metabolites were enriched in organismal systems, and 120 metabolites were enriched in human diseases (Fig. 7A). The top 20 significantly enriched pathways are shown in the KEGG enrichment bubble diagram. The results show that the differentially expressed metabolites were primarily involved in metabolic pathways, neuroactive ligand–receptor interaction, glycine, serine, threonine metabolism, Huntington disease, and prostate cancer (Fig. 7B).

## Discussion

Despite being a rare and underdiagnosed disease, NT1 is known to severely hinder patients' sociality and affect their quality of life. In addition, the underlying pathogenesis of NT1 remains unclear. The untargeted LC–MS-based metabolomics analyses applied in our study not only comprehensively examined metabolites but also revealed a holistic metabolic network. In our current study, we uncovered untargeted metabolic profiles and key metabolic pathways between the general population and NT1 patients. We identified pathway-based metabolomic differences and features of metabolites in NT1 patients through a principal metabolic pathway analysis. These findings could provide a better mechanistic understanding of this complex disease and further insights for early identification and therapeutic intervention.

Our study measured metabolic alterations in NT1 patients and identified 2174 metabolites in serum samples in the positive and negative ion modes. The detected metabolites were compared to those from HC subjects. Dehydroepiandrosterone (DHEA) and N-methyl-D-aspartic acid (NMDA) were significantly increased metabolites in patients with NT1. DHEA is not only the most abundant circulating steroid in the human body but also functions as a neurosteroid (Friess et al. 2000; Maninger et al. 2009). Growing evidence suggests that DHEA supplementation is an effective treatment for adrenal insufficiency, osteoporosis, hypopituitarism, schizophrenia, and depression (Strous et al. 2003; Eser et al. 2006; Maninger et al. 2009). Several studies have shown that sleep efficiency, TST, SL, and daytime dysfunction were not only associated with changed diurnal rhythms of cortisol but also related to alteration in DHEA (Kische et al. 2016; Doan et al. 2018). The hypothalamus–pituitary–adrenal (HPA) axis, two primary outputs of which are cortisol and its antagonist dehydroepiandrosterone (DHEA), is implicated in the pathogenesis of sleep and cardiometabolic disorders (Kische et al. 2016; Doan et al. 2018). The research found that a shorter sleep duration, poorer subjective sleep quality, and lower sleep efficiency were associated with a slower rate of cortisol decline later in the day. The variations in DHEA were similar in direction to those in cortisol (Huang et al. 2017). A recent study found that enhanced levels of DHEA were positively correlated with N3 sleep stage in practitioners of long-term mindfulness meditation (Nagendra et al. 2022). In addition, previous studies have shown that estriol was significantly increased in a group of male patients with NT1. The plasma testosterone level was normal in patients' urine, suggesting that estriol was more likely to be derived from androgens (DHEA and testosterone) through metabolization in the liver rather than from the testicles or the adrenal cortex (Sjaastad et al. 1970). These findings are broadly consistent with our results of the variation trend of DHEA observed in NT1 patients. Moreover, DHEA is a neurosteroid, and clinical trials have consistently found that healthy young males receiving DHEA shortly before bedtime showed enhanced activity in the sigma and theta electroencephalogram (EEG) frequencies and increased REM sleep; whereas, the levels of sleep-associated secretion of human growth hormone, cortisol, and peripheral testosterone were uninfluenced. These results may be related to the mixed agonistic and antagonistic functions of steroids and their metabolites interacting with the GABAA receptor system (Friess et al. 1995, 2000). Another hypothesis is that DHEA is a powerful modulator of sleep states and memory processes that depends on the cholinergic systems in patients with memory disorders. Local infection of DHEA in the brainstem and basal forebrain cholinergic neurons reportedly alter sleep and memory in rodents (George et al. 2006). However, the mechanism of DHEA in NT1 remains unknown. Further studies are required to identify the regulatory mechanisms of DHEA and understand the functions of DHEA in NT1.

N-methyl-D-aspartic acid is another metabolite that showed significant differences between the two groups in the metabolomic analysis. NMDA is an agonist for a class of excitatory amino acid glutamate receptors and is implicated in most neuroexcitatory events in the central nervous system (Paoletti et al. 2013; Bozic and Valdivielso 2015). Meanwhile, the misregulation of NMDA, especially its overexcitation, is connected to numerous neurodegenerative disorders, such as Alzheimer’s and Parkinson’s diseases, and neurotoxicity (Catarzi et al. 2006; Johnson and Kotermanski 2006; Paoletti et al. 2013; Gonzalez et al. 2015; Wang et al. 2017). Growing evidence indicates that NMDA tends to induce sleep-like behavior and has a sedative effect. For example, NMDA, α-amino-3-hydroxy-5-methyl-4-isoxazolepropionic acid (AMPA), and ionotropic glutamate receptors (iGluRs) are significant in inducing sedation and hypnosis under severe stress in chicks (Yamane et al. 2009). Therefore, the sleepiness of narcoleptic patients may be closely related to elevated NMDA levels. Some studies have shown that sleep deprivation might reduce the functionality and expression of glutamate NMDA and AMPA receptors (Kopp et al. 2006; McDermott et al. 2006; Hagewoud et al. 2010). This finding may be related to the fact that glutamate, activating NMDA and non-NMDA subtypes of postsynaptic ionotropic glutamate receptors, stimulates orexin neurons. NT1 is caused by the selective loss of orexin-producing neurons (Li et al. 2002; Katsuki and Akaike 2005). The orexin system is obviously the most important in NT1 (Mahoney et al. 2018; Nepovimova et al. 2019). Currently, the relationship between NMDA and orexin is only tentative, and how NMDA regulates orexin to result in the symptoms of NT1 remains to be elucidated.

In biological systems, signaling pathways play a significant role in the homeostasis and development of organisms. Hence, an analysis of signaling pathways not only helps us gain insights into the pathogenesis of diseases but also allows us to better understand how metabolic changes may play a role in the occurrence of NT1. The KEGG pathway enrichment analysis showed that the differential metabolites are mainly involved in metabolic pathways, such as glycine, serine, and threonine metabolism, and neuroactive ligand–receptor interaction. The glycine, serine, and threonine metabolic pathways are thought to provide the main energy metabolism precursor substance for the tricarboxylic acid (TCA) cycle (Schwartz et al. 1985). The metabolites driving these differences were choline, dimethylglycine, and L-allothreonine. Choline, a direct precursor of acetylcholine, was also found to aggravate cataplexy (Honda et al. 1997). Literature regarding the role of other differential metabolites in narcolepsy is still scarce, and we aim to focus our future research on this topic. Previous studies found the five most closely related metabolic pathways to sleep through a database analysis; these include purine metabolism; glycine, serine, and threonine metabolism; nicotinate; and nicotinamide metabolism (Wang et al. 2019). Similarly, in the cerebrospinal fluid metabolomics study in NT1 patients, the significant metabolic pathways were implicated in glycine, serine, and threonine metabolism (Shimada et al. 2020). In another metabolomic study of plasma samples from narcoleptic patients, the significant metabolic pathways were also implicated in glycine and serine metabolism, tryptophan metabolism, and arachidonic acid metabolism (Dauvilliers et al. 2022). Meanwhile, some studies have demonstrated that neurotransmitter; glycine, serine, and threonine metabolism; and proline and arginine metabolism are significantly changed in chronic paradoxical sleep deprivation (PSD) (Gou et al. 2017; Ma et al. 2018). However, the neuroactive ligand–receptor interaction, which was also a significant enrichment pathway, is a gathering of all ligand receptors related to intracellular and extracellular pathways on the plasma membrane. Among those, many potential receptors are closely related to insomnia (Jin et al. 2021). In addition, the upregulation of neuroactive ligand–receptor interactions through treatment with *G. resinaceum* alcohol extract (GRAE) improves sleep (Chen et al. 2022). As a result, these significantly enriched pathways play very important roles in sleep-related diseases, and the roles of these pathways in NT1 need further investigation. This study provides a theoretical basis and new clues for further studies investigating the treatment mechanism for NT1.

Our study has some limitations. Given the rarity of NT1, our sample size was quite small, and the majority of our NT1 patients were from the same region. Thus, our results may not be generalizable. Second, this was a preliminary study of the serum composition in NT1. Further studies are required to confirm the variations in specific biomarkers found in our study through other advanced techniques. Third, despite the specific expression of serum metabolites found in NT1, we should also analyze patients with other subtypes to ensure the diagnostic potential of differential metabolites. Last, we did not include other body fluids such as cerebrospinal fluid and urine because of economic and technological constraints and only compared the data from literature. In the present study, we found both biomarkers and signaling pathways of efficacy in patients with NT1. These findings might potentially lead to the development of a clinical diagnosis of NT1 and a theoretical basis for the occurrence of the disease.

### Supplementary Information

Below is the link to the electronic supplementary material.

Supplementary file1 (PDF 315 KB)

Supplementary file2 (PDF 298 KB)

## Data Availability

The data analyzed in this study has been presented in the manuscript and in the supplemental material.

## Abbreviations

NT1: Narcolepsy type 1
EDS: Excessive daytime sleepiness
REM: Rapid eye movement
MSLT: Multiple sleep latency test
CSF: Cerebrospinal fluid
HLA: Human leukocyte antigen
LC‒MS: Liquid chromatography‒mass spectrometry
PCA: Principal component analysis
QC: Quality control
OPLS-DA: Orthogonal partial least square discriminant analysis
ESS: Epworth Sleepiness Scale
VIP: Variable influence on prediction
