# Supplementary material for: Serum metabolomics study of narcolepsy type 1 based on ultra-performance liquid chromatography–tandem mass spectrometry
Source: Amino Acids. 2023 Sep 10;55(10):1247–59. doi: 10.1007/s00726-023-03315-z (PMC10689557; doi:10.1007/s00726-023-03315-z)
Supplement: Supplementary file 1 — Supplementary file1 (PDF 315 KB) [file 726_2023_3315_MOESM1_ESM.pdf]

Supplementary Table S1

Name	Log2_FC	Pvalue	Vip
(-)-Guttiferone E	1.652983425	0.009061568	0.298488009
(-)-Jasmonic acid	-2.071966809	0.214912091	0.412215439
(+)-Bebeerine	-1.017073581	0.069900767	0.177850558
(+)-Norephedrine	0.409523497	0.103961782	0.282102932
(+)-Tetrandrine	-0.902279127	0.021378713	0.284054617
(13E)-11a-Hydroxy-9,15-dioxoprost-13-enoic acid	0.243434916	0.553698114	0.054760731
(2'E,4'Z,8E)-Colneleic acid	0.435793634	0.419560299	0.160196877
(2R,3R)-3-Methylglutamyl-5-semialdehyde-N6-lysine	0.619692527	0.114443292	0.354825289
(2S,5S)-trans-Carboxymethylproline	0.420377133	0.198510227	0.446250854
(3S)-3,6-Diaminohexanoate	0.20674874	0.437498603	0.235429607
(3S,5S)-Carbapenam-3-carboxylic acid	-0.499932352	0.563321739	0.250344122
(5-L-Glutamyl)-L-glutamate	1.229727533	0.000374445	0.508308104
(6S)-6-beta-hydroxy-1,4,5,6-tetrahydronicotinamide-adenine dinucleotide	-2.390789425	0.023183764	0.626544766
(R) 2,3-Dihydroxy-3-methylvalerate	0.102034479	0.626145448	0.133209773
(R)-3-Hydroxybutyric acid	0.337326487	0.181569604	0.263547991
(R)-4-Hydroxymandelate	0.044651895	0.903810572	0.008244564
(R)-Higenamine	0.970851836	0.148638436	0.193106458
(R)-mandelic Acid	-0.067153971	0.870071845	0.001483846
(R)-Methylmalonate semialdehyde	-0.429817698	0.257988496	1.252570541
(R)-Phenyllactyl-CoA	-1.621622683	0.022444169	0.220752805
(S)-1-Phenylethanol	-0.304078923	0.27448679	0.631103055
(S)-1-Pyrroline-5-carboxylate	1.987234852	0.004267566	1.233620225
(S)-2-Phenyloxirane	0.879178509	0.057567792	0.201772816
(S)-2-Propylpiperidine	0.811673097	0.003710645	1.099325415
(S)-3-Hydroxydodecanoyl-CoA	0.012190738	0.978800984	0.02471452
(S)-4-Hydroxymandelate	0.656887978	0.078321541	0.79570891
(S)-Abscisic acid	0.690691497	0.236336318	0.106084176
(S)-Coclaurine	1.726209144	0.078437015	0.17968392
(S)-ethylmalonyl-CoA	0.316569479	0.434981343	0.171104192
(S)-Lactate	0.548989176	0.293650344	0.649285944
(S)-Methylmalonic acid semialdehyde	1.228406753	0.107946891	1.318984383
(S)-N-Methylcoclaurine	0.51233589	0.299764147	0.106942641
(S)-Norcoclaurine	-0.1903695	0.74079475	0.061561338
(S)-Reticuline	-0.034779165	0.942228273	0.015507531
(S,E)-Zearalenone	0.527809584	0.247481659	0.468040863
(Z)-4-Hydroxy-6-dodecenoic acid lactone	0.579566477	0.256651789	0.083148454
[6]-Gingerol	0.395779964	0.346122517	0.045950871
[6]-Shogaol	0.496455994	0.243078619	0.184223868
1-(3,4-Dihydroxyphenyl)-1-decene-3,5-dione	0.48483956	0.474065179	0.13924629
1-(3,4-Dihydroxyphenyl)-5-hydroxy-3-decanone	0.065577344	0.848737376	0.032969366

1-(5'-Phosphoribosyl)-5-amino-4-imidazolecarboxamide	0.882477632	0.132347889	0.135172475
1,1-Dimethylbiguanide	0.024865794	0.936843035	0.016732445
1,2,3-Trihydroxybenzene	-0.201665648	0.489303167	0.122478506
1,2-Cyclohexanedione	0.750933995	0.044295875	0.488382853
1,2-Dehydroreticuline	0.161717012	0.615698831	0.104481007
1,3-Dihydro-(2H)-indol-2-one	1.602061779	0.041719052	0.218340443
1,5-Anhydrosorbitol	1.090305527	0.008731737	0.20249411
1,5-Naphthalenediamine	0.35215931	0.146235607	0.55808116
1,7-Dimethyluric acid	0.429625414	0.539634529	0.042878822
1,8-Cineole	0.092612607	0.800019951	0.010456107
1,8-Dinitropyrene	1.076822221	0.029562896	0.32666943
10-Deoxymethynolide	0.335660536	0.162515682	0.553730996
10-Formyldihydrofolate	-1.326911967	0.107916113	0.093493331
10-Hydroxydecanoic acid	0.177270577	0.424422895	0.465385333
10-Nitrolinoleic acid	0.874215878	0.348109763	0.22185101
11-Aminoundecanoic acid	0.297396393	0.22414069	0.208537636
11b-Hydroxyandrost-4-ene-3,17-dione	-0.095920881	0.899988385	0.004203125
11-Dehydro-thromboxane B2	-0.118401032	0.639482284	0.090617991
11-O-Demethyl-17-O-deacetylvindoline	0.636105593	0.107679665	0.277408324
12-Keto-leukotriene B4	-0.283286894	0.54737526	0.075972243
12-Keto-tetrahydro-leukotriene B4	-3.521571218	0.023622519	1.629396658
13-Deoxycarminomycin	0.908114719	0.023640834	0.165891489
13-L-Hydroperoxylinoleic acid	0.209729531	0.726713338	0.044398989
14alpha-Hydroxy-5beta-cholest-7-ene-3,6-dione	-0.161878266	0.775835902	0.266480774
15-Deoxy-d-12,14-PGJ2	0.311807331	0.203064436	0.623813536
16(R)-HETE	0.266497366	0.776750824	0.002998134
16-Methoxy-2,3-dihydro-3-hydroxytabersonine	-0.299579623	0.471810827	0.711996934
16-Methoxytabersonine	-0.424008317	0.576088479	0.050525926
17a-Estradiol	0.433069119	0.087108782	0.291743832
17alpha,21-Dihydroxypregnenolone	1.850092553	0.019109659	0.139016009
17-Hydroxyprogesterone	1.214090584	0.018890291	0.314874417
19(R)-HETE	0.863644158	0.040283542	0.239208922
1-Amino-2-methylanthraquinone	1.916352985	0.117192188	0.098647857
1-Aminocyclopropanecarboxylic acid	0.905546241	0.045163449	0.366985353
1D-1-Guanidino-3-amino-1,3-dideoxy-scyllo-inositol	1.040765566	0.212824422	0.07648986
1-Hexadecanol	-0.087111688	0.813244786	0.218890918
1H-Indole-3-acetamide	0.309687455	0.227576407	0.810611132
1-Hydroxymethylnaphthalene	0.306643658	0.385803828	0.16914667
1-Hydroxypyrene	0.411508679	0.137458933	0.669640877
1-Methylhistidine	0.418273294	0.215172958	0.362407143
1-Methylnicotinamide	0.12751803	0.802888188	0.059317092
1-Methyluric acid	0.92218813	0.014560305	0.134957397
1-Naphthol	0.035500283	0.871782731	0.007464042

1-Palmitoyl-2-(5-keto-6-octenedioyl)-sn-glycero-3-phosphocholine	0.203501193	0.478564399	0.024844451
1-palmitoylglycerophosphocholine	-0.007423692	0.995887088	0.241628254
1-Pyrroline-5-carboxylic acid	0.446396814	0.490478375	0.078967066
2,22-Dideoxy-3-dehydroecdysone	-0.435774644	0.473524595	0.128105196
2',3'-Cyclic CMP	-1.413674383	0.153974193	0.091937086
2,3-Diaminopropionic acid	0.301781017	0.188592053	0.173709963
2,3-Dinor-8-iso prostaglandin F2alpha	1.683534432	0.023950524	0.180081839
2,4-Diaminoanisole	0.696784366	0.052717946	1.310130059
2,4-Dihydroxybenzophenone	0.440538324	0.174360767	0.142487115
2,4-Dinitrotoluene	-1.172188302	0.39198748	0.464565785
2,5-Dihydroxybenzoate	0.348152653	0.185704997	0.076959925
2,6-Diethylaniline	0.770627264	0.034910674	0.199939708
2',6'-Dihydroxy-4'-methoxyacetophenone	0.638420709	0.057551045	0.081672392
21-Hydroxypregnenolone	-0.129959475	0.555284196	0.179728466
24,25-Dihydrolanosterol	-1.055100331	0.357310094	0.095508946
24-Methylenecycloartanol	0.378418937	0.123159907	0.815564607
27-O-Demethylrifamycin SV	-0.351163964	0.446639458	0.197191217
2-Acetamidofluorene	0.308948744	0.480895628	0.038562755
2-Acetolactate	-0.146650278	0.716090933	0.015175288
2-Amino-2-deoxy-D-gluconate	-0.452357447	0.42723327	0.138602029
2-Amino-3-phosphonopropionic acid	0.286370814	0.266964085	0.517587306
2-Aminoacrylic acid	0.266423052	0.581962249	0.07588576
2-Aminobenzenesulfonate	-0.076885305	0.851174524	0.016008125
2-Aminobenzimidazole	1.819170859	0.022157813	0.211052271
2-Aminobenzoic acid	0.036641563	0.868999393	0.02354484
2-Aminomalonate semialdehyde	0.004825521	0.984077959	0.005609834
2-Arachidonoylglycerol	0.386524208	0.37382072	0.153675701
2-Carboxy-D-arabinitol 1-phosphate	0.111338887	0.710569888	0.029471677
2-Chloromaleylacetate	0.447664903	0.314998686	0.101407569
2'-Deamino-2'-hydroxyneamine	-1.966623482	0.044226345	0.285309292
2'-Dehydrokanamycin A	1.770387823	0.112006919	0.117726612
2-Dehydropantoate	0.55999831	0.432682662	0.119432168
2-Deoxyecdysone	0.694746829	0.436932022	0.222164854
2-Furancarboxaldehyde	-0.077177831	0.836472202	0.053268011
2-Furoate	-1.323557727	0.0374632	1.277183408
2-Furoyl-CoA	0.719202183	0.226226422	0.052134421
2-Heptanone	0.773978091	0.072760486	1.271446235
2-Hydroxy-3-(4-hydroxyphenyl)propenoic acid	-0.100577087	0.754747122	0.017633126
2-Hydroxybutyric acid	0.57121147	0.181791464	0.353884929
2-Hydroxyestrone	0.264825667	0.776938398	0.314026187
2-hydroxyflutamide	1.500709068	7.80E-05	0.407456997
2-Hydroxyglutarate	0.280805972	0.3727899	0.127589325
2-Keto-6-acetamidocaproate	0.384256146	0.168057733	0.113051589

2-Ketobutyric acid	0.371195088	0.095512929	1.288998318
2-Keto-glutaramic acid	0.985062336	0.316702502	1.020883945
2-Methoxyestradiol	-0.63031767	0.19943148	0.116062059
2-Methoxyestrone	1.881007024	0.008677352	0.161890397
2-Methylserine	0.632057986	0.036296384	0.984038986
2-Methylthiobenzothiazole	-0.434216	0.50393467	0.107789561
2'-N-Acetylparomamine	0.33827487	0.52568609	0.052975877
2'-O-Methyladenosine	0.52493992	0.15999534	0.306697334
2-Oxo-4-methylthiobutanoic acid	-0.702213848	0.215592215	0.897869605
2-Oxoarginine	-0.447899138	0.401246241	0.472718643
2-Phenylacetamide	0.495724985	0.094230454	2.102897293
2-Phenylethanol	-3.342361589	0.318690915	4.115209764
3-(3,4-Dihydroxyphenyl)pyruvate	0.520193976	0.262023845	0.099587502
3-(4-Hydroxyphenyl)-1-(4-hydroxy-2-methoxyphenyl)-2-propen-1-one	0.214427418	0.435541598	0.041791332
3-(Methylthio)propanoyl-CoA	-0.559863606	0.228735448	0.099932258
3,4-Dihydro-2H-1-benzopyran-2-one	0.335976509	0.14565615	0.545834133
3,4-Dihydrospheroidene	-2.150907739	0.277685918	0.525519163
3,4-Dihydroxybenzaldehyde	1.642288354	0.075604966	0.909190186
3,4-Dihydroxymandelic acid	0.331232989	0.158024236	0.292065225
3,4-Dihydroxymandelic acid	-0.795775264	0.206393653	0.187403597
3,4-Methylenedioxyamphetamine	0.674495549	0.411970412	0.116208036
3,5-Dibromo-L-tyrosine	0.890881219	0.062330049	0.186363354
3,5-Dihydroxy-phenylglycine	0.691716947	0.239698536	0.194680709
3,7-Dimethylquercetin	-0.133256642	0.870001345	0.009339996
3-aci-Nitropropanoate	0.217905168	0.611229783	0.477101792
3-alpha(S)-Strictosidine	-0.906971953	0.309671896	0.173287549
3alpha,11beta-Dihydroxy-5alpha-androstane-17-one	0.010849	0.987367666	0.005542741
3alpha,7alpha-Dihydroxy-12-oxo-5beta-cholanate	-0.388669097	0.255066861	0.385275994
3''-Amino-3''-deoxygentamicin A2	0.399650747	0.321885774	0.355469776
3-Amino-4,7-dihydroxycoumarin	0.360474763	0.142472375	0.220253145
3-Amino-4-hydroxybenzoate	0.35382891	0.398559789	0.192647839
3beta,5beta-Ketotriol	0.18898257	0.807926794	0.159201661
3-Carbamoyl-2-phenylpropionaldehyde	0.329582284	0.178841839	1.056053861
3-Dehydro-2-deoxyecdysone	-0.009247016	0.982636878	0.021128851
3-Dehydroecdysone	-0.544115381	0.077856547	0.168038074
3-Dehydrosphinganine	0.495708798	0.160287643	0.663665037
3'-Demethylstaurosporine	0.541800496	0.066942783	0.16902
3-Hydroxyanthranilic acid	0.531836318	0.023133055	1.317376622
3-Hydroxybenzoic acid	-0.106359246	0.877681311	0.043117076
3-Hydroxyphenylacetic acid	-0.037354926	0.911575366	0.016473259
3-Hydroxypicolinic acid	-0.090278562	0.816995507	0.118653369
3'-Hydroxyropivacaine	3.267279762	0.00434021	0.349342477
3-Indoleacetonitrile	2.491166765	0.003297166	2.973380238

3-Isopropylmalate	-0.305640028	0.551839097	0.134695354
3-Ketosphingosine	0.458250265	0.584674436	0.11932953
3-Methoxy-4-hydroxyphenylglycolaldehyde	0.977225553	0.163232038	0.14074697
3-Methoxyanthranilate	0.654229434	0.033507223	0.158310669
3-Methyl-2-oxovaleric acid	0.12996901	0.779664692	0.07945075
3-Methyladenine	0.167821189	0.697488226	0.756055131
3-Methylcrotonyl-CoA	-1.356046251	0.114083905	0.230310743
3-Methylcrotonylglycine	-0.440165351	0.366315284	0.29995431
3-Methyl-L-tyrosine	0.826333734	0.005457354	0.572600362
3-Methyloxindole	0.397290254	0.115427216	0.244387768
3-Methylthiopropyl-desulfoglucosinolate	1.362563594	0.144604305	0.351242526
3-Methylxanthine	0.14929621	0.497075989	0.042945825
3-O-alpha-Mycarosylerythronolide B	-0.355398998	0.737328358	0.05994674
3-Oxalomalate	0.297650589	0.236977466	0.08891245
3-Phosphoglyceric acid	0.128269149	0.681932956	0.033466998
3-Succinoylpyridine	0.156681004	0.723520399	0.021720912
3-Sulfinoalanine	-0.029000694	0.922680736	0.049714925
3-Sulfinylpyruvic acid	0.462955973	0.084888182	0.113069433
4-(2-Aminophenyl)-2,4-dioxobutanoic acid	0.903072541	0.118009733	0.258349269
4-(Methylnitrosamino)-1-(3-pyridyl)-1-butanone	0.422745496	0.092991988	0.332938711
4,4?-Dichlorobenzophenone	0.523573364	0.478459094	0.156031945
4,4'-Methylene bis(2-methylaniline)	0.833645128	0.122023285	0.100142238
4,4'-Methylene-bis-(2-chloroaniline)	0.719728168	0.258664023	0.071480896
4,4'-Thiodianiline	0.308015785	0.344500443	0.060063264
4,5-Dihydroorotic acid	0.511186383	0.183637289	1.260796532
4a-Carbinolamine tetrahydrobiopterin	0.903741595	0.056472911	0.154713311
4a-Carboxy-4b-methyl-5a-cholesta-8,24-dien-3b-ol	0.580466578	0.353052437	0.393052874
4-Acetamido-2-aminobutanoic acid	0.135019352	0.583823532	0.002279286
4-Amino-2-methyl-5-(phosphooxymethyl)pyrimidine	0.242861475	0.358134174	0.085758618
4-Guanidinobutanoic acid	0.509694975	0.073294957	0.086367834
4-Hydroxy-2-butenoic acid gamma-lactone	0.228115576	0.332360649	0.147189536
4-Hydroxy-2-oxoglutaric acid	0.495356269	0.090177764	0.142504648
4-Hydroxy-5-phenyltetrahydro-1,3-oxazin-2-one	-0.19559423	0.490915369	0.599542475
4-Hydroxycinnamic acid	0.481278333	0.092738611	2.159831168
4-Hydroxycinnamoylagmatine	-0.427086871	0.375371903	0.531556138
4-Hydroxycoumarin	0.390922662	0.095854766	0.191678904
4'-Hydroxyflavanone	-0.394761659	0.627421355	0.042350046
4-Hydroxy-L-glutamate	-0.27312588	0.517859928	0.028399731
4-Hydroxyphenylacetylglutamic acid	-0.089220085	0.796166179	0.096326101
4-Hydroxyphenylglyoxylate	0.462273656	0.499258159	0.344694694
4-Hydroxyphenylpyruvic acid	-0.876502984	0.397442633	0.55934061
4-Imidazolone-5-propionic acid	0.394001138	0.234357955	0.170133619
4-Methoxy-2,2'-bipyrrole-5-carbaldehyde	-0.187484463	0.679521951	0.071851734

4'-Methoxyflavanone	0.672129271	0.217077008	0.124189573
4-Methylaminobutyrate	-0.388637961	0.341869318	0.155217059
4-Methylbenzoic acid	0.929990618	0.008215867	0.120720684
4-Methylbenzyl alcohol	-0.238581224	0.505045849	0.062013793
4-Nitroquinoline N-oxide	-0.294513343	0.634980019	0.047496403
4-Octylphenol	1.007573039	0.042716514	0.29128236
4'-Oxolividamine	-0.332586362	0.293395436	0.097726552
4-Quinolinecarboxylic acid	0.515513432	0.166966966	0.395042981
4-Toluenesulfonamide	0.23838502	0.579345553	0.44988303
5-(Methylthio)-2,3-dioxopentyl phosphate	0.705911615	0.090832129	0.186730762
5,10-Methenyltetrahydrofolic acid	0.942019993	0.010127546	0.160162966
5,6-Dihydro-5-fluorouracil	0.509582215	0.210465501	1.051379522
5,7-Dimethoxyflavone	1.825728614	0.057895564	0.167792105
5-Amino-6-(5'-phosphoribitylamino)uracil	0.266838975	0.342520574	0.172617284
5-Aminopentanoic acid	0.12676776	0.675060259	0.12494253
5-Aminosalicylate	-0.138535427	0.736846927	0.03700653
5a-Pregnane-3,20-dione	-0.280815289	0.666108569	0.034965897
5'-Dehydroadenosine	0.214808358	0.380036722	0.154124552
5'-Deoxy-5-fluorouridine	0.43182673	0.400554295	0.116729298
5'-Deoxyadenosine	0.069260194	0.81412333	0.017209756
5-Formiminotetrahydrofolic acid	-0.435413407	0.567137363	0.076623659
5-Guanidino-3-methyl-2-oxopentanoate	-0.245484101	0.451003432	0.14117385
5-Hydroxyconiferyl alcohol	-0.066308188	0.81970768	0.0815262
5-Hydroxyferuloyl-CoA	-0.67751253	0.214766738	0.131864314
5-Hydroxyindoleacetic acid	0.305141984	0.245228149	0.352550438
5-Hydroxyindoleacetylglycine	0.960903856	0.10877195	0.21258779
5-Hydroxy-L-tryptophan	0.553480932	0.025502353	0.28195347
5-Hydroxylysine	-0.443898086	0.342428015	0.127561587
5-Hydroxymethyl-2-furaldehyde	0.237674463	0.390017642	0.178710602
5-Hydroxypentanoic acid	0.75654932	0.007970445	0.335244475
5-KETE	-0.021247118	0.930155899	0.088238138
5-Methylbarbiturate	0.497347853	0.19593839	0.217444265
5-Methylcytosine	-0.067079282	0.813123357	0.008375023
5-Methylphenazine-1-carboxylate	1.183655296	0.219790729	0.119738376
5-Methyltetrahydrofolic acid	-0.233937877	0.729473574	0.022090737
5-Methylthioadenosine	0.213684195	0.655677238	0.186159821
5-Nitro-2-(3-phenylpropylamino)benzoic acid	0.287343386	0.245977191	0.084645163
5-Oxoavermectin "1b" aglycone	0.557206786	0.252659464	0.168743296
6,8a-Seco-6,8a-deoxy-5-oxoavermectin "1b" aglycone	-0.350436161	0.289761226	0.571148313
6-Acetyl-D-glucose	0.476017641	0.138087384	0.248139045
6-Amino-6-deoxyfutalosine	-0.16069427	0.786796907	0.077822606
6-Aminopenicillanic acid	-0.552818779	0.095858291	0.168872518
6beta-Hydroxytestosterone	0.767981465	0.440254219	0.454201607

6-Hydroxyhexan-6-olide	0.342610596	0.14440016	0.452613452
6-Hydroxymelatonin	1.140823189	0.009830945	0.307774028
6-Keto-prostaglandin F1a	-1.512437756	0.242733607	0.263145372
6-Methoxymellein	-0.015329388	0.973416872	0.000549845
6-Methylmercaptopurine	0.464380996	0.069827268	0.390520534
6-Methylpretetramide	0.177731616	0.769681743	0.029020579
6-O-Methyldeacetylisoipecoside	0.186876277	0.446291677	0.039512883
6-O-Methylnorlaudanosoline	0.51018223	0.209893516	0.113646508
6'-Oxogentamicin X2	0.111671167	0.663597691	0.030106171
6'-Oxolividamine	-0.665998584	0.207101292	0.251745445
6-Paradol	1.319868347	0.202645339	0.135569308
6-Phosphogluconic acid	0.45245524	0.046916073	0.29743192
6-Tuliposide B	0.049330315	0.933209619	0.016688736
7(1)-Hydroxychlorophyllide a	1.136615554	0.009883888	0.484177821
7-Methylguanine	0.402052753	0.096122377	0.21621179
7-Methylxanthine	0.407372922	0.1711055	0.163897249
7-Methylxanthosine	0.231588457	0.579107481	0.079641579
7-Oxodeoxycholate	0.087585581	0.761567311	0.017011297
8,9-DiHETrE	-0.29909908	0.284204202	0.286700617
8,9-EET	0.467526208	0.565108344	0.114708752
8-Anilino-1-naphthalene sulfonate	0.230612318	0.284508783	0.136598335
8-Demethyl-8-(2,3,4-tri-O-methyl-alpha-L-rhamnosyl)tetracenomycin C	1.894007631	0.000148874	0.435506804
8-Demethyl-8-alpha-L-rhamnosyltetracenomycin C	0.683243469	0.062838662	0.169890045
8-Ethyl-12-methyl-3-vinylbacteriochlorophyllide d	0.38759411	0.218395963	0.064742654
8-Hydroxyquinoline	0.212796915	0.404768875	0.072012699
9,10-DHOME	0.012849412	0.975273097	0.046986106
9,10-Dihydroxystearate	-0.028673259	0.931814241	0.050849731
9,12,13-TriHOME	0.923437879	0.013416185	0.759279944
9-cis-Retinol	0.657842296	0.410046781	0.318032964
9-Riburonosyladenine	1.718393998	0.006231516	0.214189468
9S-hydroxy-11,15-dioxo-5Z,13E-prostadienoic acid	4.709486253	0.019899492	0.142914212
A alpha C	0.548449182	0.093612722	0.364102978
Acephate	0.372816055	0.130504592	0.250590435
Acetaminophen	0.23417441	0.344765454	0.07845746
Acetazolamide	0.383672548	0.526975512	0.16118542
Acetophenone	0.316074885	0.149603303	0.210433716
Acetylcysteine	0.1713284	0.537768166	0.10938062
Acetylphosphate	0.246733938	0.6619524	0.364111601
Aconitine	0.300977583	0.46305609	0.059057962
Adenosine	0.280878013	0.46682526	0.096185295
Adenosine 5'-phosphate disodium	0.620712779	0.084591138	0.325274326
Adenosine phosphosulfate	0.603185978	0.149822637	0.112610499
Adipate semialdehyde	1.184930619	0.008066046	0.633293177

Aflatoxin B2	-0.309649614	0.344684783	0.246773843
Aflatoxin G	1.008530911	0.337380871	0.049490128
Afzelechin	0.066948471	0.826542665	0.012638005
Agnuside	-0.235192053	0.683822106	0.019340652
Ajmaline	-0.63852634	0.263937644	0.096566076
Alachlor	-1.084728508	0.064795273	0.256615299
Alanine	-0.081465618	0.946492708	0.122095918
Alantolactone	0.842942875	0.202596474	0.237294577
Albendazole	0.332132889	0.313992436	0.074496752
Allantoin	0.153012244	0.643956061	0.013095434
Allocholic acid	1.872731306	0.262590971	0.092418192
Allocystathionine	0.737157526	0.01825399	0.24016186
Allopregnanolone	1.357740937	0.231224591	0.132783602
all-trans-Retinoic acid	0.811418045	0.480712078	0.043894244
Aloesin	0.609559546	0.156935269	0.058592206
alpha-Chaconine	-0.296269041	0.321002343	0.19884453
alpha-Ergocryptine	1.214346215	0.002171131	0.840964633
Alpha-Lactose	0.551575667	0.114305706	0.197704638
alpha-Naphthyl-beta-D-glucuronide	0.536071479	0.034430236	0.235792236
alpha-Solanine	0.357569724	0.165158095	0.097803302
alpha-Tocopherol	0.077460101	0.737656839	0.089114676
Alpha-Tocotrienol	0.490092677	0.236922858	0.293495321
alpha-Zearalenol	-0.475595249	0.302031797	0.281660733
Ametryn	-0.207490104	0.505574755	0.104188127
Amidosulfuron	-0.239990285	0.681216998	0.057356697
Amifostine	0.210977893	0.768499128	0.103966749
Amikacin	-0.692671563	0.402743601	0.218759815
Aminoadipic acid	0.276399063	0.431099142	0.230448188
Aminomalonic acid	-0.92808879	0.060492525	1.241870841
Aminopentol	0.636483791	0.086238583	0.549518916
Aminophenazone	0.245419524	0.620516723	0.047515268
Amiodarone	0.309889546	0.375451905	0.01374373
Amitraz	0.700463599	0.009798152	0.7363861
Ammelide	-0.785329172	0.022456916	0.233723777
AMP	-0.447547623	0.299259616	0.082143188
Ampicillin	0.992233276	0.109082525	0.285950232
Andrographolide	1.109643085	0.226092485	0.126706246
Androsta-1,4-diene-3,17-dione	0.006634978	0.990713657	0.045202043
Androsterone	0.891721106	0.001628151	0.301090509
Angiotensin IV	1.00072763	0.009808364	0.396419928
Anhalamine	0.331221353	0.381031126	0.352196387
Anhalonine	-0.259422855	0.605209677	0.041478853
Aniline	0.342965905	0.250090496	0.083691982

Aniracetam	-0.307687209	0.714088268	0.018904336
Anserine	0.247194584	0.472683886	0.318759213
Anthraniloyl-CoA	-1.593924436	0.019889936	0.357086101
Antibiotic G-418	0.190549742	0.363527032	0.125108063
Antibiotic JI-20A	0.167827365	0.612188951	0.665214349
Apramycin	-1.788368441	0.146019716	0.476596424
Arachidic acid	0.051012247	0.954485927	0.116088167
Arachidonate	-0.23117203	0.505346798	0.055080141
Arbutin	1.2099768	0.016521798	0.141968324
Arecoline	-0.664378465	0.503875532	0.146555422
Arprinocid	0.658524087	0.726733218	0.071130537
Aryl beta-D-glucoside	0.609733495	0.025528339	0.159102941
Aspartame	0.842376581	0.005180705	0.695608974
Aspartylglycosamine	0.638228323	0.270015597	0.109649895
Astemizole	-0.778400609	0.085589353	1.099601274
Astragalin	0.226958371	0.302838941	0.14309853
Asymmetric dimethylarginine	-1.823171826	0.015087489	0.166375
Atalaphylline	0.120504526	0.667064218	0.02621022
Atazanavir	-0.50247722	0.409589473	0.041050154
Atenolol	-0.029263847	0.9577716	0.031946662
Atorvastatin	-0.703432418	0.042267756	0.355790036
ATP	-0.629441084	0.116713175	0.345096701
Atraton	0.765112882	0.197209595	0.188710854
Atrazine	0.163933474	0.738649913	0.004520461
Aucubin	1.002447243	0.185040144	0.127369504
Aurachin B	-0.068233916	0.903734314	0.019730407
Avanafil	0.09035765	0.780133145	0.003894793
Avermectin A1b aglycone	-0.04443982	0.885089615	0.07535912
Avermectin B1a	0.980652852	0.005113423	0.267315666
Avermectin B1b aglycone	-1.020223816	0.074086637	0.284262102
Azacitidine	0.680089651	0.267224945	0.163632943
Azadirachtin A	-0.316088855	0.489589346	0.182255184
Azaperone	0.691121827	0.297312528	0.077920452
Azaserine	-0.131377701	0.799612664	0.015492312
Azatyrosine	0.193256746	0.651693525	0.029689294
Baclofen	0.360173158	0.102945399	0.250337228
BADGE	-0.182275854	0.689131977	0.055209134
Baicalein	-0.574547499	0.659903659	0.295946566
Beauvericin	-0.028335275	0.927735998	0.11631374
Benfuracarb	-0.595058726	0.303910328	0.116924026
Benthiavalicarb isopropyl	1.449766222	0.036758907	0.405520627
Benzaldehyde	0.278174962	0.210033251	0.293382575
Benzoylecgonine	0.617319712	0.037507673	0.498431221

Benzyl isothiocyanate	0.58188024	0.047911728	0.164152818
Bepridil	-0.041841699	0.860111295	0.349267653
Berberine	0.515730045	0.330917757	0.054668109
beta-Alanine	0.258791649	0.251499313	0.650157832
beta-Alanyl-L-arginine	-0.583712021	0.403504513	0.196603283
beta-Alanyl-L-lysine	0.302622792	0.510974705	0.048215063
Beta-Carboline	0.42979226	0.174037286	0.052751556
beta-Caryophyllene	-0.537651897	0.405868324	0.151163996
Beta-D-Glucose	0.587416267	0.029412763	0.472689392
Beta-Guanidinopropionic acid	0.061934362	0.807687659	0.04633577
Betaine	0.685845128	0.134801093	0.278726037
Beta-Leucine	-0.090902643	0.813724049	0.170437068
Beta-Tyrosine	-0.100372173	0.727476411	0.009804885
Bialaphos	-0.77804575	0.170221799	0.087224127
Bilirubin	0.124590905	0.659066449	0.035022298
Biliverdin	0.140049643	0.61500434	0.078573348
Bilobalide A	0.25759381	0.336067356	0.094819622
Biochanin A	0.197728305	0.438335822	0.002763869
Biocytin	-0.315274583	0.267439143	0.118965227
Bioresmethrin	-0.646659105	0.065559669	0.23802183
Biotin	0.254016792	0.663797165	0.383726862
Bis(beta-D-glucosyl) crocetin	0.310714105	0.551163255	0.054333887
Bis-gamma-glutamylcystine	0.063464261	0.914518056	0.021708809
Blasticidin S	-0.065935714	0.882611843	0.010374811
Bovinocidin	0.555073556	0.359993543	0.190411111
Bromacil	0.449862203	0.175153098	0.149233474
Bromocriptine	-0.281350634	0.463725935	0.106748862
Brompheniramine	0.094261187	0.855970802	0.010930099
Brucine	0.463766077	0.065798798	0.524036641
Bufalin	0.246605417	0.569281104	0.129112745
Buformin	0.317828004	0.432806531	0.069003047
Bufotenin	0.660007577	0.359537172	0.07367257
Buprofezin	-1.059372908	0.058494866	0.263179027
Bursehernin	-0.039826323	0.936134763	0.011194972
Butyryl-L-carnitine	0.847708763	0.183230405	0.752063534
Bz-Arg-OEt	0.058203492	0.887901598	0.04087033
Caffeic acid	-0.030475734	0.921992181	0.005412061
Caffeine	0.728245259	0.585927939	0.144176229
Calcitriol	0.268328741	0.3577667	0.10137261
Camptothecin	0.215125933	0.643697994	0.009051347
Cannabinol	0.305024375	0.576646819	0.11977072
Caproic acid	0.427276904	0.078339046	0.116346589
Carbamoyl phosphate	0.451936313	0.051139107	0.549124782

Carbetamide	0.430913045	0.275544429	0.222422335
Carbimazole	-0.83968797	0.239427036	0.081669123
Carboprost	0.211090214	0.797444064	0.00041347
Carbosulfan	0.098239899	0.67898899	0.044605734
Carfentanil	-0.163771542	0.472615192	0.102717918
Carisoprodol	-0.446013634	0.30383652	0.099736308
Carmustine	0.546188243	0.402782781	0.153920979
Catechin	0.387946858	0.448242367	0.103017178
Catechol	0.598029543	0.136115306	1.052316673
Cefdinir	-0.431706557	0.421024102	0.084876426
Celecoxib	-0.29609564	0.639147203	0.044792882
Celiprolol	-0.274686439	0.521102216	0.10714251
Cerulenin	0.351694279	0.429407889	0.120581051
Cetyltrimethylammonium bromide	0.857367397	0.087273422	0.167934914
Chavicol	0.61155792	0.094727028	0.656503203
Chicoric acid	0.350195646	0.547227359	0.104945561
Chitobiose	-0.196108943	0.497149619	0.055538637
Chloramphenicol 3-acetate	-0.476238099	0.172719056	0.306799589
Chlorfluazuron	-0.439407061	0.330814339	0.118662848
Chlorhexidine	-0.148272542	0.817391153	0.013980664
Chlormadinone acetate	0.492501184	0.494297089	0.135833344
Chloroxuron	1.495569482	0.001199255	0.301325553
Chlorpheniramine	-0.063797289	0.773293952	0.045835779
Chlorpyrifos-methyl	-0.340417812	0.559336357	0.083794407
Chlorsulfuron	-0.833574678	0.308102155	0.055045749
Chlorzoxazone	-0.122752394	0.598772015	0.045192024
Cholesterol sulfate	-2.570633686	0.084358758	0.346723579
Choline	0.538147393	0.03843711	2.617989168
Choline sulfate	0.191537447	0.365427341	0.115839468
Ciliatine	0.799421448	0.355337115	1.399982811
Cimetidine	-0.005876129	0.989584153	0.017913941
Cinnamaldehyde	1.070114204	0.129874157	0.458002703
Cinoxacin	0.314306348	0.495040085	0.033462962
cis-4,5-Dihydroxy-4,5-dihydropyrene	0.44124126	0.377140197	0.05925468
cis-4-Hydroxy-D-proline	-1.910687507	0.003755839	0.979353109
cis-4-Hydroxy-L-proline	0.450553044	0.177504385	0.455434222
cis-Aconitic acid	0.492647067	0.271581351	0.181756432
cis-N-Methyl-(S)-7,8,13,14-tetrahydroprotoberberine	0.863151904	0.029152865	0.229513421
Cis-zeatin	0.198065604	0.355787783	0.08659138
Citalopram	-2.333080868	0.161852392	0.795452047
Citicoline	1.480977749	0.006865995	0.16877419
Citreoviridin	-0.369712125	0.294167229	0.087847503
Citric acid	0.396325247	0.425751171	0.161612117

Citrinin	0.887884508	0.033562031	0.22193428
Citrulline	0.191361655	0.442969476	0.516944225
Clerodin	0.655187069	0.252161401	0.059733069
Clofentezine	0.155124281	0.5835246	0.017412921
Clofibrate	0.221475105	0.39241405	0.029645288
Clofibric acid	-0.394026764	0.230265155	0.111999698
Clopyralid	0.325280243	0.22975481	0.111198799
CMP	-0.045384446	0.970155135	0.12683553
Codeinone	0.216093289	0.642129933	0.042259608
Coelichelin	0.101377456	0.86298198	0.296738419
Coenzyme F420-1	0.74766602	0.044219207	0.296241534
Coniferin	-1.374997075	0.010183225	0.140375449
Cortexolone	0.399150482	0.299980811	0.285904393
Cortisol	0.461634855	0.258458483	0.047842338
Cortisone	0.244660033	0.539530161	0.024759073
Cortivazol	0.581072291	0.093287737	0.149251905
Corynebactin	0.238429682	0.680825973	0.072810479
Coumachlor	-1.235158232	0.085609434	0.203301505
Coumaphos	0.557146282	0.061848395	0.211428403
Coumarin	0.012707996	0.970872963	0.006020893
Coumestrol	-0.311120663	0.585391211	0.054393145
Cucurbitacin B	7.282654199	0.026272895	0.534474973
Curcumin monoglucoside	-0.839372831	0.099482695	0.204748744
Cyanazine	0.10764559	0.709635739	0.037551028
Cyanidin 3-galactoside	-0.995149671	0.019973589	0.244800052
Cyazofamid	-0.052504881	0.898310624	0.059319248
Cyclic AMP	1.146763309	0.018617437	0.23731854
Cyclic GMP	-0.132579144	0.615130915	0.107754507
Cyclohexylamine	-0.520181791	0.294819833	0.162654026
Cyclopentolate	0.099977944	0.877479784	0.033154758
Cyclopeptine	0.427555857	0.279781608	0.100313413
Cymarin	0.099923253	0.818798729	0.126709442
Cysteic acid	0.128043463	0.561557811	0.077843009
Cysteinylglycine	0.175816424	0.403388198	0.108432898
D-1-Piperideine-2-carboxylic acid	-0.386547296	0.568306981	0.06133751
Daidzein	0.37500496	0.150988713	0.195574813
Dapdiamide C	0.132886777	0.629154831	0.117786202
Dapsone	0.667089113	0.010928777	0.429880561
D-Cysteine	0.685098921	0.243281432	0.124516277
Deacetylvindoline	-0.192284363	0.412369174	0.577885414
Decanoyl-L-carnitine	0.95096905	0.310001617	0.816561401
Decarbamoylnovobiocin	-1.212549434	0.073059191	0.088317796
Deethylatrazine	0.573882635	0.202787908	0.085537288

Deferoxamine	0.17961891	0.735947915	0.301650263
Dehydroepiandrosterone	0.62492805	0.01522397	17.29722053
Dehypoxanthine futalosine	-0.333480577	0.324536531	0.907131186
Deisopropylatrazine	0.374456341	0.095571578	0.109772859
Deltaline	0.168408306	0.465911243	0.151696791
Delta-Tocopherol	0.536069617	0.059644174	0.10917608
Demethylated antipyrine	0.456239183	0.12372821	0.640916649
Demethyldecarbamoylnovobiocin	-0.279709834	0.554234338	0.197515487
Demethylisoalangiside	-0.457325936	0.442515534	0.043237527
Deoxyadenosine	0.89225171	0.342318608	0.078321894
Deoxycholic acid	0.310346974	0.151973769	0.573382924
Deoxycorticosterone acetate	-0.637756563	0.391128682	0.129113374
Deoxyguanosine	0.475917333	0.160158714	0.050098041
Deoxyinosine	0.022112956	0.965072539	0.005532068
Deoxyloganin	0.744562349	0.223853486	0.109496137
Deoxyuridine	0.192180921	0.368393978	0.315711695
Desaminotyrosine	0.629341673	0.160584551	0.157929699
Desmethylxanthohumol	1.30691157	0.328149815	0.060541288
Desmosterol	0.887700837	0.081508119	0.735226847
Dethiobiotin	0.584757438	0.061670335	0.105747675
Dextromethorphan	1.362184081	0.020842391	0.158408984
D-Fructose	0.150302232	0.542311486	0.065644553
D-Galactose	0.22959645	0.61906326	0.009499026
D-Glucose 1-phosphate	-0.639800157	0.252685068	0.146383054
D-Glucuronic acid	0.588059233	0.021265765	0.274356793
D-Glutamine	0.219935614	0.630650272	0.148489725
D-Glyceraldehyde 3-phosphate	0.011137521	0.98327374	0.024318179
Di(2-ethylhexyl) adipate	-0.681033661	0.29756596	0.146827495
Diacetoxyscirpenol	-0.84477914	0.15364629	0.180996306
Diadenosine triphosphate	-0.043006613	0.935359847	0.010066676
Diatrizoate	-0.736053583	0.183129878	0.328449323
Diazinon	1.497743148	0.006028804	0.286852103
Dibutyl phthalate	1.249175644	0.012476183	0.28889093
Diclofenac	0.099666701	0.720191236	0.03235223
Dicyclomine	0.34414342	0.124963188	1.051228564
Didecyldimethylammonium chloride	0.260322165	0.378264275	0.077734929
dienogest	-0.128079221	0.72025739	0.01877052
Diethyl phthalate	-0.082519073	0.870122823	0.445028661
Diethylphosphoric acid	-0.636538277	0.247402928	0.204326247
Diethylpropion	0.005084105	0.9840336	0.044414809
Digitoxin	0.705107603	0.012556435	0.271307443
Dihomo-gamma-linolenate	-0.434972749	0.441581904	0.075545986
Dihydroanhydropodorhizol	0.755369867	0.037582654	0.09541776

Dihydrocapsaicin	-0.569890795	0.22826643	0.15498591
Dihydroergotamine	-1.477636597	0.047925601	0.147987995
Dihydrogeranylgeranyl diphosphate	0.709122613	0.375596623	0.0539205
Dihydrostreptomycin 6-phosphate	0.47047366	0.193435912	0.25677971
Dihydrothymine	0.413986343	0.09629441	0.40525185
Dihydroxyacetone phosphate	-1.182813584	0.118219331	0.896524706
Diisodecyl phthalate	-0.254578834	0.557741994	0.092875705
Dimefuron	1.674500374	0.164085722	0.311238125
Dimethenamid	0.193528112	0.375887927	0.063746175
Dimethirimol	0.50900103	0.204681548	0.158477933
Dimethyl sulfone	0.748479547	0.053175399	1.254943918
Dimethyl trisulfide	-0.129556951	0.613921749	0.110193396
Dimethylglycine	0.821954988	0.01814428	1.290237521
dIMP	0.387039833	0.332455008	0.071544992
Diniconazole	0.237898447	0.513981018	0.061009286
Dioscin	1.310927223	0.001495694	0.497326667
Diosmetin	-0.136872177	0.708346176	0.052954835
Diphenhydramine	0.372098069	0.151877994	0.134696619
Diphenylamine	0.762800866	0.006111418	0.618549008
Dipyridamole	-0.857534151	0.042077332	0.809717402
Disopyramide	-0.516887327	0.617659202	0.037455919
DL-Glutamate	-0.034520132	0.950492836	0.010639931
D-Lysopine	2.94265735	0.001743014	0.209504509
D-Lyxose	-0.99112605	0.211960398	0.589552844
D-Malic acid	0.501813083	0.209713405	0.208494444
D-Mannose	0.4243278	0.070433663	0.227167891
Dobutamine	1.979804855	0.303640803	0.262195057
Docosahexaenoic acid	-0.558564204	0.255131044	0.542927578
D-Octopine	1.592814253	0.000159023	0.289143683
Dodecanedioic acid	0.321897777	0.128702389	0.242208265
Dodecanoic acid	-1.063763226	0.021372166	1.791304933
Dolichotheline	0.397234974	0.325978874	0.117265182
D-Ornithine	0.054369908	0.800729487	0.098613698
D-Ornithine hydrochloride	0.1576589	0.455322659	0.14389003
D-Phenyllactic acid	0.505601616	0.074045784	0.663358021
D-Pinitol	-1.11362142	0.019659762	0.581885397
D-Ribose 5-phosphate	0.274184609	0.339196357	0.148976255
D-synephrine	0.399102425	0.087190897	0.508554523
D-Tartaric acid	0.29637883	0.196336622	0.423028286
D-Xylitol	1.591253606	0.000989008	0.667473542
Dyphylline	0.986577779	0.091782771	0.132272581
Ecdysone	-0.346169567	0.535898331	0.163243548
Ecgonine	-0.093986612	0.817070979	0.026197252

Ecgonine methyl ester	0.570505352	0.113377251	0.133080676
Ectoine	-0.005552064	0.987381393	0.010180483
Edifenphos	-1.234777244	0.152603218	0.06331341
Edrophonium	0.067416988	0.773752434	0.011275387
Eicosapentaenoic Acid	-0.218069441	0.69102898	0.040854201
Ellagic acid	0.142389046	0.628876	0.043104605
Emetine	-0.150763968	0.712138668	0.058165465
Enalapril	-1.205570934	0.05506815	0.375173679
Enalaprilat	-0.111974913	0.770001226	0.031899376
Enilconazole	-0.036778291	0.890426825	0.028484596
Enol-phenylpyruvate	0.489156315	0.096976081	0.803172675
Enoxacin	1.093067342	0.076270274	0.089413275
Epiandrosterone	0.227344146	0.44874236	0.039092852
Epinephrine	-1.920393502	0.014443372	7.292390003
epsilon-(gamma-L-Glutamyl)-L-lysine	0.308398121	0.541584185	0.123394169
Epsilon-caprolactam	-0.131737299	0.679247791	0.197895949
Equol	0.55961	0.090407897	0.605715053
Ergothioneine	-0.970044772	0.206205164	0.134688421
Erucic acid	-0.035566186	0.950980509	0.317488755
Erythritol	0.248340783	0.679519211	0.249861646
Esculetin	0.062060868	0.818824104	0.093421853
Esmolol	-0.600084156	0.373420901	0.134295147
Estrone	0.239369105	0.661418717	0.117304272
Ethoprophos	0.312911456	0.20187678	0.109303924
Ethyl icosapentate	1.41189417	0.040406106	0.125189062
Ethylene glycol tetraacetic acid (EGTA)	-1.078747163	0.178165986	0.100771148
Ethynodiol Diacetate	-0.59655423	0.085506169	0.244502606
Etoxazole	0.287406057	0.161665752	0.270092899
Eugenol	-0.013103876	0.970210352	0.033899707
Exemestane	0.674172555	0.451092604	0.057053388
FAPy-adenine	0.146063895	0.760297135	0.351854514
Farnesoic acid	0.093858984	0.683959713	0.024828891
Fenfluramine	0.847909543	0.129385169	0.593172068
Fenpyroximate	-0.595112465	0.662651802	0.060211688
Fenthion	0.011287811	0.972405656	0.022725904
Flavonol 3-O-(6-O-malonyl-beta-D-glucoside)	1.067573618	0.005853152	0.245268043
Flavonol 3-O-[alpha-L-rhamnosyl-(1->6)-beta-D-glucoside]	0.915923366	0.16042835	0.104538199
Flavonol 3-O-beta-D-glucosyl-(1->2)-beta-D-glucosyl-(1->2)-beta-D-glucos	0.317135464	0.605490017	0.070502343
Florasulam	0.110637858	0.674818279	0.026648052
Flumazenil	1.068942306	0.298857628	0.086785173
Flunisolide	-0.343253768	0.665641064	0.094639031
Fluorene	1.221934033	0.030796672	0.251402979
Fluperlapine	0.333555893	0.375114924	0.245432708

Fluroxypyr	0.061448168	0.835106626	0.007469638
Flutamide	0.251583104	0.362157876	0.23859545
Fluvastatin	-0.06629964	0.760909035	0.142921641
Fluvoxamine	-0.173674476	0.710959085	0.038527923
FMLP	-1.669831352	0.293496294	0.298263625
Folic acid	1.215055013	0.013705697	0.167656622
Folinic acid	-3.593004448	0.199362183	1.07143948
Formononetin	-0.391702005	0.446677323	0.413480809
Formoterol	-0.115909724	0.68500532	0.108170265
Formylanthranilic acid	0.436604367	0.16761025	0.105907756
Fosinopril	0.615921631	0.018145971	0.348854472
Fraxetin	1.020081321	0.022948314	0.429750985
Fructose 6-phosphate	0.213170426	0.376187159	0.165513682
Fucose 1-phosphate	0.64595973	0.037850958	0.189968091
Fumaric acid	-0.802213931	0.06202507	0.781362951
Fumitremorgin A	0.16878599	0.47105869	0.07674883
Furosemide	-1.787513395	0.145597435	0.270551199
Gabapentin	0.110035936	0.623507664	0.061778547
Galactaric acid	-0.161156375	0.459855071	0.087959462
Galantamine	1.00351329	0.010638492	0.890754593
Gallic acid	0.018463209	0.963495809	0.010861789
Gallopamil	-0.705074272	0.161609305	0.089044229
Gambogic acid	-0.640833091	0.217014275	0.285230954
gamma-Aminobutyric acid	0.53967374	0.017790428	0.263451038
gamma-Glutamylalanine	1.18296878	0.000702946	0.997598432
gamma-Glutamyl-beta-aminopropiononitrile	0.010745025	0.98212902	0.046240647
gamma-Glutamyl-beta-cyanoalanine	-0.891845932	0.050951202	0.188202624
gamma-Glutamylcysteine	0.196481393	0.460711267	0.095283233
Geissospermine	0.891613001	0.003310321	0.393446096
Geldanamycin	0.902264339	0.093062336	0.142090708
Gelsemicine	0.56622757	0.105188735	0.134402847
Gemcitabine	-0.101193846	0.908487004	0.059583145
Genipin	1.797028892	0.006642945	0.283766022
Genistein	0.03204004	0.881867365	0.000217629
Genistin	-0.086496977	0.795866163	0.208316964
Gentamicin A2	1.200188948	0.00908838	0.262468223
Gentamicin C1a	-0.247391744	0.490021974	0.124110128
Gentamicin X2	-0.720449138	0.300849492	0.218714718
Geranyl acetate	0.614966667	0.209369243	0.087787076
Geranylacetone	0.811369362	0.008969816	0.374404513
Geranylgeranyl-PP	0.703745624	0.034552333	0.159921619
GF 109203X	-0.217297026	0.775028177	0.07564482
Gibberellin A7	-1.457394674	0.123000849	0.087753048

Glucaric acid	0.528736167	0.022758302	0.435398917
Glutathione	0.057338895	0.91353554	0.002157688
Glyburide	-0.543753329	0.140311893	0.148709883
Glyceric acid	0.142955398	0.606575914	0.119741191
Glycerol	0.145512832	0.579173015	0.101377521
Glycerophosphocholine	-0.137607316	0.688082545	0.517025204
Glycerophosphorylcholine	0.087134329	0.860935791	0.051237085
Glycitin	0.224654397	0.432722379	0.089850893
Glycyl-leucine	-0.020432506	0.961694376	0.043800477
Glyphosate	0.140485837	0.551579935	0.032806552
GMP	0.400952559	0.086913254	2.241689534
Granisetron	0.404968371	0.334827911	0.170502292
Guanabenz	-0.890927079	0.287485293	0.05693528
Guanethidine	0.043212687	0.871415378	0.014143633
Guanfacine	0.132452109	0.609201535	0.085610757
Guanidoacetic acid	-3.26726478	0.297568503	0.812778479
Guanosine	-0.812616986	0.193579527	0.302159752
GW 7647	0.124122614	0.780833062	0.02096939
Haloperidol	-0.105117181	0.907734327	0.000349049
Harmaline	0.38398954	0.152002275	0.668899319
Harpagoside	0.014171556	0.979522774	0.01231671
HC-toxin	-0.668806082	0.34074937	0.990808786
Heme	-0.377382472	0.641766481	0.282829336
Heptanoic acid	0.603370225	0.085557795	0.672127991
Heptenophos	-0.0938941	0.664328919	0.087046547
Herniarin	0.317370543	0.153360288	0.120106249
Hesperetin	0.620943897	0.365138083	0.241615448
Hexylamine	0.187057926	0.516915623	0.035221022
Histamine	-0.014670408	0.987745721	0.180237521
Homocitric acid	-0.711196892	0.101533125	0.312792284
Homocitrulline	0.647392618	0.158194836	0.114521829
Homogentisic acid	0.435287699	0.532719671	0.233915611
Homovanillic acid	0.233559262	0.595599506	0.932149168
Honokiol	-0.63144385	0.320548655	0.210601904
HT-2 Toxin	1.810546468	0.022983812	0.268311357
Hydrochlorothiazide	0.104658597	0.680886021	0.013912005
Hydrocortisone aceponate	-1.778301151	0.039666461	0.548053263
Hydrogen phosphate	-0.702447947	0.183767862	0.184474186
Hydroxychloroquine	-0.268354644	0.246163106	0.204944239
Hydroxyzine	-0.201257057	0.569361453	0.061952418
Hygromycin B	0.611932937	0.020857667	0.119492483
Hypaconitine	-0.37458572	0.280829195	0.105283906
Icariin	1.112882234	0.003617278	0.135797712

Imazapyr	-0.560727056	0.435449115	0.087687635
Imidacloprid	0.124480785	0.794824026	0.061497875
Imidazol-5-yl-pyruvate	0.339441831	0.146855191	0.304376664
Imidazolelactic acid	-0.136601939	0.670931832	0.077848054
Iminoarginine	-0.456832729	0.502976488	0.298171773
Imipramine	0.401130451	0.109195168	0.230674772
Immunomycin	0.395547941	0.16548413	0.101430652
IMP	0.264775689	0.622241367	0.01854903
Indinavir	0.563384451	0.097349982	0.348382605
Indole	0.367885761	0.104234539	0.342855857
Indoleacetaldehyde	0.54246564	0.039146009	0.690670279
Indolebutyric acid	0.232948309	0.696839275	0.044410121
Indoleglycerol phosphate	0.393991423	0.295420377	0.383747
Indolelactic acid	0.37248338	0.348475561	0.203653438
Indolepyruvate	0.349784919	0.225460986	0.625825447
Indoxacarb	-0.92960238	0.082293747	0.307175441
Ipratropium	0.040675196	0.94151652	0.033476992
Iprodione	-0.049329264	0.846989792	0.044519813
Irbesartan	-0.60357325	0.377591932	0.120211275
Isoalangiside	-0.612558903	0.386112417	0.091151653
Isochavicol	0.860162091	0.002386388	0.61658799
Isocitric acid	-1.259245625	0.249763219	0.331698274
Isoetharine	0.144150409	0.517455768	0.048775445
Isoferulic acid	-0.364956577	0.349324892	0.148227151
Isoguvacine	-0.935528035	0.593153236	0.99234552
Isolithocholic acid	0.58176761	0.043887792	1.256000458
Isoniazid	0.573963282	0.072214238	0.245668053
Isoniazid alpha-ketoglutaric acid	-0.551013379	0.445322984	0.060280473
Isopentenyl adenosine	1.029699473	0.031496202	0.230775752
Isopimpinellin	0.355001915	0.546352428	0.047999054
Isoprothiolane	-0.186040616	0.8518499	0.464771497
Isouron	-0.342058122	0.159908229	0.385162365
Juglone	0.319004586	0.345618603	0.19061127
Kanamycin B	-0.392333734	0.69151331	0.177867267
Ketamine	0.588647176	0.056818152	0.205840225
Ketoleucine	0.147288969	0.546244992	0.140357174
Ketorolac	0.930140175	0.037582641	0.098894409
Khellin	0.372493806	0.11760374	0.115115402
Kojic acid	-0.236294256	0.405258455	0.121176538
Kynurenic acid	0.40445348	0.100831017	0.360306276
Kyotorphin	0.372284508	0.361484134	0.187669882
L(-)-Carnitine	0.09886307	0.732738432	0.494912287
L-2,4-diaminobutyric acid	0.344683975	0.138108804	0.505599687

L-2-Hydroxyglutaric acid	0.61446015	0.035362151	0.847528097
L-3-Cyanoalanine	-0.486929209	0.135087385	0.331919602
L-3-Hydroxykynurenine	-0.664366714	0.301301553	0.145195354
L-4-Hydroxyphenylglycine	-0.129601458	0.709457086	0.908462795
L-Alanine	0.316687734	0.433933207	0.127343629
L-Alanyl-gamma-D-glutamyl-L-lysine	0.733517351	0.009450909	0.205732826
L-Allothreonine	1.268172629	0.004207558	1.613776189
Lamivudine	0.063557343	0.843324442	0.051173314
Lamotrigine	-0.91386677	0.279125182	0.05470016
Lanosterin	0.907243468	0.012875957	0.326418389
Lappaconitine	-0.214728133	0.469874578	0.530274526
L-Arabinose	0.547095593	0.074358394	0.545162741
L-Arabitol	0.669433122	0.022055025	0.459211852
L-Arogenate	0.371594646	0.142081241	0.404845435
L-Asparagine	0.37448617	0.119666813	0.30794923
L-Aspartate-semialdehyde	0.216772265	0.516209152	0.120041357
Latrunculin B	-0.464285953	0.273304389	0.218551897
Lauryltrimethylaminium bromide	0.03867145	0.909399317	0.015731297
L-beta-Phenylalanine	0.839070127	0.269082326	0.327177955
L-Carnitine	0.029477042	0.907901801	0.045772504
L-Citramalyl-CoA	-0.638538804	0.172627421	0.085338376
L-Cystathionine	0.392423021	0.160272477	0.218381095
L-Cystine	1.183988334	0.01221671	0.384294385
Lenacil	0.309686073	0.627377595	0.036313852
Lenticin	-0.879467507	0.076136723	0.903814626
Letrozole	-0.85209959	0.102609621	0.267104595
Leukotriene E4	-0.823316854	0.260601808	0.231743577
Levamisole hydrochloride	0.398665192	0.090245819	0.626224059
Levonordefrin	1.43452426	0.021990232	1.052608265
Levonorgestrel	1.014871033	0.018530246	0.171254022
Levorphanol	-0.020643335	0.964969928	0.005724482
L-Fucose	0.610602242	0.130838391	0.451830674
L-Glutamic acid 5-phosphate	-0.151207793	0.697257977	0.045645974
L-Glutamic gamma-semialdehyde	0.659057094	0.010543682	0.705623679
L-Gulonolactone	3.247507247	0.016569061	2.781949414
L-Histidine	-0.003457936	0.992192289	0.051506854
L-Histidine trimethylbetaine	-0.62902999	0.28776178	0.194945368
L-Iditol	0.655832847	0.03744108	2.169994493
Linatine	0.744696015	0.1608511	0.154041428
Lipoamide	-0.594675438	0.256492885	0.492968841
Lipoxin B4	0.430361841	0.151917003	0.279214558
L-Isoleucine	0.265534945	0.405713155	3.851876309
Lithocholic acid	0.320081619	0.153893283	0.373644767

Lithocholyltaurine	0.354641623	0.598403387	0.027522281
Lividamine	-0.598772693	0.286581533	0.224131776
L-Kynurenine	-1.823916088	0.252535948	0.471937042
L-Lactic acid	0.679457253	0.127734426	0.279459795
L-Lysine	0.327535348	0.24907697	1.442495083
L-Methionine	0.46426868	0.241508348	2.041065005
L-Norleucine	0.235928207	0.454936091	0.224830387
L-Norvaline	-0.550793667	0.031130714	0.344407875
L-Octanoylcarnitine	1.031627946	0.263438251	0.650992196
Lomefloxacin	0.387811602	0.391420799	0.108286439
Loratadine	-3.173836422	0.076789055	0.483902432
Lorazepam	-0.812899726	0.151765483	0.135828924
Lotaustralin	0.366716445	0.405302278	0.311375518
L-Phosphoarginine	0.35306509	0.171663401	0.073562256
L-Prolinamide	0.142644742	0.549580941	0.089570405
L-Proline	0.14658726	0.615381253	0.038574473
L-Serine O-sulfate	0.058149972	0.834360923	0.007727764
L-Targinine	0.274191131	0.387724923	0.180117407
L-Theanine	0.624784483	0.142086819	0.185812263
L-Threonine	0.374125653	0.097256081	0.994352766
L-Tryptophan	0.356140432	0.140278123	3.819897503
L-Tyrosine	0.497658499	0.085633822	5.427361559
Lubiprostone	-0.068095609	0.869034555	0.02035537
Lumichrome	0.239280774	0.247123852	0.160589822
Lunarine	-0.767339381	0.303976725	0.518860042
Lupinine	0.99824754	0.013705555	0.229045823
L-Valine	-0.005232496	0.985304809	0.295942088
Magnesium protoporphyrin	0.380478738	0.247976876	0.055431886
Malaoxon	-0.672934055	0.310277826	0.090279521
Maleimide	0.492401825	0.16071494	0.610149627
Maltotetraose	-1.327172843	0.321385868	0.217483755
Mandelic acid	0.22650623	0.489806045	0.261987992
Manidipine	-0.775665604	0.18589069	0.218059539
Maslinic acid	0.694498757	0.133637519	0.168027149
m-Cresol	0.31520374	0.193034808	0.733783958
Medazepam	-0.434872479	0.696107998	0.21691146
Melphalan	0.327369301	0.198963004	0.206247283
Meperidine	1.228797447	0.017588806	0.310351126
Mepyramine	0.328586781	0.602748072	0.115097738
Mercaptopurine	1.128986683	0.091221719	0.193230908
Mesaconitine	0.4314591	0.176028927	0.0659375
Mesobilirubinogen	0.183370074	0.772069258	0.017483176
Mesoridazine	0.28092791	0.471803694	0.050382967

Metalaxyl	0.345447235	0.288315384	0.106867741
Methabenzthiazuron	0.260828306	0.283648624	0.148013411
Methazolamide	0.409120279	0.329924402	0.108134275
Methidathion	0.904282382	0.006579771	0.30232482
Methionine sulfoximine	-1.039052048	0.004054484	1.250776168
Methomyl	0.014181779	0.965021427	0.000708131
Methoprene	0.112636795	0.737291679	0.040455262
Methotrexate	-0.338033377	0.31459725	0.195847834
Methotrimeprazine	0.497981024	0.533610019	0.049651148
Methoxamine	-0.431409652	0.512556768	0.031334579
Methoxyfenozide	0.332575909	0.422552404	0.034525674
Methyl (indol-3-yl)acetate	0.880655522	0.645004585	0.188564178
Methyl aklanonate	1.715411751	0.105499521	0.09688753
Methyl isobutyl ketone	-0.127705023	0.697530585	0.017788775
Methyleugenol	0.266338963	0.212972119	0.236219531
Methylisoeugenol	1.093766353	0.003632387	0.281183049
Methylmalonic acid	-0.463966597	0.584763901	0.111940031
Methylselenopyruvate	-1.436932179	0.449256957	0.097779796
Metobromuron	-0.622326456	0.065757359	0.412845392
Metoclopramide	0.240846343	0.251245036	0.140787498
Metoprolol	0.079616301	0.872072556	0.049709629
Metosulam	-0.055552707	0.886520761	0.018297528
Metoxuron	0.501310167	0.141068836	0.059601513
Metyrapone	0.512831594	0.251479685	0.325657597
Mevalonic acid	-0.236757711	0.736842242	0.182019699
Mevalonic acid-5P	0.248050684	0.30571907	0.07508037
Mevinphos	0.279909167	0.214720373	0.105711768
Mexiletine	0.544188559	0.237716389	0.136142337
Miconazole	-0.611142052	0.197796486	0.164910838
Midodrine	2.121282984	0.0129823	0.170429026
Mifepristone	-0.316102177	0.359286796	0.100734674
Miglitol	0.202435647	0.482176993	0.490106745
Mirtazapine	-0.446692723	0.41178116	0.151791902
Mitomycin	0.666014219	0.145713226	0.103996302
Mitragynine	-0.121199851	0.635491199	0.074049228
Molindone	1.848627818	0.007985515	0.155401288
Monensin	-0.529481905	0.444836889	0.190834699
Monomethyl sulfate	-1.622827076	0.003916584	0.807046401
Monuron	0.643751517	0.332984665	0.25601249
Mukurozidiol	0.043091478	0.942247323	0.034760997
Muramic acid	0.122934947	0.752304857	0.030441873
Myclobutanil	0.052693632	0.935330699	0.165549791
Mycophenolate	0.288121916	0.509558836	0.056612276

Myo-inositol hexakisphosphate	-0.005948085	0.983068455	0.078783438
Myricetin	0.625631075	0.058161171	0.086986021
Myristic acid	-0.126809411	0.634822239	0.077281589
N(6)-Methyllysine	-0.014210997	0.975687982	0.013029665
N(omega)-Nitro-L-arginine methyl ester	-0.709503882	0.127416733	0.195942973
N,N-Diethylglycine	0.759584734	0.456235946	0.873002499
N,N-Diethyl-m-toluamide	-1.986280684	0.015285584	0.83717624
N,N-Dimethylsphingosine	0.292164069	0.412853796	0.478429874
N1,N12-Diacetylspermine	0.719707777	0.107071447	0.130334108
N1,N5,N10-Tricoumaroyl spermidine	0.30222633	0.506533822	0.120751735
N1-Acetylspermidine	0.75558943	0.035349636	0.363761975
N2'-Acetylgentamicin C1a	-1.518773086	0.199744596	0.116984337
N2-Malonyl-D-tryptophan	0.403525821	0.319535145	0.119355834
N2-Succinyl-L-ornithine	1.461665823	0.002071263	0.630446374
N4-Acetylsulfamethoxazole	-0.336737518	0.761375648	0.094317547
N6-(Delta2-Isopentenyl)-adenine	0.587636477	0.165778832	0.070959653
N6-Acetyl-L-lysine	-0.030021568	0.884400755	0.039636799
Nabumetone	-0.000137255	0.999746155	0.014359129
N-Acetyl-a-neuraminic acid	0.741171734	0.323197658	0.209930795
N-Acetylarylamine	0.640603319	0.02792792	0.441138633
N-Acetyl-beta-glucosaminylamine	0.021642438	0.938881228	0.047838571
N-Acetyldemethylphosphinothricin	-0.111403581	0.706817321	0.110663727
N-Acetyl-D-tryptophan	0.370264867	0.571127783	0.143527397
N-Acetyl-L-aspartic acid	0.599920175	0.058924163	0.120118317
N-Acetylleucine	-0.051573922	0.831891963	0.039277436
N-Acetyl-L-glutamate 5-semialdehyde	-0.423285366	0.307534607	0.117534978
N-Acetylornithine	0.070981784	0.839511375	0.036528833
N-Acetylputrescine	-0.986284416	0.010555188	0.259517754
N-Acetylserotonin	0.451800219	0.232035552	0.101964324
NAD+	0.133360365	0.700393208	0.024587896
NADP	0.601244081	0.153559235	0.106401169
NADPH	-0.223495659	0.641974435	0.069840226
Naftifine	0.273822515	0.493183386	0.045257813
Nalbuphine	-0.011178052	0.963390427	0.098633094
Nalidixic acid	0.477949845	0.274019141	0.306205688
Nalorphine	-1.776705857	0.028726491	0.720936084
N-Alpha-acetyllysine	-0.465868734	0.350879951	0.299094377
Nalpha-Methylhistidine	0.595984839	0.185340291	0.067789847
Naltrindole	-0.475129122	0.268944574	0.070464878
N-Amidino-L-glutamate	0.394058119	0.396426256	0.074966022
Nandrolone	0.92728698	0.009164991	0.960488648
Naproanilide	-0.345181298	0.429970288	0.084264148
Napropamide	0.082370473	0.907927425	0.013842641

N-Benzoylanthranilic acid	0.31080996	0.128705174	0.242587539
N-Butyryl-L-homoserine lactone	2.163566944	0.025467508	0.371086453
N-Demethylansamitocin P-3	1.220855191	0.002371826	1.538541992
N-Demethyl-desepoxymaytansinol	0.258472246	0.639870888	0.008097407
N-Desmethylcitalopram	-0.378903607	0.491473173	0.043461025
Neburon	0.242372923	0.414984073	0.120912298
Nelfinavir	-0.112298568	0.775769752	0.021069197
Neocnidilide	-1.077695307	0.080489729	0.320718751
Neostigmine	0.547297058	0.258471182	0.151013897
Neriifolin	-0.557977098	0.476632148	0.039571504
Nevirapine	0.797246715	0.145794533	0.128241796
N-Formyl-L-glutamic acid	0.42155157	0.299924911	0.312212111
N-Formyl-L-methionine	0.162980729	0.733032474	0.094304014
Niacinamide	0.33513018	0.241478693	0.375762657
Niaprazine	-0.070121919	0.823157509	0.028741909
Nicotianamine	0.190561524	0.679845818	0.02737354
Nicotinamide riboside	0.240327117	0.460626181	0.117253266
Nicotinamide ribotide	0.212709562	0.665015633	0.024468231
Nicotine	0.368652595	0.290247598	0.151957924
Nicotinic acid mononucleotide	-0.235084018	0.674419983	0.09122004
Nicotinuric acid	0.542851513	0.245364337	0.165087469
Nitrazepam	0.50357288	0.236633311	0.098442216
Nizatidine	0.048554096	0.883375519	0.018286077
N-Methyl-(R,S)-tetrahydrobenzylisoquinoline	0.107518902	0.69915527	0.03154149
N-Methyl-D-aspartic acid	0.680081863	0.005267962	2.294743065
N-Methylhydantoin	0.376700473	0.063211851	0.228072501
N-Nitrosodi-n-butylamine	0.811096089	0.009533831	0.306906363
N-Nitroso-pyrrolidine	-1.686927806	0.372212742	0.678415452
Nobiletin	-0.161647336	0.597737094	0.04699611
Nopaline	0.835449465	0.013529032	0.28500235
Norbelladine	-0.302420128	0.571624671	0.10075773
Norcraugsodine	-0.029821731	0.953534774	0.0723894
Nordazepam	0.583631943	0.043358454	0.312470142
Norepinephrine	0.116472256	0.631252109	0.246627098
Norethindrone	-0.31449646	0.569052792	0.087294447
Norfloxacin	0.18373472	0.746523918	0.15918992
Normetanephrine	1.094538451	0.097318847	0.476868331
Norsanguinarine	0.587503951	0.239266376	0.238976396
Novobiocin	0.570667263	0.168365092	0.072961242
N-Succinyl-2-amino-6-ketopimelate	0.498758351	0.348751381	0.238739078
Nudicauline	0.460546735	0.105823144	0.327994771
O-Acetylcarnitine	2.327623172	0.173994244	1.442493572
Octanal	0.111987601	0.686051491	0.005680766

O-Demethyl-N-demethyl-staurosporine	-0.259703395	0.454841566	0.205637749
O-Demethylpuromycin	0.461751446	0.034011857	0.210889494
Ofloxacin	1.191431099	0.239026924	0.107026042
Okadaic acid	0.435511729	0.11981374	0.214705065
Oleic acid	0.23975321	0.246366987	2.19370779
Omethoate	0.576503407	0.385851862	0.169418241
Ononin	0.789190671	0.004902783	0.117210498
O-Phosphoethanolamine	0.362928149	0.092846372	0.476787975
O-Phosphothreonine	0.539034066	0.10514621	0.235227203
Ophthalmate	-0.084998268	0.786837824	0.027728841
Orciprenaline	0.414342388	0.069059901	0.39271443
Osthol	1.039372076	0.097848896	0.225850089
O-Succinyl-L-homoserine	0.991944983	0.099145543	0.174158234
Oxalacetic acid	0.25513611	0.239858237	0.282904472
Oxaloacetate 4-methyl ester	0.343345532	0.201823625	0.155497271
Oxaloglutarate	0.241122818	0.278745565	0.196718125
Oxalosuccinic acid	-0.293146276	0.393304542	0.265744117
Oxazepam	0.037826591	0.897254124	0.00137818
Oxethazaine	-0.501984937	0.477906638	0.092099345
Oxidized glutathione	1.44333973	0.162238955	0.114875199
Oxoglutaric acid	0.766246381	0.035182423	0.720841967
Oxolinic acid	0.245425884	0.250840826	0.134360549
Oxybutynin	0.466926858	0.375459841	0.142929122
Oxycarboxin	-0.017639504	0.974705618	0.028117966
Oxyphenbutazone	1.101238165	0.007466748	0.372176647
Oxytetracycline	-0.432083877	0.466571897	0.06881953
Paclobutrazol	0.063206252	0.821438739	0.016646644
Palmitoyl-L-carnitine	0.531487243	0.307826345	0.669104528
p-Aminobenzoic acid	0.016632048	0.949483906	0.069510524
Pantothenic acid	0.599517115	0.180877139	0.24787784
pantothenic acid	1.24193209	0.173095447	0.130075821
Pantothenol	1.51556273	0.000257931	0.283190209
para-Aminoazobenzene	0.388526802	0.370061717	0.103658072
Paspalicine	-0.303340559	0.410909478	0.109438937
Penicillic acid	0.395423469	0.113707225	0.329726238
Penitrem A	-0.723927612	0.090098442	0.320192662
Pentamidine	0.03552787	0.91860235	0.020238924
Pentazocine	0.022619894	0.959267579	0.035332785
Pentoxifylline	0.274723647	0.555672636	1.380888068
Perakine	-1.346719771	0.037829639	0.352210815
Pergolide	0.944270008	0.080761281	0.158725348
Phenmetrazine	0.874925545	0.368820485	0.061739135
Phenol	0.315849162	0.130667161	0.146769357

Phenolphthalein	1.541087249	0.019893179	0.277245939
Phenothiazine	0.243086501	0.629352208	0.066283705
Phenylacetaldehyde	0.52845713	0.062280404	0.463655883
Phenylacetic acid	-0.057742123	0.86284797	0.095538826
Phenylacetylglutamine	-0.05780661	0.90231112	0.107024638
Phenylacetylglycine	0.616871313	0.232633607	0.099759047
Phenylbutazone	0.05161155	0.841232042	0.000831578
Phenylethylamine	0.351175072	0.134749091	1.267402775
Phenylpyruvic acid	0.168047301	0.598204585	0.031247091
Phosphocreatine	1.193241091	0.004456362	0.169814547
Phosphoenolpyruvic acid	0.022474882	0.91641819	0.001421864
Phosphoglycolic acid	0.312463987	0.588627497	3.233221647
Phosphohydroxypyruvic acid	0.379080619	0.44632255	0.127597873
Phosphorylcholine	-0.171957856	0.630506419	0.150029456
Phylloquinol	0.179732064	0.373512143	0.080258434
Phytyl diphosphate	0.133442118	0.746607123	0.010678462
Piceatannol	0.739609268	0.102109495	0.105441056
Pimelic acid	-0.337470097	0.294568225	0.192373687
Pimozide	0.720955208	0.07829137	0.465745254
Pimpinellin	0.386360261	0.153949283	0.148430611
Pindolol	0.35729935	0.54304095	0.08242978
Pioglitazone	0.207926796	0.52996889	0.122179009
Pipecolic acid	0.410608781	0.357849336	0.457141056
Piperine	0.8554418	0.332229304	0.77758546
Piperonal	-0.614951173	0.261856262	0.947969875
Piplartine	2.844905539	0.072177581	0.130919334
Pirbuterol	0.44017445	0.189878374	0.123571289
Pirimiphos-ethyl	0.71770135	0.402486071	0.063873988
Plastoquinol-9	1.049931246	0.064778294	0.136709037
p-Octopamine	-0.346460152	0.29325531	0.619683076
Polygodial	0.035798659	0.951815552	0.024642876
Pomiferin	1.339117984	0.095374619	0.269002892
Porphobilinogen	0.195175362	0.481042973	0.14884098
Practolol	0.761895026	0.02985807	0.72440517
Pramoxine	-0.249206088	0.787642121	0.001772112
Prazepam	0.486278056	0.283670572	0.191793706
Precocene II	-0.309771497	0.558459389	0.155122602
Prednisone	-0.386078943	0.535517722	0.041055935
Prephenate	0.295563542	0.289419191	0.505115015
Procaine	1.316849682	0.27988934	0.305598266
Procarbazine hydrochloride	1.519857256	0.322258218	0.312510659
Prochlorperazine	0.511824323	0.170561572	0.125333988
Procymidone	0.533913802	0.029161015	0.254172508

Prodiamine	0.000583674	0.998651399	0.010174255
Profenofos	0.054528423	0.930700746	0.018162713
Progesterone	2.055442823	0.097115275	0.439422933
Proguanil	-0.331527503	0.53739922	0.058524874
Promazine	0.628301191	0.13041822	0.149254023
Promethazine	0.779923391	0.306708593	0.15804329
Propanil	0.304096444	0.564702241	0.255671206
Propanoyl phosphate	0.332628405	0.140715651	0.562546088
Propazine	-0.150673288	0.738193989	0.040961499
Propinol adenylate	0.236133316	0.296669832	0.041226594
Propionylcarnitine	1.270391363	0.249697627	0.99756451
Propoxur	0.414008292	0.123206204	0.119805401
Propoxyphene	0.677219996	0.534197108	0.135215608
Propylthiouracil	0.183889858	0.540952211	0.067016667
Propyphenazon	0.71627932	0.388478989	0.114569631
Prostaglandin E1	-0.54231341	0.178710694	0.291285435
Prostaglandin F2b	1.086971596	0.065239367	0.20389839
Prostaglandin-c2	-0.241819928	0.644821094	0.113136291
Protopine	-0.344180035	0.762719675	0.010858251
Protoporphyrin IX	0.42239739	0.060655204	0.291119133
Protoporphyrinogen IX	0.296888789	0.485939813	0.785776587
Protoveratrine A	0.571057276	0.052589675	0.153930218
Protoveratrine B	0.782840029	0.058740113	0.405785977
Pseudoephedrine	-0.878269927	0.048654795	0.194519799
psi-Pelletierine	0.338912512	0.263471325	0.114593389
Psoralen	0.154218536	0.663163087	0.022888362
Psoralidin	0.144169442	0.768366933	0.02098892
Pterin	-0.330169901	0.470452009	0.11716005
Pumiloside	-0.881762962	0.507361403	0.034975775
Purine	0.357778325	0.381009973	0.107244129
Puromycin	-0.689592069	0.381963809	0.088000405
Pymetrozine	1.106301589	0.030751804	0.149996021
Pyrantel	1.338666868	0.000428079	0.317595003
Pyrazophos	0.280145036	0.218142492	0.046093489
Pyridostigmine	-0.235955637	0.647024212	0.078755113
Pyridoxal	1.219788191	0.024709534	0.139165118
Pyridoxal 5'-phosphate	1.249143725	0.002316574	0.591606547
Pyridoxamine	0.209514145	0.302803948	0.099502097
Pyridoxamine phosphate	0.316659947	0.171507718	0.425045492
Pyridoxine	-0.054735636	0.877530066	0.143586431
Pyriftalid	1.012217572	0.03401654	0.264686735
Pyrimethanil	1.245562999	0.00881863	0.190413745
Pyrimidodiazepine	-0.497335162	0.492805607	0.081536578

Pyrrole-2-carboxylic acid	0.251926844	0.335209294	3.510873176
Pyrrolidonecarboxylic acid	-0.729588653	0.052274788	1.870160376
Qing Hau Sau	1.658559915	0.009305552	0.266542311
Quercetin	0.996342476	0.09558362	0.162399655
Quetiapine	-0.551584747	0.436941323	0.078339085
Queuine	-0.447505871	0.597503892	0.055008218
Quinaldic acid	0.67664363	0.093497277	0.115176345
Quinapril	0.617580424	0.017006017	0.590265983
Quinapril hydrochloride	-0.51154631	0.630881543	0.024673255
Quinate	-0.57616869	0.100425271	0.2829894
Quinestrol	0.304637258	0.46673291	0.091452425
Quinmerac	1.402426347	0.05651243	0.278932615
Quinolinic acid	1.255879354	0.245924564	4.294670622
Quinone	0.749238016	0.001232416	0.310995801
Rabeprazole	-0.095878035	0.788180994	0.047592796
Raffinose	0.516955686	0.13302517	0.28245869
Repaglinide	-0.011237496	0.960863696	0.044899186
Rescinnamine	-1.83697731	0.008787345	0.242521059
Resiniferatoxin	0.999664518	0.027547471	0.175885914
Resolvin D2	-0.043665952	0.840127629	0.097548097
Resveratrol	0.17007208	0.558636948	0.019875934
Retinol	0.378089535	0.202464788	0.749320335
Retinoyl b-glucuronide	0.01160397	0.979032641	0.0305491
Rhein	0.103255378	0.798551997	0.030660711
Rhodomycin D	0.464325625	0.25793709	0.089130185
Ribavirin	-0.088606864	0.764452627	0.075002801
Riboflavin	0.225834616	0.803787027	0.045453392
Riboflavin reduced	0.76820234	0.051893954	0.275382816
Ribose 1,5-bisphosphate	0.014628926	0.957503852	0.096187684
Rifampicin	0.324847238	0.216846838	0.09063485
Rifamycin B	0.087310531	0.795162682	0.019839892
Rimsulfuron	-0.003188426	0.991083962	0.00661145
Rinderine	-2.971641518	0.144042204	0.217219489
Risperidone	0.28275756	0.257774134	0.056642266
Rotenone	-0.407105726	0.50303323	0.045518713
Rutamarin	0.109230899	0.811377094	0.025839806
S-Adenosylhomocysteine	0.119452216	0.710856023	0.07482683
S-Adenosylmethionine	0.294620886	0.176738469	1.029818585
Sakuranetin	0.492381959	0.140997227	0.142289845
Salannin	-0.614031125	0.437115905	0.112788994
S-Allylcysteine	0.627233128	0.043582469	0.86432442
Salmeterol	-0.600031926	0.221511192	0.10607756
Sanguinarine	-0.562383022	0.633632378	0.089097935

Sarsasapogenin	0.134185595	0.723105636	0.053342419
Scarlet Red	-0.205861618	0.792064277	0.073836435
Scoparone	0.318797975	0.24996541	0.245474895
Scopoletin	0.414231809	0.181523545	0.127234362
Scytophycin C	5.269293389	5.41E-08	0.482560451
SDS	0.46200124	0.108301059	1.24177555
Sebacic acid	0.394079071	0.108223087	0.490105614
Sedoheptulose	0.349075208	0.334383399	0.073312239
Selenomethionine	-0.244757485	0.819628023	0.124562226
Semustine	0.973608662	0.030284987	0.168118107
Senecionine	-0.282013347	0.545535704	0.335154994
Serotonin	0.516743927	0.056567514	0.367749932
Sertraline	-0.841079955	0.234580988	0.142042506
S-Formylglutathione	-0.016248887	0.97373724	0.012906985
S-Glutathionyl-L-cysteine	-1.424928609	0.066168972	0.229396041
S-Hexyl-glutathione	0.402276082	0.299315404	0.48558951
Sibutramine	0.66072254	0.4180231	0.102470043
Sildenafil	-0.064385369	0.782866731	0.033068093
Simazine	0.480619418	0.171082204	0.34874132
Simeconazole	0.96338234	0.011249271	0.127275583
Simvastatin	-0.108006436	0.871402031	0.029420548
Sinapic acid	1.227981744	0.002938688	0.224632806
Sinapine	-0.363328962	0.680146293	0.017881011
Sinapyl alcohol	0.100898977	0.825265723	0.001840738
Sisomicin	-0.435656585	0.193951432	0.315854731
S-Methyl-L-methionine	0.615745453	0.358326098	0.15277282
Sodium deoxycholate	-0.195778619	0.597299616	0.5975745
Sotalol	-0.091733277	0.763947134	0.009123766
Sparfloxacin	0.080137489	0.89928969	0.045667282
Spermidine	0.209670102	0.586537273	0.767766899
Spermine	-1.650882888	0.117193253	0.609824641
Sphinganine	0.196485509	0.528900988	0.114777365
Sphingosine	-0.156891497	0.529756901	0.137684613
S-Ribosyl-L-homocysteine	0.473023357	0.220304267	0.077100748
Stearic acid	-0.072704898	0.853303767	0.131707821
Sterigmatocystin	-0.114132163	0.824497556	0.058605475
Streptidine 6-phosphate	0.799847024	0.687025012	0.068101636
Streptozocin	0.326620422	0.454514989	0.100951386
Strychnine	0.485063695	0.515799504	0.088976397
Styrene Oxide	0.380694904	0.297355124	0.275340891
Suberic acid	0.285474456	0.249433269	0.328915851
Succinic acid	0.833579682	0.022929802	0.542929275
Sucrose	0.52825857	0.244756633	0.080028889

Sudan I	0.721809582	0.041041926	0.184249225
Sufentanil	0.494527079	0.193648988	0.109222005
Sulconazole	0.476502083	0.072951237	0.114822713
Sulfadiazine	0.97399857	0.015518401	0.210845959
Sulfamethazine	0.328787065	0.22395147	0.11660875
Sulfamethizole	0.435626225	0.061459509	0.148842919
Sulfamethoxazole	0.627310702	0.06699027	0.181066598
Sulfate	-0.520568519	0.272178408	0.213809213
Sumatriptan	-0.132892576	0.718062814	0.061943644
Tartaric acid	0.52057972	0.337029965	0.776437221
Taurine	0.413002657	0.11537482	0.74071242
Taurocholic acid	-1.08883328	0.290676157	0.114839843
Taurohyocholate	-0.342998174	0.520630155	0.431088477
Taxifolin	0.695301113	0.177775842	0.086847597
Taxusin	0.341494042	0.637637128	0.148241434
Telmisartan	-1.229058169	0.049511209	0.481034121
Temazepam	0.362693568	0.178120961	0.153895801
Tentoxin	0.484227028	0.068594928	0.214329891
Terbufos	0.26023484	0.262272467	0.083883742
Testosterone cypionate	0.038742021	0.885435976	0.049943255
Tetracaine	0.396199003	0.375076265	0.078628332
Tetracenomycin F1	0.612733058	0.076881652	0.093204108
Tetracenomycin F2	0.116666622	0.643223248	0.108701493
Tetrahydrocortisone	-0.094712878	0.803828368	0.075124681
Tetrahydrodipicolinate	-0.790300668	0.076036016	0.687039913
Tetrahydrofolic acid	0.049780473	0.912054351	0.003174638
Tetrahydroharmine	-0.013675069	0.956636355	0.004942503
Tetrahydropteridine	-0.011286029	0.965040251	0.010823591
Tetrangomycin	1.62001092	0.10977808	0.173510841
Thalsimine	-0.42293462	0.430984932	0.104279245
Thapsigargin	-0.521753354	0.343342035	0.668708186
Theophylline	-0.797011812	0.400581811	0.314925265
Thiamethoxam	-0.192815354	0.777792799	0.370688269
Thiamine	0.124622982	0.721588263	0.121267312
Thiamine aldehyde	-0.571971574	0.459425225	0.18620751
Thiamine pyrophosphate	-0.115555511	0.768214823	0.028790934
Thiazole	0.915161763	0.05457715	0.130946944
Thidiazuron	-0.085971941	0.773151369	0.019866575
Thiocyclam	0.452252148	0.327868032	0.070185566
Thiodicarb	0.141275405	0.635415732	0.04039565
Thiotepa	0.275141496	0.453560791	0.179812995
Thymidine	0.29460101	0.580259825	0.070789118
Thymine	0.264508037	0.36794311	0.609814149

Thymyl acetate	0.081213986	0.693888458	0.111175398
Thyrotropin releasing hormone	0.785170021	0.105148662	1.03319713
Thyroxine	0.772619395	0.009223709	0.250311617
Tiamulin	-0.516568112	0.340766755	0.178236857
Timolol	-0.054136915	0.881711909	0.047488454
Tobramycin	0.147753051	0.631871039	0.218324699
Tolterodine	0.778352678	0.406352912	0.27335337
Topiramate	0.45378291	0.467138516	0.041925697
Tramadol	4.070440173	0.168607455	0.593220788
Trandolapril	-0.758191213	0.467681237	0.083399678
Tranexamic Acid	-0.339300236	0.316015774	0.139632522
trans-Cinnamoyl beta-D-glucoside	0.083638201	0.761016247	0.130416844
Tranylcypromine	-0.133135058	0.670332617	0.027499164
Trehalose	0.179945695	0.51533705	0.267434727
Tri-allate	0.67955069	0.486543389	0.093101572
Tributyl phosphate	0.286939083	0.331199761	0.077135015
Trientine	0.862874217	0.002256075	0.327783178
Triethanolamine	0.451648897	0.387027063	0.038924099
Triethylamine	1.093606849	0.037469999	0.431011442
Trimethoprim	1.012141295	0.013658562	0.730692274
Trimipramine maleate	0.137754803	0.571134738	0.024241959
Trioxsalen	-0.174244593	0.622472126	0.063511185
Triphenyl phosphate	0.448678797	0.224611299	0.175832882
Tris(1-aziridinyl)phosphine oxide	-0.950417197	0.359836306	0.284379051
Tropate	0.179123072	0.535183671	0.038123896
Tropatepine	-0.103434353	0.688999897	0.138045754
Tubocurarine	0.164751797	0.681832294	0.019435826
Tylactone	1.154819254	0.067289525	0.14611295
Tylosin	-0.093499588	0.785494031	0.079178714
U50488	2.771389939	0.068016747	0.110036908
UDP-L-rhamnose	-0.071885254	0.921763901	0.001990788
Uracil	0.355539466	0.249768738	0.144806591
Uracil 5-carboxylate	0.041101179	0.892498562	0.080186869
Uracil mustard	-0.078113966	0.791133837	0.032039632
Ureidopropionic acid	2.196933592	0.299084254	0.733099273
Uridine	0.382037815	0.185125618	0.255303999
Uridine diphosphate-N-acetylglucosamine	-0.158489324	0.57885102	0.124911249
Urocanic acid	-0.005095914	0.988363174	0.025273011
Ursodeoxycholic acid	0.136482642	0.708554662	0.015124236
Usnic acid	0.265662857	0.283032494	0.06796975
Valeric acid	0.488148537	0.280240005	0.188307655
Valpromide	0.424220104	0.058265902	0.239591946
Vanillin	-0.802538546	0.196392198	0.187173317

Vanylglycol	0.380472779	0.158460218	0.467148318
Vardenafil	0.143120949	0.578948566	0.000530432
Vellosimine	0.06011574	0.850714783	0.002302065
Venlafaxine	1.461026603	0.004927505	0.173584107
Verapamil	-0.576469171	0.250468712	0.278758531
Veratramine	0.021379895	0.956413319	0.127320582
Veratridine	-0.739110247	0.165442287	0.239258581
Vinclozolin	4.622104548	0.271367749	0.992865094
Vindoline	-0.421723445	0.518560822	0.202956503
Visnagin	-0.734072504	0.164628307	0.076188861
Vitamin K1	0.368816492	0.587141894	0.10927523
Vomitoxin	-0.354445249	0.493278451	0.066463794
Xanthine	0.523625582	0.217840858	0.433226541
Xanthopterin-B2	0.651141718	0.097144202	0.130192295
Xanthosine	3.492991021	0.167343985	0.229593976
Xanthoxin	0.247831678	0.510713522	0.024832455
Xanthurenic acid	1.087028922	0.063327628	0.221368219
Xylometazoline	0.290601797	0.31754674	0.079374977
Yamogenin	0.114576371	0.751023713	0.025044934
Zalcitabine	0.495142416	0.105216638	0.219069767
Zaleplon	0.188870604	0.635556187	0.037783974
Zonisamide	0.252309625	0.272443508	0.117145103
Zoxamide	0.223091355	0.813599047	0.079305168
Zymosterol intermediate 2	0.86606448	0.108226055	0.329641477
