# Supplementary material for: Serum metabolomics study of narcolepsy type 1 based on ultra-performance liquid chromatography–tandem mass spectrometry
Source: Amino Acids. 2023 Sep 10;55(10):1247–59. doi: 10.1007/s00726-023-03315-z (PMC10689557; doi:10.1007/s00726-023-03315-z)
Supplement: Supplementary file 2 — Supplementary file2 (PDF 298 KB) [file 726_2023_3315_MOESM2_ESM.pdf]

Supplementary Table S2

Name	Log2_FC	Pvalue	Vip
Xanthine	0.942931595	0.214134206	1.489375609
Uridine	0.379656159	0.154361444	0.87978838
Uric acid	-0.264084168	0.17697516	5.750332719
Thymidine	0.110829372	0.183347476	0.39450327
Succinic acid	0.310730606	0.143290834	0.762694339
Stearic acid	0.172933406	0.53547607	0.604044311
Salicyluric acid	-1.413225774	0.410683385	0.156927768
Salicylic acid	-0.473693147	0.582409064	0.358646646
S-Adenosylmethionine	0.034068183	0.965906081	0.0800343
Pyroglutamic acid	0.317270542	0.34577843	0.705125354
Putrescine	-0.166841381	0.702592525	0.488848731
Phenyllactate	0.709521526	0.018089291	0.96054212
Phenyl acetate	0.092251082	0.174445636	0.675918276
Phenol	-0.442547982	0.515343741	0.194180349
Oxoadipic acid	2.94149155	0.004734457	3.063986131
Oxalacetic acid	-0.383554278	0.246814406	2.562262736
N-Acetylglutamic acid	0.28773154	0.112402655	0.51006521
N-[(3a,5b,7a)-3-hydroxy-24-oxo-7-(sulfooxy)cholan-24-yl]-Glycine	-0.036158967	0.95748593	0.015180398
Methyldopa	0.226107852	0.019740331	0.563491926
Methyl jasmonate	-0.04615344	0.441039643	0.193479888
Mesaconate	0.129643016	0.481947291	0.293762837
Mannitol	0.113260771	0.495923889	0.246825215
L-Phenylalanine	0.287742014	0.055604397	4.905439023
L-Norvaline	0.199390277	0.629989675	0.275224333
L-Methionine	0.172008241	0.533958858	0.694124528
L-Malic acid	0.483970822	0.063123426	0.521906293
L-Cystine	-0.353330694	0.655002468	0.255348531
L-Arginine	0.270966852	0.193950423	1.578977193
Isopropylparaben	0.296334548	0.191483113	0.371918068
Inosine	-0.575550206	0.562927375	2.365977006
Hypoxanthine	-0.431181794	0.49712574	0.844995193
Hippuric acid	-0.087644248	0.837612638	0.493843198
Glycocholic acid	-0.706297176	0.189756868	1.346050163
Glycochenodeoxycholic acid	-0.014152867	0.985565998	0.073961949
Gluconic acid	0.512061314	0.130617237	1.936815708
gamma-Glutamylcysteine	0.122624775	0.119993915	3.280697519
Fumaric acid	1.306579981	0.050802915	1.217789538
Folic acid	0.103853591	0.906348386	0.108352033
Erythritol	0.717561515	0.010019828	1.918095543
Epiandrosterone	0.078176931	0.470458593	0.631490817

Docosapentaenoic acid (22n-3)	-0.028059215	0.947650011	0.056474201
D-Mannose	0.717108427	0.433858046	0.818003063
Dibutyl phthalate	-0.175195059	0.316665419	0.208141517
D-Glucuronic Acid	0.260400155	0.317831767	0.804968312
D-Erythritol 4-phosphate	-0.61008514	0.490638465	0.335444458
D-Erythritol 1-phosphate	-0.627954559	0.471934339	1.201774748
Deoxyguanosine	0.51321229	0.004992039	0.996169748
Deoxycholic acid	-0.402741932	0.358781647	0.506802981
dCMP	0.253250814	0.008841503	0.473465148
Creatine	0.587757113	0.20309498	0.926899166
Cortisol	0.161044513	0.058737156	0.370352164
Bovinic acid	-4.551177605	0.092141834	14.58701843
Azelaic acid	0.393625328	0.091380005	0.777031791
alpha-Ketoisovaleric acid	0.109080919	0.389617717	0.660987721
Allocholic acid	0.102178049	0.881112133	0.058265627
Acetylcholine chloride	0.079411281	0.283968214	1.576202926
6-Hydroxynicotinate	0.386789094	0.082058775	0.75488792
5-Hydroxyindoleacetic acid	0.398346936	0.108788899	0.777834239
5-Aminopentanoic acid	-0.034953717	0.906061747	0.217306876
3-Methylindole	0.43886967	0.226110128	1.447811057
3,4-Dihydroxybenzeneacetic acid	-0.384955273	0.584385643	1.354975093
2-Oxoarginine	0.58071849	0.014311453	0.677107291
2-Hydroxyphenylacetate	0.00591284	0.96823353	0.053526819
2,4-Dinitrophenol	-0.048373641	0.882409686	0.086690075
13S-hydroxyoctadecadienoic acid	0.245303268	0.518470361	0.233453244
12-KETE	2.588922156	0.166581052	0.809000518
12-Hydroxydodecanoic acid	1.365451222	0.006710814	1.582274755
(R)-3-Hydroxybutyric acid	0.607151969	0.077422882	1.53929357
Zinniol	0.257637415	0.360359633	0.299848934
Zingerone	0.227130663	0.052069757	0.964567545
Zidovudine	0.8121095	0.352642072	0.12680696
Zafirlukast	0.303899724	0.397785068	0.049874168
Yohimbine	-0.035918366	0.945271233	0.033581656
Xanthylic acid	-0.110586454	0.463871824	0.054732133
Xanthurenic acid	0.33654126	0.302266772	0.130986288
Xanthoxylin	0.818494791	0.211481278	0.224200978
Xanthosine	-1.104603759	0.019195285	0.378402035
Xanthopterin-B2	-0.343030585	0.078270988	0.269084688
Xanthohumol	-0.96155638	0.200924966	0.206139096
Xanomeline	0.557111508	0.062604222	0.21454936
Withaferin A	0.401368256	0.35045908	0.141293898
Vulgarin	1.199153304	0.04524809	0.369549616
Virginiamycin	-0.594276059	0.27091772	0.089405093

Vincristine	0.283183403	0.291413963	0.322063039
Vidarabine	0.458602938	0.458885641	0.216880293
Veratramine	-0.388392885	0.341181384	0.12808808
Vanillylmandelic acid	-1.169248715	0.00221707	0.551731164
Valeric acid	0.012187543	0.929331443	0.009714583
Valdecoxib	1.015511095	0.089177067	0.428105057
Ursodeoxycholic acid	-0.16511281	0.754378995	0.198703698
Uridine diphosphate-N-acetylglucosamine	-0.361359222	0.275890751	0.159486729
Uridine diphosphategalactose	0.214927025	0.339704154	0.080314558
Uracil mustard	0.470178702	0.329044837	0.089212107
Uniconazole P	-0.360791298	0.46137707	0.060482554
Undecanoic acid	0.073451303	0.372786743	0.085179868
UDP	0.125659036	0.163882403	0.085347521
Tubocurarine	-0.092369662	0.930447081	0.027562876
Tryptophanol	-1.102099963	0.02167302	0.477648857
Tryptophanamide	0.498110666	0.038116321	0.135842983
Triticonazole	0.08692299	0.545038527	0.31226557
Triphenyl phosphate	0.427589456	0.170451545	0.243589934
Triflumuron	-0.770817726	0.035849258	0.517872957
Triamcinolone acetonide	-0.370767835	0.567368636	0.104309037
Triamcinolone	-0.134626254	0.827473338	0.034770271
Tremulacin	-0.052251305	0.92849773	0.00544073
Trehalose	0.207000876	0.53836619	0.112925628
trans-Zeatin riboside	0.366960321	0.120175225	0.244257488
trans-Piceid	0.094318856	0.819291644	0.03029769
trans-Ferulic acid	-0.040621574	0.63428431	0.079710529
trans-Aconitate	-2.940752294	0.160220388	0.956636488
Tos-Lys-CH2Cl	0.402603938	0.54299036	0.133358989
Toluene-4-sulfonate	0.22005529	0.213326135	0.391832709
Tolmetin	0.003593887	0.987608699	0.003531044
Tobramycin	-0.032058979	0.934926493	0.02622359
Thyroxine	0.463239366	0.069724516	0.182050306
Thiophanate-methyl	0.506181629	0.20456917	0.10068902
Thidiazuron	-0.1036165	0.808766792	0.103891343
Thiazole	0.237750149	0.648293102	0.171258256
Thiamine monophosphate	0.069701172	0.462344785	0.035947034
Thiacloprid	-1.295516768	0.073755797	0.778911382
Thiabendazole	0.135766457	0.074515659	2.059904107
Theophylline	1.096168158	0.347226277	0.468422001
Thapsigargin	0.366870226	0.014951256	0.241541941
Thalidomide	0.559133061	0.071949451	0.305113776
Tetracosanoic acid	0.221578296	0.408009951	0.221224024
Tetrabromobisphenol A	0.17703485	0.3240495	0.293614239

Teflubenzuron	-0.41658216	0.031428717	0.337924628
Tebuthiuron	-1.220922669	0.018262761	0.464900245
Tebufenozide	0.518229828	0.428333035	0.069437888
Taxifolin	0.513495265	0.054763614	0.095089182
Taurocholic acid	-0.883669662	0.091993748	0.516620742
Taurochenodesoxycholic acid	0.161210136	0.8247382	0.007032296
Taurine	0.36420674	0.106565987	0.812770577
Tartaric acid	-1.064434747	0.433500347	0.604055522
Tamsulosin	-0.219426243	0.744940682	0.089251013
Tacrolimus	0.927344387	0.011756088	0.377563002
Tacrine	0.295640415	0.413753026	0.119851868
T2 Toxin	-0.18499514	0.815745806	0.03044791
Syringic acid	-0.158440274	0.670179242	0.258079888
Suspensaside	0.709973657	0.19233287	0.176000499
Sumatriptan	0.21043048	0.015216333	0.249059493
Sulpiride	-0.085935599	0.829578019	0.051470819
Sulfoacetate	0.175928182	0.351291265	0.094449551
Sulfluramid	0.385447268	0.04138272	0.466352192
Sulfisoxazole	-0.533977495	0.022132795	1.312598786
Sulfinpyrazone	-0.038837511	0.940424684	0.013803484
Sulfathiazole	0.516220832	0.000216368	0.278710746
Sulfasalazine	-0.441691726	0.696348112	0.062433887
Sulfamethoxazole	0.731440374	0.007276932	0.479334705
Sulfamethizole	0.178737118	0.048132153	0.342004446
Sulfamethazine	-0.079338534	0.920128667	0.056941983
Sucrose	0.740120039	0.105337212	0.701648079
Sucralose	0.315811861	0.107069197	0.131900634
Succinyl sulfathiazole	-1.26166873	0.174612716	0.181211471
Succinate semialdehyde	-0.217653536	0.502477066	0.222141577
Suberic acid	-0.147260398	0.410634371	0.258907623
Streptozocin	-0.017901961	0.980825592	0.009070993
Streptonigrin	0.918458271	0.215526057	0.135403203
Streptomycin	-0.381422822	0.475429002	0.151586755
Stevioside	0.300830543	0.504545234	0.052530854
Spironolactone	-0.335674908	0.425527831	0.107139763
Sphingosine 1-phosphate	-0.539974586	0.199798446	0.45065058
Sphinganine	-0.066548724	0.438169566	0.044419906
Soyasaponin I	0.136869508	0.648391725	0.033208928
Sorbitol	-0.159628992	0.883145673	0.302757381
S-Lactoylglutathione	-0.404667508	0.017593915	0.104847727
Sinomenine	-0.593273109	0.252114491	0.13972287
Sinigrin	0.125283251	0.200353192	0.28900586
Sinensetin	0.192884671	0.638637575	0.066380631

Sinapyl alcohol	0.411681996	0.356813708	0.272665354
Sinapic acid	-0.25354648	0.522352489	0.067466767
Sildenafil	0.917968589	0.057686356	0.835940211
Shikimic acid	-0.265014786	0.805563892	0.097807255
S-Hexyl-glutathione	0.3281286	0.600469093	0.07039981
Shanzhiside	-0.490764583	0.306261688	0.12924035
Sedoheptulose 7-phosphate	0.052143423	0.564769611	0.167388224
Secbumeton	-2.911081633	0.306708193	0.448845924
SDS	-0.399801625	0.24056837	0.090299538
Scytophycin C	0.274509594	0.203820225	0.264359382
SC-58125	1.448616871	0.313440118	0.263567795
Saucernetin	0.501481515	0.559749448	0.080100555
Sarcosine	0.452275999	0.038280658	0.663767638
Salvinorin A	-0.015007201	0.985076163	0.010927934
Salidroside	0.662748208	0.263504392	0.068754098
Salicin	0.781309018	0.057642616	0.142998525
Sakuranetin	0.515673105	0.036367736	0.331450697
S-Adenosylhomocysteine	-0.141225984	0.545136351	0.057913972
Saccharin sodium anhydrous	-0.478714577	0.647780275	0.630062209
Saccharin	0.020181328	0.954895408	0.01346143
S-(4-Azidophenacyl)glutathione	0.454721784	0.121623271	0.440057991
Rotenone	-0.046566238	0.909261002	0.042856139
Roridin A	-0.187278766	0.786439292	0.027194322
Ritonavir	0.038243296	0.864972987	0.012032948
risedronate	0.119137342	0.172221219	0.147631055
Rimsulfuron	0.045497298	0.895215131	0.069064304
Rifamycin	-1.829725188	0.020678759	0.349784259
Rifampicin	0.288737995	0.505421977	0.076801754
Ribostamycin	0.78469997	0.291298966	0.090124228
Ribose 1,5-bisphosphate	-0.022834858	0.961541512	0.068931706
Ribitol	0.268558205	0.352419607	0.212058602
Resveratrol	0.19457493	0.745973487	0.009254451
Reserpine	0.255747726	0.097474645	0.094585835
Rescinnamine	-1.592283028	0.004625089	0.171716213
Repaglinide	-1.005860793	0.012862867	0.483084999
Ranitidine	-0.031104802	0.963004324	0.022589975
Raloxifene	1.048981397	0.149916798	0.321601846
Rabelomycin	0.008979375	0.980311254	0.028347746
Quinolinate	0.30106815	0.149381506	0.394860023
Quinmerac	1.903153467	0.08569256	0.196717347
Quinine	1.516123113	0.298155913	0.260561494
Quinapril	-1.216984362	0.032246918	0.404374725
Quinacrine	0.083589471	0.869707238	0.059502882

Quassin	-0.213497952	0.65715289	0.129263189
Pyrimethanil	-1.08858323	0.000734275	0.342915322
Pyridoxamine 5'-phosphate	0.394298822	0.028379717	2.823978024
Pyridoxal 5'-phosphate	0.543639801	0.505550803	0.051836253
Pyridaben	0.352974767	0.406866849	0.119217207
Pyrethrosin	1.167838183	0.069695484	0.156355193
Pymetrozine	0.151916705	0.194946751	0.09524444
Purpurogallin	1.803155171	0.000293335	0.3899768
Purpurin	0.870222043	0.002520899	0.167491447
Puromycin	0.376502307	0.370014056	0.116665676
Pterin	0.770788118	0.007866987	0.21589867
Protoveratrine A	0.971649775	0.008470707	1.508505098
Protionamide	2.707189652	0.171824039	0.46442208
Prostaglandin F2a	-0.149268725	0.76686972	0.083418597
Propyzamide	-0.382348697	0.649454985	0.197583322
Propanil	0.212863488	0.565114318	0.045129388
Procymidone	0.095813712	0.545578683	0.145610532
Probucol	-3.454036006	0.017755387	0.507303254
Prednisone	0.055522978	0.755632291	0.051021062
Pravastatin	-0.694250018	0.213056267	0.262536394
PQQ	0.141171535	0.604259926	0.023729657
p-Phenolsulfonic acid	-0.421605238	0.514272812	2.129897444
Podofilox	1.1141844	0.160640187	0.161627107
Piroxicam	0.06988153	0.659058795	0.019733577
Piplartine	-2.126884835	0.185036758	0.2118282
Pipecolic acid	-0.140762663	0.505663808	0.076649381
Pinolidoxin	0.06784246	0.911685183	0.024270726
Picrotin	-0.26799792	0.497725919	0.101318404
Picein	-0.133593023	0.599667229	0.155826838
Phosphorylcholine	0.52812691	0.092708546	0.371577257
Phosphonoacetate	-0.941414705	0.007333936	0.665803345
Phosphocreatine	0.59552062	0.18528441	0.307266682
Phospho-anandamide	0.690953119	0.091016154	0.220736365
Phloretin	-3.710638628	0.104164779	0.513577566
Phenytoin	0.659301287	0.057232816	0.146064001
Phenylpyruvic acid	0.18759235	0.580381159	0.028754887
Phenylbutazone	0.2229874	0.401885363	0.452988326
Phenylacetylglycine	-0.32329171	0.491202086	0.110189805
Phenylacetaldehyde	0.021123371	0.913066261	0.012113588
Phenthoate	0.209920321	0.31367394	0.188056209
Phenolphthalein	0.170261412	0.2819053	0.130170936
Petunidin 3-glucoside	0.165137911	0.119273299	0.077339375
Perillyl alcohol	-0.333384406	0.451387379	0.106794764

Perillic acid	0.331842733	0.511095441	0.335123035
Pentobarbital	-0.53179673	0.030701351	0.260608411
Pentachlorophenol	-1.57953798	0.155657269	0.44744384
Penicillin V	0.550301808	0.201355236	0.230300878
Penicillin G	0.440934256	0.083236175	0.149186496
Pendimethalin	1.719191024	0.009150798	0.356954291
Pencycuron	0.308824078	0.200076959	0.180931166
Penciclovir	-0.318078147	0.471386522	0.0704894
p-Cresol	-0.088462441	0.890357222	0.137310212
Paxilline	1.179071793	0.037637998	0.433547039
Paraoxon	0.271509743	0.00428938	0.355877405
Pantothenol	0.133583369	0.238295089	0.179907784
Pantothenic acid	0.508057846	0.117628017	0.495640853
Oxyphenbutazone	1.95695853	0.00704665	0.374596513
Oxycodone	-0.361252066	0.01097589	0.184978221
Oxidized glutathione	0.140425765	0.161683316	0.254088917
o-Toluate	3.893002864	0.039838472	2.511617816
O-Succinyl-L-homoserine	0.235347342	0.268970132	0.1543262
Ostruthin	0.307224063	0.451288309	0.25638955
Osajin	-0.861296869	0.099587715	0.353423921
Ornithine	0.682471683	0.154768146	0.190809447
Ophthalmate	0.590512074	0.118786544	0.207997871
O-Phosphotyrosine	-0.042651968	0.753804156	0.033603466
Omethoate	-0.582556512	0.067818572	0.863293497
Omeprazole	0.107389502	0.175243342	0.076393484
Olopatadine	0.407493368	0.224516188	0.434256422
Olmesartan	0.247005724	0.669922324	0.037139461
Olanzapine	0.198897449	0.305092438	0.587983058
Ofloxacin	0.859218694	0.127939187	0.246974327
Octopine	0.331959585	0.630976048	0.027219337
n-Propyl gallate	0.331880724	0.015419272	0.294933712
Norspermidine	0.060259302	0.543076492	0.093215762
Norfloxacin	-0.071117589	0.814273771	0.034939827
Norethindrone acetate	0.104798885	0.818326929	0.230459888
Nonadecanoic acid	1.042340728	0.010164377	1.194712267
Nomilin	0.185488909	0.715716927	0.044542546
Nodularin	0.064331853	0.761950694	0.033433546
Nobiletin	1.050587215	0.015374158	1.619453065
N-Methyl-D-aspartic acid	0.607825325	0.004931676	0.390916676
Nizatidine	0.140626586	0.758927858	0.074565504
Nitrofurazone	-0.549343722	0.038606378	0.683374651
Nitrofurantoin	0.822097921	0.240412485	0.338453606
Nitrofen	0.530296288	0.191335512	0.163060781

Nimodipine	-0.162130469	0.725805386	0.017114481
Niflumic Acid	0.152924812	0.270270167	0.111976918
Nifedipine	0.010719155	0.925360565	0.006528094
Nicotinuric acid	0.537313887	0.115856202	0.170245143
Nicotinic acid mononucleotide	0.16745608	0.256285208	0.047139987
Nicotinic acid	-0.01097248	0.974020332	0.018373055
Nicotinamide ribotide	0.309279974	0.476426612	0.178587216
Nicotinamide riboside	-0.849443068	0.201978162	0.171835766
Nicosulfuron	0.410886958	0.258856845	0.264405593
N-Glycolylneuraminic acid	-0.192660016	0.084616882	0.089339649
N-Formyl-L-methionine	0.019145323	0.896877651	0.000957529
N-Formyl-L-aspartate	-0.208930922	0.476185213	0.103041244
Neburon	0.716129186	0.212637689	0.16205211
Nateglinide	1.42429645	0.013099045	0.690645059
Naringenin	-0.280391718	0.486530187	0.126811106
N-Amidino-L-glutamate	0.427073457	0.179691286	0.479834149
N-Alpha-acetyllysine	0.177783124	0.40071482	0.22509857
N-Acetylneuraminic acid	0.705933187	0.030767283	2.099396998
N-Acetylmuramate	-0.105692043	0.842274541	0.078140966
N-Acetylmannosamine	0.002170468	0.98583915	0.013795961
N-Acetyl-L-tyrosine	0.124667702	0.773105844	0.170801981
N-Acetyl-L-phenylalanine	0.05553168	0.81246273	0.025347469
N-Acetyl-L-leucine	-0.474288598	0.005775583	0.281201577
N-Acetyl-L-glutamine	0.624976047	0.268398395	0.127926056
N-Acetyl-L-aspartic acid	0.175682709	0.122426288	0.369157755
N-Acetylhistidine	0.632667687	0.224906346	0.262115844
N-Acetyl-D-glucosamine	0.476904455	0.072714789	0.349307085
N-Acetylaspartylglutamate	0.172201014	0.266601676	0.147987293
N-Acetylanthranilate	0.49281053	0.01594817	0.224809516
N-Acetyl-alpha-D-glucosamine 1-phosphate	0.278973035	0.040882602	1.035571539
N4-Acetylsulfamethoxazole	-3.807667859	0.057993055	1.080597031
N2-gamma-Glutamylglutamine	0.347550603	0.286431329	0.105595997
N-(L-Arginino)succinate	-0.801710939	0.11996138	0.175442045
myo-Inositol	0.042867971	0.752433809	0.645472452
Muramic acid	0.156115328	0.347271946	0.128221721
Mupirocin	0.75568987	0.478827834	0.748724998
Moupinamide	-0.482764807	0.190194724	0.423914852
Morphine	-0.644200771	0.368643503	0.138511354
Mometasone furoate	-0.031685354	0.947184723	0.003147223
Mitragynine	-0.388883939	0.599481063	0.285572917
Mitomycin	0.017824285	0.903699575	0.080001407
Minocycline	0.728916495	0.206582261	0.325786729
Milbemectin	0.060334585	0.816527434	0.030814025

Miglitol	0.815816691	0.218852736	0.15504506
Midazolam	0.48072249	0.069597797	1.022843965
Metoxuron	0.269774199	0.220825552	0.054634572
Metosulam	-0.03748766	0.744313753	0.104492767
Metolazone	0.30973963	0.119961979	0.116840717
Metobromuron	0.12012773	0.843283541	0.006424661
Methoprotryne	0.071838297	0.899404826	0.002912859
Methionine sulfoximine	0.091182941	0.520898321	0.397059291
Methazolamide	0.909359543	0.199940366	0.291945395
Metamitron	-0.193250001	0.555208329	0.034342431
Mesobilirubinogen	0.788887242	0.301565643	0.106833187
Meloxicam	-0.366323603	0.732205582	0.094056176
Melibiose	-0.609102121	0.18872412	0.381278575
MeIQx	-0.190588997	0.190326487	0.073768536
Mefenamic acid	-1.601669221	0.053719366	0.228938798
Meclofenamate sodium	0.339055599	0.130748854	0.130608924
Mebendazole	0.296668267	0.314369153	0.191746257
MCPA	0.083017186	0.78877824	0.006503994
Maytansine	0.43613232	0.272618306	0.265583619
Manumycin A	-0.669098654	0.50368587	0.07181028
Malvidin 3-glucoside	0.19420551	0.183509605	0.644777463
Malonate	-0.185795764	0.473565442	0.129982952
Malaoxon	0.82482845	0.423760058	0.294479578
LysoPA(16:0/0:0)	0.776108855	0.34254836	0.299401816
Lupinine	0.271334985	0.028632682	0.111658983
Lumichrome	0.161453549	0.693043543	0.044778765
L-Tryptophan	-0.089813277	0.843517155	0.032333444
L-Threonine	0.228168687	0.043076089	0.836815043
L-Serine	-0.654591238	0.12162628	0.517716309
L-Ribulose	-0.228399832	0.451999803	0.159619645
L-Proline	0.027712218	0.858930158	0.039871682
Lovastatin	0.032066326	0.930114331	0.017253252
Lotaustralin	-0.582775461	0.151527843	0.34911476
Losartan	0.232128987	0.451348302	0.493974155
Lopinavir	-0.442274078	0.552127082	0.108280271
Lonchocarpol A	-0.670665697	0.273187096	0.488263883
Lomefloxacin hydrochloride	0.243248662	0.515295611	0.080891031
Loganin	0.161105801	0.690717721	0.095868193
L-Lysine	0.122971841	0.656871488	0.157003965
Linuron	3.530443983	0.321880301	0.414353652
Linoleate	0.749807071	0.114855431	6.366391828
Ligustilide	0.17270542	0.754367396	0.043090389
L-Iditol	0.375901934	0.056917798	1.49707918

L-Homocysteine	0.296407056	0.440674138	0.152468099
L-Histidine	0.075153174	0.458744744	0.164768174
L-Glutamic acid	0.48969016	0.019415174	1.394959384
L-Fucose	0.294159388	0.130387085	0.162110134
Leukotriene F4	-1.365680663	0.024062724	0.256379815
Leukotriene D4	-0.629101999	0.002362382	0.259482017
Leukotriene C4	0.737313576	5.68E-05	1.168190742
Lenacil	0.001867286	0.995090848	0.049516046
L-Dopa	0.276699418	0.269024184	0.103014252
L-Djenkolic acid	-0.423617138	0.579759689	0.189350678
Latrunculin B	-0.059057852	0.823617386	0.039137233
L-Aspartic acid	0.473916164	0.123047566	1.92312439
L-Ascorbic acid, 6-octadecanoate	0.544831333	0.317853237	0.223158316
L-Arabitol	1.17900412	0.047311288	0.356766744
Lappaconitine	0.590109529	0.362078619	0.100097458
Lanosterin	-0.0340233	0.921658285	0.045767069
Lamivudine	0.325593245	0.823884402	0.021075896
L-Allothreonine	-0.025735012	0.92273244	0.022687089
Labetalol	-0.074194969	0.860424445	0.023286587
Kynurenic acid	0.5791458	0.047581687	0.27539372
Kinetin	1.24405978	0.008905821	0.162780791
Khellin	0.225663514	0.311091215	0.160971877
Kanamycin B	-0.142453526	0.70984526	0.072779912
Kanamycin	0.272015419	0.014940741	0.098291127
Kaempferol	-1.745369331	0.185132011	0.168569436
Kaempferide	0.775471115	0.065975225	0.977230952
Ivermectin B1a	-0.597463667	0.037152499	0.323458638
Itaconate	0.164798178	0.36611886	0.14507614
Isorhamnetin	0.140344978	0.209880286	0.111102595
Isoquercitrin	1.539640684	0.003598757	0.352246469
Isopentenyl adenosine	0.292493395	0.35627128	0.954414526
Isocitric acid	0.154607579	0.491945983	0.562278931
Isochlorogenic acid b	-3.400833947	0.260957956	3.88219229
Irinotecan	0.426493769	0.002365875	0.121845679
Irbesartan	-0.209996754	0.671829577	0.079787666
Iproniazid	-0.34388786	0.559314048	0.080308716
Ipecoside	-0.681292202	0.26140369	0.144201477
Iodoquinol	-0.067804661	0.510980418	0.045068032
Inositol 1,3,4,5,6-pentakisphosphate	-0.819010461	0.005935493	1.523154228
Indolepyruvate	-0.287644915	0.77806315	0.06949448
Indolelactate	1.338557229	0.508466183	0.176208906
Indolebutyric acid	-0.422214537	0.214595065	0.213601924
Indoleacetaldehyde	-0.305305496	0.257294473	0.118170441

Indole	0.028356126	0.836258815	0.060106972
Indapamide	0.185572341	0.15422428	0.751661088
Immunomycin	-1.430399838	0.012824093	0.658768065
Ifosfamide	0.08313321	0.623368566	0.044165952
Icariin	-0.562321845	0.186772965	0.115460998
Hydrogen phosphate	-0.215369594	0.452147215	0.364137174
Hydrocortisone succinate	-1.438116092	0.022419214	0.172502568
Hydrochlorothiazide	-0.222805126	0.440211867	0.066205576
HT-2 Toxin	-0.362531155	0.292426782	0.403748924
Homo-L-arginine	-0.060092855	0.76818143	0.087742085
Hexadecanedioic acid	0.68707389	0.129040325	0.337464811
Hesperetin	0.94990616	0.008542113	0.302797617
Heptenophos	-0.453568856	0.341867731	0.136609136
Heptanoic acid	-0.396744596	0.19402313	1.398147564
Hellebrin	0.595937636	0.007260042	0.650248345
HC-toxin	0.219281159	0.659876679	0.029600204
Halosulfuron-methyl	-0.493191452	0.070417789	0.283266094
GW 7647	-0.473767342	0.26511638	0.071504641
Guanine	0.600662532	0.317395285	0.192491507
Guanidoacetic acid	0.043756992	0.896956906	0.068908805
Grayanotoxin I	-0.113715133	0.811292845	0.060764875
Glycyrrhetinate	-0.438401135	0.417248784	0.161869951
Glycyl-leucine	-0.7034117	0.324927246	0.207333744
Glycyl-glycine	0.21982907	0.293375115	0.26676616
Glycocholic acid	-0.123507135	0.778976949	0.034785024
Glycitein	-0.333573043	0.242450559	0.186031375
Glyceric acid	0.637850744	0.050225328	0.910768003
Glutathione	-0.054543253	0.644356042	0.135761822
Glutaric acid	-0.058248084	0.691008724	0.20753145
Glutamylglutamic acid	1.245612592	0.000120085	1.078471088
Glucotropaeolin	0.126264407	0.403621595	0.100633615
Glucose 6-phosphate	0.645155348	0.10989867	0.261946923
Glucosamine 6-phosphate	-0.264955402	0.49276949	0.124376793
gluconasturtiin	1.617796802	0.371827948	0.323406417
Glucaric acid	-0.420234283	0.070846647	0.43196384
Glimepiride	0.847208359	0.397127815	0.119240893
Glaucarubin	-0.06121468	0.933047781	0.009868125
Gibberellin A9	-0.071803358	0.720189856	0.080422358
Gibberellin A8	-0.070972729	0.42820136	0.054138466
Gibberellin A124	-0.43807697	0.246478648	0.200150096
GF 109203X	0.344444449	0.454794614	0.343817296
Geranylgeranyl-PP	0.987469331	0.186253488	0.150322654
Geranic acid	0.129459679	0.237797568	0.105670682

Genistin	-0.550370586	0.292609095	0.175354134
Genistein	-0.856055785	0.229903777	0.091196964
Genipin	0.014222142	0.968524078	0.001185836
Geldanamycin	0.139953508	0.823435156	0.093416321
GDP-L-fucose	0.117067838	0.534000694	0.028724113
Ganciclovir	-0.028201699	0.958680973	0.016046195
Gamma-Tocotrienol	0.231191871	0.360725383	0.127446778
gamma-Glutamylalanine	0.33290957	0.098954277	0.41322719
Gallic acid	0.656447782	0.270033484	0.120414302
Galacturonic acid	-0.34686402	0.689844148	0.254620601
Galactonic acid	-0.711628276	0.270369808	0.081365393
Galactaric acid	-2.566635441	0.011034855	0.963202403
Fumitremorgin A	-0.230552027	0.343542075	0.168393154
Fucose 1-phosphate	0.11038894	0.133128406	0.313154412
Fructose-1P	1.54632181	0.02007841	0.693415402
Fructose 1,6-bisphosphate	0.161922513	0.714825031	0.765332502
Fraxin	-0.436865968	0.172530184	1.490153725
Fraxidin	1.304428295	0.162901872	0.245610369
Formononetin	-0.113553125	0.857176527	0.000786055
Forchlorfenuron	1.101988048	0.113804439	0.170833419
Fomesafen	-0.042551856	0.878463629	0.050856065
FMN	-1.032141691	0.081168512	0.609152557
FMLP	0.890841112	0.0090158	0.303710371
Fluvastatin	-0.035184981	0.921021269	0.018008996
Flutamide	0.119598397	0.570096714	0.128128319
Fluroxypyr	0.124568801	0.651730518	0.01266887
Flurecol	-1.096199781	0.184567565	0.234626755
Fluperlapine	0.685022759	0.027642261	0.155235589
Fluocinonide	-0.321789307	0.514073001	0.13716506
Flumioxazin	0.320304682	0.415674528	0.089545498
Fludioxonil	-1.061663631	0.074607527	0.392602449
Fluazinam	-0.072665323	0.865841876	0.239568696
Fluazifop	-0.12527344	0.737391499	0.070874226
Floxuridine	-0.272553688	0.573768062	0.061977133
Flecainide	-0.033157927	0.9375517	0.002637373
Fexofenadine	-0.299189584	0.157914297	1.36461416
Fenoxycarb	-1.34689581	0.19746489	0.237393297
Fenoprofen	-0.652096214	0.151559156	0.381067246
Fenoldopam	0.098779301	0.25557993	0.120386211
Fenhexamid	0.643269445	0.380564722	0.077873105
Fenamiphos	-0.320828392	0.336402026	0.515450242
Exemestane	0.041448517	0.920860155	0.036834357
Eupatoriochromene	0.536573342	0.106390564	0.135804098

Eupatilin	0.302696489	0.036963899	0.092076781
Eugenol	1.19034092	0.402606223	0.124274879
Etidronic acid	-0.390407162	0.068642733	0.107618147
Ethynodiol Diacetate	0.896311749	0.085146133	0.844333407
Ethylmethylacetic acid	0.213962609	0.427900342	0.070865174
Ethirimol	-0.143671918	0.546447488	0.085779354
Ethidimuron	0.114377718	0.803711054	0.000187107
Estrone sulfate	0.75570011	0.411136773	0.09435911
Estrone glucuronide	0.462446544	0.215124122	0.187540685
Erythromycin	0.418295285	0.141292551	0.308614563
Ergothioneine	-0.520941726	0.324013949	0.300655281
Epothilone B	-0.381826334	0.154268802	0.554575993
Epicatechin	-3.588063834	0.165779127	0.38516578
Enalaprilat	0.377050366	0.301054593	0.09502567
Emtricitabine	-0.119427169	0.614563822	0.074079503
Ellagic acid	-0.034325743	0.848872333	0.042141366
Efavirenz	0.279521423	0.663972949	0.032353087
Ecgonine	-0.228035143	0.592908278	0.031237235
D-Xylitol	-0.696879937	0.086927299	0.497684981
dTDP	0.37177968	0.0608938	0.234037351
D-Phenylalanine	0.287017779	0.056277662	1.509888727
D-Pantethine	-0.417437644	0.286205362	0.12635109
Doxazosin	-0.10736307	0.150580615	0.169248231
D-Ornithine	-0.231546524	0.297959589	0.145876188
Dopamine	0.044274027	0.909721317	0.01648684
Docosahexaenoic acid	-0.413438207	0.071476303	0.14175927
Docetaxel	-1.391997272	0.007131915	0.641912694
DNOC	0.382163869	0.27919301	0.372389181
DL-Homocystine	0.336401462	0.09187535	0.263052457
DL-Glycerol 1-phosphate	0.162401498	0.625729272	0.169935722
DL-alpha-Hydroxybutyric acid	-0.475801198	0.10533393	0.352470561
Diuron	0.197694893	0.478334327	0.086965005
Disulfiram	0.580547057	0.012660503	0.672186251
Dinotefuran	0.239058648	0.797669433	0.176144496
Dinoseb	0.34236929	0.125421419	0.129330416
Dimethylallylpyrophosphate	0.189338219	0.642221057	0.036241713
Dimethyl sulfone	0.126893684	0.114787745	2.338101425
Dimefuron	0.454991242	0.572589256	0.102959801
Dihydrotestosterone	-0.096064882	0.842773621	0.007460968
Dihydrostreptomycin	-1.173068045	0.094037851	0.160484718
Dihydromyricetin	-0.336685928	0.520445427	0.123875633
Dihydromethysticin	-0.228663696	0.618324643	0.045811724
Dihydrofolic acid	-0.080653883	0.830467778	0.025626561

Digitoxin	-0.029506328	0.894788064	0.016740424
Digalacturonate	0.71088552	0.137945997	0.177299521
Diflunisal	0.49387289	0.174720932	0.10710722
Diflubenzuron	-0.043012314	0.831687483	0.153548823
Diethylthiophosphoric acid	-0.01712197	0.924106469	0.023053092
Diethyl phthalate	-0.114834406	0.738331604	0.205709244
Diethofencarb	-0.369423865	0.361936849	0.089783438
Diethanolamine	0.059598792	0.468721885	0.528908527
Diclofenac	0.159346609	0.462543457	0.071712946
Dichlorphenamide	0.284791908	0.011515412	0.419447568
Dichlorophen	0.70768929	0.043725396	0.110283966
Dichlormid	1.522722695	0.351593642	0.176872904
Diazoxide	-0.118083812	0.695921133	0.027660895
Diaminopimelic acid	-0.74754287	0.258032616	0.254602019
Diacetoxyscirpenol	2.149745374	0.008693522	0.265905451
D-Gulono-1,4-lactone	-0.781201972	0.106525752	0.232072628
D-Glycero-D-galacto-heptitol	0.487845486	0.119196048	0.139830729
D-Glyceraldehyde 3-phosphate	-0.247080223	0.236423296	0.131228
D-Glutamine	-0.215407493	0.067502064	0.471189938
D-Glucose 1-phosphate	-0.136459889	0.780100314	0.177903727
D-Fructose 1-phosphate	0.961416182	0.0256009	0.255603066
Dethiobiotin	-0.282818191	0.347785236	0.114743029
Desmosterol	-5.702332146	0.324846932	2.208447733
D-Erythrose 4-phosphate	-0.405365346	0.699322869	0.165073231
Deoxyuridine-5'-triphosphate	0.704515477	0.008355944	0.522269652
Deoxyuridine	0.423748642	0.176777703	0.083131876
Deoxyinosine	-0.487796494	0.053025623	0.123615705
Deoxycytidine	-0.470035829	0.134782118	0.149018468
Deoxycorticosterone acetate	0.152810287	0.041070691	0.331022033
Deoxyadenosine	0.111511371	0.481747971	0.060347423
Delphinidin 3-glucoside	-0.081426117	0.725560697	0.038775635
Dehydroepiandrosterone sulfate	-0.55020597	0.320289384	0.123020006
Dehydroepiandrosterone	0.225372473	0.172623967	0.433867506
Dehydroascorbate	-0.225459095	0.200813768	0.131150096
Deacetylvindoline	-1.375149798	0.012180473	0.804812845
Dattelic acid	-0.030235743	0.913109496	0.011078831
Daphnoretin	0.162373026	0.235590759	0.147841755
Dantron	0.105542875	0.188636374	0.385111384
dAMP	-0.152015198	0.886183423	0.105435062
D-Alanyl-D-alanine	0.978840496	0.033170221	0.273870262
Daidzin	0.250414241	0.593859572	0.052741227
Daidzein	-0.105856481	0.900034198	0.044035129
Dacarbazine	-2.529560001	0.179725297	0.626313313

D-2-Hydroxyglutaric acid	-0.035294805	0.874713264	0.20884268
Cytosine	0.264887576	0.638947353	0.020184951
Cytarabine	-0.357956674	0.269403475	0.84409646
Cysteine-S-sulfate	0.104496888	0.663772215	0.063811176
Cymoxanil	0.913004048	0.003760402	3.937427177
Cymarin	-1.005556361	0.082355347	0.221148295
Cyclopentanone	0.262567623	0.004337768	0.391560269
Cyclic GMP	-1.15264825	0.291697341	0.197533606
Cyclic AMP	0.052769177	0.908285316	0.001914211
Cyclamic acid	0.181272553	0.62035568	0.076394065
CTP	0.443600978	0.162382074	0.08848627
Coumachlor	1.121270712	0.036935823	0.171885724
Corydaline	0.34422291	0.655267478	0.026691507
Cortisone acetate	-0.165838409	0.672967284	0.056824605
Cortisone	-3.196453393	0.019546404	1.458127422
Cortisol 21-acetate	3.471356702	7.22E-05	0.447022984
Corticosterone	0.34384268	0.45881171	0.132956879
Coronaridine	0.108776887	0.329566397	0.090464385
Coleonol	0.08842499	0.816953175	0.053620787
Coenzyme Q10	-0.154400214	0.447764453	0.041522873
CMP	-1.646496809	0.323755133	0.316991262
clothianidin	3.420414729	0.000742835	0.301038104
Clomipramine	-0.255636131	0.540432977	0.056527653
Clethodim	-0.399994333	0.121533474	0.119483498
Clerodin	-0.763557463	0.263160406	0.232394442
Clavulanate	-0.59455035	0.357622931	0.111435595
Citrulline	-0.042023531	0.795949867	0.096664551
Citrinin	0.14666214	0.609574989	0.186043596
Citric acid	0.508992445	0.013408111	4.337631714
Citicoline	0.796850246	0.009332362	0.315661767
Cis-zeatin	1.57342896	0.028861803	0.428075382
cis-11-Methyl-2-dodecenoic acid	0.389206615	0.261068347	0.093860157
Ciprofloxacin	1.527592944	0.004443753	0.864984447
Cimetidine	0.243171724	0.597783225	0.10078248
Cilastatin	0.001122601	0.997140552	0.007273483
Chorismate	0.581345812	0.000167385	0.363887812
Cholesterol sulfate	-0.518522817	0.432163031	0.222744229
Chlorpheniramine	0.000723357	0.9978049	0.049179823
Chloridazon	0.410843336	0.300533566	0.195582309
Chloramphenicol	0.232945381	0.074056691	0.550783369
Chloraminophenamide	0.298379018	0.29026931	0.170321665
Chitobiose	-0.449165366	0.095889877	0.074693851
Chicoric acid	0.464661712	0.005599374	0.166562203

Chalconaringenin	1.806144399	1.03E-06	0.861517116
Cetraxate	-0.247012115	0.737190834	0.028700655
Cerulenin	0.385716556	0.522169776	0.083182705
Cerivastatin	1.367735213	0.000989263	1.146888072
Cephalomannine	-0.777575194	0.064916809	0.249268146
Celecoxib	-1.541013526	0.000311061	1.086498749
Ceftibuten	-0.187215419	0.629101775	0.126884766
Cefotaxime	-0.117108433	0.670326029	0.135500669
Cefdinir	0.141337659	0.128867417	0.06060826
Cefazolin	0.721377327	0.045732729	0.163707608
Cefalotin	2.162859644	0.000144762	0.302125766
Cefadroxil	-0.112847773	0.831776126	0.034525029
CDP	0.235515586	0.4869353	0.267037167
Castanospermine	0.35562297	0.583627773	0.059587994
Carprofen	0.466049254	0.197590339	0.488883094
Carmine red	0.394193672	0.011982397	0.316602489
Carglumic acid	-0.432926445	0.206926697	0.389131763
Carboxin	0.207405204	0.382208722	0.176974504
Carbaryl	-0.751013975	0.180125749	0.361668391
Carbamoylaspartate	0.075240979	0.523704852	0.071924423
Caprylic acid	-0.463859019	0.072860759	0.316907784
Caproic acid	0.189088533	0.025403687	1.835180886
Capric acid	-0.544396996	0.441466961	0.169148937
Capecitabine	-0.145991506	0.578336939	0.033492189
Candesartan cilexetil	-0.313199217	0.461349102	0.059119395
Candesartan	0.843161936	0.283846841	0.098736057
Canavanine	0.148183385	0.589054815	0.08807469
Camalexin	0.060315039	0.773635598	0.07328391
Caffeine	0.066305572	0.903074266	0.010041936
Caffeate	-0.02015817	0.974839467	0.068799594
Butyryl-L-carnitine	0.729029755	0.161921811	0.139706433
Butabarbital	-0.028199024	0.723913134	0.039079353
But-3-enylglucosinolate	2.013822938	1.14E-05	0.472352245
BUFEXAMAC	0.133395676	0.499497018	0.06131002
Bufalin	-0.05564001	0.896874932	0.009670915
Bromofenoxim	-0.016812312	0.928868496	0.160067543
Bromacil	0.172774549	0.407417519	0.245303845
Brassinolide	0.598823993	0.212334286	0.348348304
Bovinocidin	-1.065209644	0.377800902	0.272447388
Boscalid	0.517767637	0.192020196	0.082683718
Boc-Asn	-0.567013854	0.083335651	0.191911402
Bithionol	-0.035001695	0.951519578	0.02381462
Bispyribac	0.022585246	0.928077152	0.041185882

Bis(4-nitrophenyl)phosphate	0.385416667	0.342096147	0.067901717
Biopterin	0.218060571	0.328404047	0.140816282
Biochanin A	0.442731343	0.205197289	0.451561618
Bilirubin	0.97005874	0.238235305	0.39381166
Bifenazate	0.20611373	0.504146696	0.08459376
beta-Thujaplicin	0.189034267	0.748941242	0.031474846
beta-N-Acetylglucosamine	1.006024156	0.001809446	0.323551207
Benzoin	-0.180966641	0.643337247	0.055589126
Bentazone	0.127316816	0.141246999	0.364926679
Bendroflumethiazide	0.582819628	0.003612857	0.107510464
Beauvericin	-0.241410857	0.225677963	0.18031633
Azathioprine	0.461332625	0.036278425	0.192673294
Azadirachtin A	-1.477108614	0.025352437	0.263761697
Azacitidine	0.076072998	0.619773493	0.18331828
Atrazine	0.201477243	0.422456587	0.088965601
Atovaquone	-0.232367035	0.422389237	0.351633507
Asymmetric dimethylarginine	0.136869138	0.44016167	0.081160837
Asperuloside	-1.154690986	0.026231803	0.212216041
ASCORBATE	0.133440839	0.559949186	0.18795602
Aripiprazole	0.910260618	0.145591727	0.418275808
Apramycin sulfate	-0.471928776	0.373677521	0.085472864
Apigenin	0.936431588	0.457369067	0.098067067
Antimycin A1	-0.069505333	0.871064224	0.013738604
Anisomycin	0.157668191	0.471084852	0.064684012
Androsterone glucuronide	0.385421852	0.169824109	0.147740896
Anastrozole	1.553526201	2.12E-05	0.857294212
Amoxicillin	0.06558531	0.781099224	0.112705671
Aminoadipic acid	0.235032026	0.568863184	0.325252282
Amidosulfuron	0.351161865	0.009270893	0.674759607
Alprostadil	-2.369870981	0.340181216	0.456529155
Alpha-Tocotrienol	-1.900507041	0.125718869	0.771657783
alpha-Oxo-benzeneacetic acid	-0.170850395	0.723665789	0.068954057
alpha-L-fucose 1-phosphate	-0.949073699	0.100872913	0.23603727
alpha-Isopropylmalate	1.352567744	0.034763981	0.307382479
alpha-Ergocryptine	0.326438679	0.008633491	0.365925187
alpha-D-Ribose 1-phosphate	0.069973001	0.788106026	0.060619384
Alpha-dimorphecolic acid	0.313572865	0.593445389	0.048885628
Allocystathionine	0.221773296	0.488567209	0.061794504
Allantoin	0.605032144	0.254169335	0.061275663
Aldosterone	0.38088215	0.663867716	0.093877423
Albendazole sulfone	-0.311349318	0.349420977	0.203732008
Albendazole	-0.32163438	0.43952136	0.208152984
Ajmalicine	0.152298923	0.734670755	0.036717487

Agnuside	-0.090452444	0.852407834	0.195024193
Aflatoxin G2	-0.441683971	0.452683831	0.066101031
ADP	-0.095935358	0.851509075	0.031446315
Adouetine X	0.567980812	0.184713858	1.717983441
Adipate	1.983691282	0.001030353	0.941898546
Adenosine phosphosulfate	0.004310326	0.971539995	0.008564105
Adenosine 5'-phosphate disodium	0.783560232	0.009782998	0.245351584
Adenosine 5-O-(3-thiodiphophate)	0.229928739	0.571861462	0.124404255
Adenosine 3',5'-diphosphate	-0.49674371	0.359995526	0.249750593
Adenosine	-1.217526944	0.117320596	0.280265488
Adenine	0.191389898	0.056643942	0.221556027
Aclacinomycin A	0.758737135	0.002358566	0.448544144
Aciclovir	1.791804521	0.00291244	0.474143037
Acetylisoniazid	0.209788895	0.199619944	0.118627978
Acetylcysteine	1.588564657	0.025934543	0.429354828
Acetosyringone	1.04275368	0.077073401	0.36323809
Acetohexamide	1.198160442	0.079364234	0.19200201
9-OxoODE	0.005585454	0.99089181	0.042963508
7,8-Dihydroneopterin	0.279778892	0.424194471	1.548077914
6-Thiourate	-0.200356923	0.679806708	0.053250799
6-Methylmercaptopurine	1.006054459	0.00027478	0.39896666
6''-Malonylgenistin	-0.319446814	0.006132761	0.684599155
6-Hydroxymelatonin	0.398207901	0.51943163	0.099069747
6-Hydroxyhexanoic acid	0.040004174	0.952700531	0.098757134
6beta-Hydroxytestosterone	1.871907896	0.030115929	0.606563464
6-Aminopenicillanic acid	0.185569346	0.158581281	0.136356858
5'-Methylthioadenosine	0.015910382	0.981035136	0.006174992
5-Methyltetrahydrofolic acid	-4.844726356	0.10074669	0.586151529
5-Methyl-2'-deoxycytidine	-2.512819291	0.197760417	0.280432989
5-Methoxyindoleacetate	-0.510325064	0.487245236	0.188912006
5-Hydroxy-L-tryptophan	0.282521964	0.06821151	0.201486529
5,6-DHET	0.225822501	0.64948288	0.103061334
4-Nonylphenol	-0.207597215	0.445658313	0.046355742
4-Nitroquinoline N-oxide	-0.032921088	0.904431083	0.002752088
4-Nitrophenyl phosphate	0.015446123	0.940476892	0.021502924
4-Methylumbelliferyl acetate	-0.149454509	0.898147292	0.094950628
4-Methylumbelliferone sulfate	-0.077955507	0.899493048	0.220159044
4-Methylcatechol	1.394837263	0.212986165	0.542870272
4-Hydroxytamoxifen	-0.309467417	0.450568221	0.130619604
4-Hydroxyproline	0.139175458	0.374909124	0.168327062
4-Hydroxycinnamyl alcohol 4-D-glucoside	0.172148776	0.757542399	0.028717977
4-Hydroxycinnamoylagmatine	-0.181101127	0.562333181	0.064149904
4-Hydroxycinnamic acid	0.645445558	0.107030241	0.228864616

4-Hydroxybenzeneacetonitrile	0.759022696	0.118258979	0.126363531
4-Hydroxy-3-methylbenzoate	-3.220210229	0.155409293	1.178819206
4-Hydroxy-3-(3-methyl-2-butenyl)acetophenone	-1.024499031	0.032665087	0.274782724
4-fluorobenzoate	0.274746033	0.216493959	0.135296232
4-Ethylphenol	-0.226486857	0.734981543	0.064474308
4-Bromophenol	0.10307965	0.235826253	0.824684568
4-Acetamidobutanoic acid	-0.158853638	0.215872611	0.236504243
4,4'-Sulfonyldiphenol	-0.095453045	0.722507665	0.019415152
4-(beta-D-Glucosyloxy)benzoate	-0.007231979	0.952458946	0.003343415
3-Sulfinoalanine	1.057346069	0.002859967	0.387785223
3-O-alpha-Mycarosylerythronolide B	-0.049079395	0.834216153	0.061948194
3-Methylxanthine	-0.430940092	0.051986587	0.339701929
3-Methylthiopropionic acid	0.87460533	0.035908318	0.12981517
3-Methylguanine	0.155839736	0.278436895	0.127107703
3-Indoleacetonitrile	-0.591557333	0.301741482	0.120572192
3-Hydroxyvalproic acid	-0.298106989	0.410206609	0.180319627
3-Hydroxybenzo[a]pyrene	0.512999855	0.150538261	0.454623989
3-Hydroxyanthranilate	0.429918024	0.056078419	0.460397695
3-Deoxy-lyxo-heptulosaric acid	-1.787162105	0.330018868	0.720008631
3-Amino-3-(4-hydroxyphenyl)propanoate	0.234173299	0.00880341	0.501080384
3,7-Dimethyluric acid	-0.939481532	0.297806535	0.63186
3,5-Dinitrosalicylic acid	0.314982717	0.252187193	0.12305055
3,5-Diiodo-L-tyrosine	0.209165239	0.119538274	1.024813468
3,5-Dibromo-4-hydroxybenzoate	-0.105527207	0.902887133	0.000775582
3',5'-Cyclic dAMP	0.151162702	0.644372192	0.135328393
3-(Uracil-1-yl)-L-alanine	-0.050704213	0.773974786	0.037552215
3-(4-Hydroxyphenyl)lactate	0.154263435	0.127594148	0.104217963
3-(4-Hydroxyphenyl)-1-(4-hydroxy-2-methoxyphenyl)-2-propen-1-one	0.227609208	0.378787225	0.524242765
3-(3,4-Dihydroxy-5-methoxy)-2-propenoic acid	-2.183524221	0.143541995	1.39423295
2-Pyrocatechuic acid	-0.474451382	0.328846341	0.146888086
2'-O-Methyladenosine	0.538968428	0.112475248	0.281592422
2-Methoxy-4-vinylphenol	-0.528786122	0.501288294	0.17657226
2-Ketobutyric acid	0.092839511	0.446834565	0.338216543
2-Hydroxyestradiol	0.477738357	0.374008944	0.369108708
2-Heptanone	-2.271839333	0.003993061	11.94812505
2-C-Methyl-D-erythritol 2,4-cyclodiphosphate	-0.759127193	0.042585929	0.168536083
2-Benzimidazolylguanidine	0.335726112	0.427560105	0.108774178
2-Arachidonylglycerol	0.404221133	0.35319481	1.845627503
2-Aminoethylphosphonate	-0.841473606	0.161081728	0.400912758
2-Amino-3-methylimidazo[4,5-f]quinoline	-0.417100778	0.080029678	0.173241955
2-Amino-2-deoxy-D-gluconate	0.845290169	0.031096972	0.254580354
25-Hydroxycholesterol	-0.386293699	0.004894932	1.756873817
20-HETE	0.193174357	0.823595488	0.049404307

2,6-Dimethoxy-1,4-benzoquinone	-0.032992922	0.813578309	0.118674616
2',6'-Dihydroxy-4'-methoxyacetophenone	0.285877148	0.154335595	0.940759555
2,4-DP	-0.10677022	0.762637332	0.043237694
2,4-Dinitrotoluene	-0.037465656	0.890061761	0.045193072
2,4-Dihydroxypteridine	0.854861439	0.02732505	0.427477168
2',4'-Dihydroxyacetophenone	0.499336594	0.001409602	0.902422279
2,4-Dichlorophenoxyacetic acid	-0.042711705	0.845811819	0.051463155
2,4-Dibromophenol	2.797210312	0.302878691	1.134400368
2,4-DB	0.651073056	0.195094437	0.12063038
2,4,5-Trichlorophenol	0.200049044	0.394499256	0.104791919
2,3-Butanediol	0.273698984	0.255161779	0.074637289
2,3-Bisphosphoglycerate	0.074966855	0.564208175	0.771120438
2,2-Bis(4-hydroxyphenyl)hexafluoropropane	0.223505007	0.088979747	0.175783303
2-(Methylamino)benzoic acid	-0.102887203	0.779375342	0.031811867
1-Phospho-alpha-D-galacturonate	-1.110677776	0.159780911	0.140702863
1-palmitoylglycerophosphocholine	0.223348002	0.334975591	0.272701643
1-O-Galloyl-beta-D-glucose	0.880332448	0.022480553	0.211372374
1-Methylxanthine	0.586017775	0.259440242	0.756700708
1-Methyluric acid	0.011983244	0.989383915	0.153828618
1-Methyladenosine	0.975945716	0.007691623	0.591392952
1-Methyl-6-phenyl-1H-imidazo[4,5-b]pyridin-2-amine	0.030029271	0.819762424	0.042606086
1-Hydroxypyrene	0.304386094	0.459380879	0.187652359
1H-Indole-3-acetamide	0.40281336	0.038983344	0.15754106
1H-Indole-2,3-dione	1.479386851	0.017997857	0.588289991
1-Hexadecanol	0.211462577	0.150808602	0.093127007
1D-Myo-inositol 1,4,5,6-tetrakisphosphate	-0.438591225	0.442606069	0.242127346
17a-Ethynylestradiol	-0.066322237	0.806749251	0.101709619
16-Hydroxy hexadecanoic acid	-0.1927625	0.649155134	0.170071787
16alpha-Hydroxyestrone	1.517046449	0.144626088	0.125963012
13-OxoODE	-0.238961737	0.524587377	0.373257003
12-Keto-tetrahydro-leukotriene B4	0.225844219	0.658894591	0.18377932
1,8-Dinitropyrene	-0.458587007	0.401602863	0.122708227
1,5-Anhydro-D-glucitol	-0.379440992	0.209617576	0.361497763
1,3-Benzenediol	-0.642146677	0.059062475	0.250940888
1,2,6-Trigalloyl-beta-D-glucopyranose	-0.386257923	0.044700498	0.414506401
[8]-Shogaol	0.952538423	0.015031511	0.912319904
?-Cyclodextrin	-0.004489284	0.987917784	0.021986114
(S)-Isocorydine	0.157946015	0.281951346	0.146038308
(S)-[10]-Gingerol	1.131648933	0.089334499	0.489547943
(R)-Mevalonate	0.166788917	0.561251852	0.121468673
(R)-Kawain	-0.154995379	0.585261025	0.052583216
(2R)-2-Hydroxybut-3-enylglucosinolate	-1.62156668	0.138968642	0.596187663
(2-Naphthalenyloxy)acetic acid	0.533158107	0.164004984	0.644721242

(+)-Syringaresinol	-1.468717496	0.003280912	0.285762892
(+)-Eudesmin	-0.247697526	0.637692462	0.103685199
(+)-Aschantin	0.368455549	0.139351718	0.12128538
(-)-Wikstromol	-0.077649356	0.731532115	0.037955898
(-)-Jasmonic acid	-2.388503193	0.085660325	1.498047665
(-)-Epigallocatechin	-0.168616802	0.480376443	0.510185209
